# Harnessing innate immune pathways for therapeutic advancement in cancer

**DOI:** 10.1038/s41392-024-01765-9

**Published:** 2024-03-25

**Authors:** Ankang Hu, Li Sun, Hao Lin, Yuheng Liao, Hui Yang, Ying Mao

**Affiliations:** 1 Department of Neurosurgery, Huashan Hospital, Fudan University, Shanghai, P.R. China; 2 Institute for Translational Brain Research, Shanghai Medical College, Fudan University, Shanghai, P.R. China; 3 National Center for Neurological Disorders, Huashan Hospital, Shanghai Medical College, Fudan University, Shanghai, P.R. China; 4 Shanghai Key Laboratory of Brain Function Restoration and Neural Regeneration, Shanghai Clinical Medical Center of Neurosurgery, Neurosurgical Institute of Fudan University, Huashan Hospital, Shanghai Medical College, Fudan University, Shanghai, P.R. China; 5 State Key Laboratory of Medical Neurobiology and MOE Frontiers Center for Brain Science and MOE Frontiers Center for Brain Science, Institutes of Brain Science, Shanghai Medical College, Fudan University, Shanghai, P.R. China; 6 Shanghai Key Laboratory of Medical Epigenetics, International Co-laboratory of Medical Epigenetics and Metabolism (Ministry of Science and Technology), and Key Laboratory of Metabolism and Molecular Medicine (Ministry of Education), and Molecular and Cell Biology Lab, Institutes of Biomedical Sciences, Shanghai Medical College of Fudan University, Shanghai, P.R. China

**Keywords:** Cancer, Oncology

## Abstract

The innate immune pathway is receiving increasing attention in cancer therapy. This pathway is ubiquitous across various cell types, not only in innate immune cells but also in adaptive immune cells, tumor cells, and stromal cells. Agonists targeting the innate immune pathway have shown profound changes in the tumor microenvironment (TME) and improved tumor prognosis in preclinical studies. However, to date, the clinical success of drugs targeting the innate immune pathway remains limited. Interestingly, recent studies have shown that activation of the innate immune pathway can paradoxically promote tumor progression. The uncertainty surrounding the therapeutic effectiveness of targeted drugs for the innate immune pathway is a critical issue that needs immediate investigation. In this review, we observe that the role of the innate immune pathway demonstrates heterogeneity, linked to the tumor development stage, pathway status, and specific cell types. We propose that within the TME, the innate immune pathway exhibits multidimensional diversity. This diversity is fundamentally rooted in cellular heterogeneity and is manifested as a variety of signaling networks. The pro-tumor effect of innate immune pathway activation essentially reflects the suppression of classical pathways and the activation of potential pro-tumor alternative pathways. Refining our understanding of the tumor’s innate immune pathway network and employing appropriate targeting strategies can enhance our ability to harness the anti-tumor potential of the innate immune pathway and ultimately bridge the gap from preclinical to clinical application.

## Introduction

The emergence of immunotherapy has revolutionized the paradigm of cancer treatment with immune checkpoint blockers (ICB). These monoclonal antibodies, blocking immune checkpoint-relevant molecules such as PD1, PD-L1, and CTLA-4, exert their effect by inhibiting the immunosuppressive signals in T cells, thus amplifying T cell-mediated tumor cytotoxicity. While the utility of ICB has ushered in positive outcomes across multiple cancer treatments, their effectiveness largely hinges on the presence of cytotoxic T cells capable of eradicating cancer cells within the tumor.^1^ Unfortunately, not all tumors boast an adequate presence of these potent cells for effective regression. Based on immune characteristics, tumors are classified into four main categories — hot, altered-excluded, altered-immunosuppressed, and cold, representing high T cell infiltration, T cell peripheral infiltration without tumor invasion, T cell infiltration with immunosuppression, and lack of T cell infiltration, respectively. Compared to hot tumors, cold tumors inherently lack T cell infiltration, and tumors with altered phenotypes cannot harness T cell functionality due to spatial or functional constraints.^2^ For cold tumors, such as glioblastomas and pancreatic cancer, ICB has not yielded significant breakthroughs.^3,4^ Improving outcomes for patients with these types of tumors remains an urgent challenge.

The innate immusne pathway, an integral component of the immune system, plays a pivotal role in immune system activation. A hallmark of the innate immune response is its “broad” scope. This is evident in its widespread presence across various cell types and its ability to secrete interferons (IFNs) and a myriad of cytokines, altering the overall immune landscape.^5^ Increasing evidence suggests that immune cells exhibiting an immunosuppressive phenotype still retain their anti-tumor potential, and effective interventions can stimulate their reprogramming toward an antitumor phenotype.^6–8^ The expansive effects of the innate immune pathway can counteract the suppressive nature of the TME, providing potent signaling reinforcements for cellular reprogramming.^9,10^ Moreover, the activation of the innate immune pathway plays a pivotal role in the maturation, antigen presentation, and expression of co-stimulatory molecules in antigen-presenting cells (APCs), which is an indispensable step for APCs to activate T cells.^9,11^

Despite demonstrating significant efficacy in preclinical trials, targeted drugs for the innate immune pathway have achieved only limited success in clinical settings. Moreover, some studies have reported that downstream signaling of the innate immune pathway may contribute to tumor promotion.^12,13^ Identifying the reasons behind the uncertain therapeutic effectiveness of targeted drugs for the innate immune pathway is an urgent matter that requires exploration.

In this review, we have delved into the immune status of diverse immune cells present within the TME, with a spotlight on their metabolic-mediated immunosuppressive transformation and the prospects for antitumor reprogramming. We have further elucidated the roles of prevalent innate immune pathways, examining their impact on tumor onset, development, and metastasis and their heterogeneous functions across various cells in the TME, as well as their misregulation in oncological contexts. The discussion on this topic presents two core views: the potential of innate immune pathways to reprogram immunosuppressive cells into anti-tumor cells, and the aberrant shaping of innate immune pathways in the TME leading to the instability and unreliability of singular agonist applications. We have summarized the promising anti-tumor results of innate immune agonists in existing clinical trials and their established safety profile, collated and categorized the application strategies of existing innate immune pathway agonists, and emphasized the prospects of targeting innate immune pathways and the importance of combination multi-targeted therapies in overcoming abnormal tumor signaling pathways. Finally, we discussed how to overcome the barriers to targeted therapy of innate immune pathways in the future. The core perspective is to fully dissect the plasticity and heterogeneity of innate immune pathways from multiple dimensions, focusing on and revolving around the treatment paradigm of “precision medicine.”

## The immune landscape in the tumor microenvironment

**Fig. 1:**
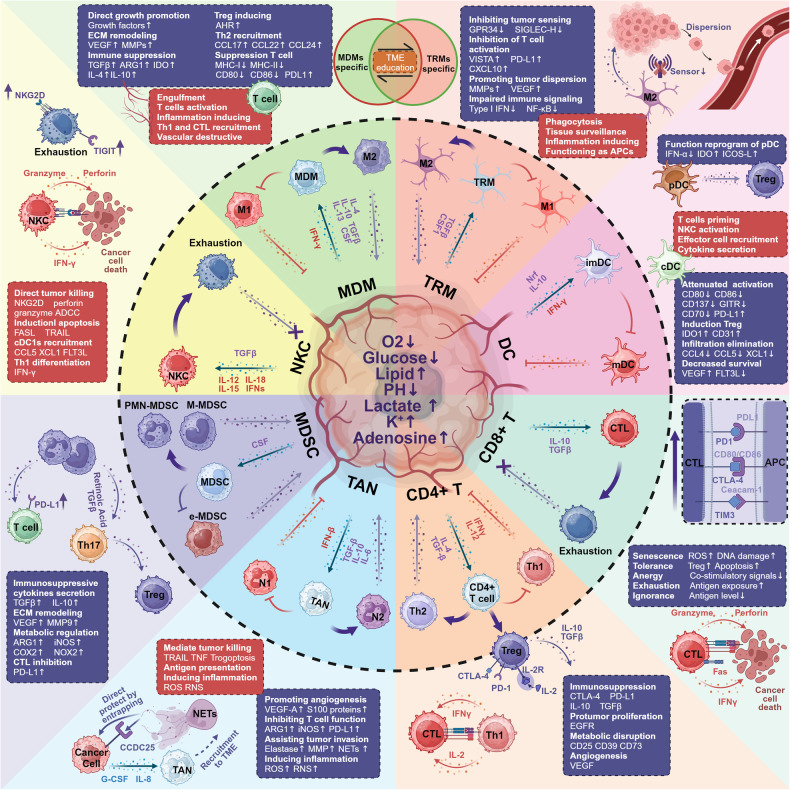
Tumor immune microenvironment. The TME mainly includes monocyte-derived macrophages (MDMs),^655,656^ tissue-derived macrophages (TRMs),^655^ dendritic cells (DCs),^77^ CD8^+^T lymphocytes (CTLs),^124^ CD4^+^ T lymphocytes,^149,166^ tumor-associated neutrophils (TANs),^657^ myeloid-derived immunosuppressive cells (MDSCs),^658^ and natural killer cells (NKCs).^111^ Cells in the tumor immune microenvironment have pro-tumor (purple) and anti-tumor (red) effects. The TME induces immune cells’ pro-tumor differentiation or polarization through various pathways. The abnormal microenvironment of a tumor (black text in the tumor’s center), along with factors (purple) secreted by the tumor itself, can induce cells within this environment to undergo differentiation or polarization towards a pro-tumor phenotype, or experience exhaustion of their anti-tumor functions. However, there are cytokines (red) that have the potential to overcome or reverse these pro-tumor phenotypes. Created with BioRender.com

The immune cells within the TME are critically important for both the progression and treatment of cancer. In this section, we primarily explore how the TME developmentally, functionally, and metabolically remodels innate immunity, as well as how innate immune cells can be targeted to overcome immunosuppressive states. We provide a general outline for each class of immune cells (Fig. 1).

### Tumor-associated macrophages

Tumor-associated macrophages (TAMs) are pivotal immune cells mediating chronic inflammation within TME and are ubiquitous across all tumor types.^14,15^ A salient characteristic of macrophages is their polarization, endowing them with anti-inflammatory and pro-inflammatory capabilities and building upon the classification of helper T cells (Th) 1 and 2, Mills et al. devised a typology dividing macrophages into anti-tumor M1-type and pro-tumor M2-type.^16^

During the generation of tumors, M1-type macrophages play roles in immune surveillance and eradication.^17,18^ They boast robust phagocytic activity and antigen-presenting capabilities, activating T cells and releasing cytokines that promote inflammation. However, as tumors evolve, there’s a shift towards M2 polarization, driven by the tumor milieu. Th2 cells, basophils, and eosinophil release inducers such as IL-4, IL-13, and tumor cells themselves release cytokines such as macrophage colony-stimulating factors (CSFs) and transforming growth factor (TGF) β that drive this M2 polarization.^19–23^ The tumor’s hypoxic conditions and lactic acid presence are also involved in this process. Hypoxia induces TAMs to upregulate the expression of SLC40A1 (solute carrier family 40 member 1) and lipocalin 2, which promotes the supply of iron to tumor cells, consequently facilitating tumor proliferation.^24,25^ Hypoxia also influences TAMs’ glucose metabolism, contributing to tumor growth, although the specific mechanisms remain unclear. On one hand, hypoxia upregulates DDT4 (DNA damage-inducible transcript 4), driving the inhibition of the key glycolytic entity, mechanistic target of rapamycin (mTOR) complex 1 (mTORC1). This metabolic shift from Warburg respiration (high glycolytic metabolism) to mitochondrial respiration reduces glucose uptake, increasing relative glucose availability for tumor cells and endothelial cells.^26^ On the other hand, some studies have found that hypoxia increases glucose transporter 1 (GLUT1) expression in TAMs, enhancing glucose uptake and glycolysis. This, in turn, suppresses antitumor immunity by competing for glucose with NK cells and T cells.^27^ Additionally, excessive glycolysis in the TME, resulting in an accumulation of lactate, can lead to histone lactylation in TAMs, driving the transition of TAMs from an M1 to an M2 phenotype.^28^

The TME also induces the enhancement of fatty acid biosynthesis, uptake, storage, and oxidation of TAMs through mechanisms that are not yet fully understood.^29^ This metabolic characteristic is associated with the functional transition of TAMs. The enhancement of lipid metabolism in TAMs not only supports their energy supply but also contributes to shaping an acidic TME through the production of various fatty acid derivatives like PGE2 (Prostaglandin E2) and ketone bodies. This acidic environment is known to suppress anti-tumor immunity.^30,31^

Although M2-type TAMs form a positive feedback loop with TME, this loop could be broken by re-education of TAMs into the M1 phenotype. The application of monoclonal antibodies like anti-CD20 and anti-HER2 antibodies can let macrophages exert antibody-dependent cellular cytotoxicity (ADCC), which underpins the anti-tumor activities of these drugs.^18,32^ Additionally, several interventions, including the activation of IRF (IFN Regulatory Factor) 7, blocking CD47 on tumor cells which interact with SIRPα (signal-regulatory protein α) mediating the “don’t eat me” signal, and inhibiting immune checkpoint molecule PD-L1, have shown promise in reprogramming TAMs towards an anti-tumor phenotype.^33–36^

In conclusion, while the TAMs predominantly present an immunosuppressive profile educated by the TME, they still harbor anti-tumor potential. Exploring ways to harness this latent capability of TAMs to counteract tumors is enticing and warrants further investigation.

#### Monocyte-derived macrophages and tissue-resident macrophages

TAMs consist of monocyte-derived macrophages (MDMs) and tissue-resident macrophages (TRMs).^37^ MDMs are recruited from circulating monocytes into the TME, where they differentiate upon being influenced by the tumor milieu. For instance, recruited monocytes in breast cancer were identified to differentiate into TAMs depending on the Notch signaling pathway activated under the TME.^38^ Postnatally, MDMs can continually replenish and renew from hematopoietic stem cells in the bone marrow while maintaining and expanding their population through self-proliferation.^38^ In many rodent tumor models, MDMs dominate the TAM population.^18,38,39^ However, despite being the primary group within TAMs, MDMs often fail to exhibit an effective anti-tumor response.^40^ Typically, their level of infiltration correlates with a poor prognosis in patients.^41^

In contrast to MDMs, TRMs originate from early yolk sac progenitors, boasting a comparatively longer lifespan. They maintain population homeostasis through self-renewal rather than relying on monocytes.^42^ TRMs, guided by specific tissues, acquire tissue-specific functions.^43^ All TRMs exhibit elongated protrusions morphologically, monitoring the homeostatic state of their respective tissues.^43^ Given the tissue adaptability and surveillance functions of tissue macrophages, they are perceived as sentinels watching tumor initiation, capable of restraining tumor growth. For example, Kupffer cells in the liver have been found to inhibit liver tumor metastasis through phagocytosis.^44^ However, evidence also suggests that resident cells can support tumor progression. In triple-negative breast cancer (TNBC), depletion of TRMs significantly reduces tumor growth, recurrence, and metastasis.^45^ In pancreatic ductal adenocarcinoma (PDAC), TRMs promote tissue fibrosis and shape the pro-tumor extracellular matrix.^46^ Such evidence indicates that there remain many disputes regarding the roles and mechanisms of TRMs in tumors.

#### Microglia

Microglia are among the most extensively studied TRMs. Research on them has revealed that they play a dual role in the development of gliomas, similar to that of MDMs. And through microglia, we discern the importance of regulating TRMs to curtail their tumor-promoting transformation.

As early as the 1920s, it was discovered that microglia could engulf glioma cells.^47^ Microglia can also respond to stimulation of the TME, be activated to transform into an amoeboid shape, and express major histocompatibility complex (MHC) class I (MHC-I) and MHC-II, as well as co-stimulatory molecules such as CD86, functioning as APCs to activate T cells.^48^ However, the glioma environment can induce microglia to transition into a pro-tumor phenotype. Indeed, analysis of human glioma samples reveals a downregulation in the expression of receptors used by microglia to sense their surroundings, and this downregulation is more pronounced in the tumor core.^49,50^ Glioma cells themselves also participate in the remodeling process of microglia. Research has found that a subset of microglia that have engulfed glioma-derived extracellular vesicles show significant downregulation of genes involved in sensing tumor cells, tumor danger signals, and tumor-killing, such as SIGLEC-H and the GPR34 (G protein-coupled receptor 34). In contrast, genes promoting tumor dispersion are significantly upregulated, including the upregulation of the immune-checkpoint protein PDL1 and multiple MMP-encoding genes.^50,51^ Single-cell transcriptomic analyses from different datasets reveal that, compared to normal controls, gliomas contain two clusters of microglia that are absent in the rules. In these two clusters, the expression of CX3CR1 and SELPLG (CD162) in microglia is significantly downregulated.^49,52^ CX3CR1 and SELPLG act as the glioma sensors that promote microglial infiltration into the tumor.^53,54^ In addition, compared to healthy controls, cluster data from the glioma group revealed higher expression of hypoxia-related genes such as hypoxia-inducible factor 1α (HIF-1α) and VEGF α.^52^

Moreover, Gene Ontology analysis indicates a “positive regulation of vasculature development.” Concurrently, microglia display a transcriptomic phenotype resembling that of age-related microglia.^52^ Specific microglial clusters also exhibit enhanced anti-inflammatory CXCL10 secretion and enriched expression of T-cell inhibitory molecules such as VISTA and PDL1.^55^ In addition, microglia in the glioma environment show impaired type I IFN signaling and NF-κB signaling.^56^ These findings all indicate the impairment of the anti-tumor immune function of microglia in the glioma environment.

#### MDMs and TRMs: similar but different

While MDMs and TRMs display similarities in many aspects, they occupy distinct ecological niches and serve different roles.

MDMs and TRMs differ in tissue localization. In glioma, MDMs predominantly localize around blood vessels and necrotic regions, demonstrating a higher adaptability to hypoxia. In contrast, microglia primarily infiltrate the tumor’s periphery.^57^ Furthermore, MDMs and TRMs exhibit distinct functions. In PDAC, TAMs are predisposed to shape immune responses, while TRMs tend to shape the extracellular matrix.^46^ In lung cancer, TAMs primarily facilitate tumor metastasis, whereas TRMs mainly support tumor growth.^58^ TRMs manifest a more pronounced M2 phenotype in breast cancer than MDMs.^38^ In brain tumors, microglia show a stronger inclination towards pro-inflammatory functions compared to MDMs.^59,60^ MDMs, on the other hand, have a more pronounced tendency to interact with endothelial cells, hinting at their role in angiogenesis.^61^ Moreover, the composition ratio of MDMs to TRMs in the TME differs. While MDMs often dominate, the balance can reverse in specific scenarios. For instance, in early-stage TNBC, TRMs are the primary TAMs.^45^ A similar pattern is seen in glioblastoma multiforme (GBM); in newly diagnosed cases, microglia dominate, but in recurrent glioblastoma, MDMs outweigh microglia, especially in hypoxic areas.^62^ In IDH mutant gliomas, microglia are predominant, whereas, in IDH wild-type gliomas, the situation is reversed with MDMs surpassing the microglia.^56,63^

Interestingly, MDMs and TRMs compete for ecological niches. For instance, in brain tumors, by using CCR2-KO to reduce monocyte migration toward tumors, it was noted that the microglia population increased.^64^ Conversely, when microglia are depleted, monocytes fill the microglia’s ecological niche and adopt related phenotypic traits.^65^ The characteristics of TAMs in the TME are determined by the cell’s genetic background, the tumor microenvironment, and the tissue environment.^16^ This suggests a potential for MDMs and TRMs to transform into each other. Single-cell sequencing results have shown that a subset of MDMs acquires a microglia-like phenotype, such as the synapse pruning function.^64^ Consequently, the TME shapes unique immune cells, and dissecting the immunological function of TAMs requires more detailed analyses. This is crucial for the implementation of cell-type-specific targeted drugs.

Current evidence underscores the diverse roles of TAMs from different origins at various stages of tumor progression, suggesting potential interconversion and complementary functions. Accurate lineage tracing of TAMs’ origin can illuminate the tumor development process. This area, rich in potential, forms the foundation for implementing cell-type-specific precision therapies.

### Myeloid-derived suppressor cells

Myeloid-derived suppressor cells (MDSCs), a group of immune-suppressive cells, play a crucial role in promoting tumor growth and protecting tumors from immune recognition in cancer patients. MDSCs primarily bifurcate into two dominant categories: granulocytic or polymorphonuclear MDSCs (PMN-MDSCs), originating from the granulocyte lineage, and monocytic MDSCs (M-MDSCs), stemming from the monocyte lineage. Moreover, a lesser-known subpopulation of MDSCs, characterized by traits of bone marrow progenitors and precursors, is termed early immature MDSCs (e-MDSCs).^66^ MDSCs in the tumor undergo metabolic reprogramming. In the TME, glycolytic genes and the rate of glycolysis in MDSCs are upregulated, producing large amounts of phosphoenolpyruvate (PEP). PEP acts as an antioxidant agent, blunting the production of reactive oxygen species (ROS) to avoid ROS-mediated apoptosis and supporting MDSC survival.^67^ Furthermore, the abundant lactate produced by the tumor’s Warburg metabolism promotes the accumulation of MDSCs in the TME.^68^ MDSCs, induced by tumor-derived G-CSF and GM-CSF, activate STAT3 and STAT5, which induce the expression of lipid transport receptors, enhancing the uptake of liposomes, boosting respiration, and providing energy support for their immune suppression.^69^ Furthermore, the hypoxic environment in tumors induces the upregulation of HIF-1α in MDSCs, which promotes PD-L1 expression through the direct binding of HIF-1α to a transcriptionally active hypoxia-response element (HRE) in the PD-L1 proximal promoter, exerting immune suppression.^70^ However, although we know that the metabolic reprogramming of MDSCs contributes to their immune-suppressive function, the specific pathways by which they exert influence require further research.

Interestingly, the TME can skew the MDSC population distribution. For instance, in GBM patients, besides an overall surge in MDSC quantity compared to healthy controls, there’s a conspicuous shift with an increase in PMN-MDSCs and M-MDSCs and a significant decrease in e-MDSCs.^71,72^ Peripheral and tumor tissue MDSCs can further differentiate, exemplified by M-MDSCs differentiating into PMN-MDSCs in tumor-bearing mice through histone deacetylase 2 (HDAC-2) mediated epigenetic silencing of the retinoblastoma gene (Rb1).^73^ This population shift further amplifies immunosuppression. The differential regulatory role of MDSCs extends to the T cell populations. For example, TGF-β and retinoic acid, derived from MDSCs, can facilitate the conversion of T helper 17 (Th17) cells into regulatory T (Treg) cells.^74^ This process transforms T cells from a pro-inflammatory phenotype into an anti-inflammatory phenotype

Despite their pronounced immunosuppressive role, MDSCs remain plastic with the potential for immune functional shifts. In vivo experiments with all-trans retinoic acid (ATRA) have demonstrated significant differentiation of MDSCs into mature myeloid cells, leading to reduced MDSC presence and counteracting MDSC-mediated immune suppression.^75^

### Dendritic cells

Dendritic cells (DCs) are unique APCs instrumental in instigating and modulating both innate and adaptive immune responses.^76^ However, the TME can attenuate the anti-tumor immune functions of DCs, especially regarding T-cell anti-tumor functions. For instance, the TME can downregulate DC co-stimulatory molecule expression, leading to T cell immune paralysis without co-stimulatory molecule activation.^77^ Lipid metabolism DCs play a crucial role in this process. The TME compels DCs to upregulate scavenger receptors, thereby increasing lipid uptake.^78^ DCs with high lipid content are unable to effectively stimulate allogeneic T cells or present tumor-associated antigens, leading to impaired T cell-mediated anti-tumor immunity. Besides, extracellular vesicles from GBM stem cells, like LGALS9, can hamper DC antigen presentation.^79^ Furthermore, tumor-infiltrating DCs may elevate the presentation of immune-inhibitory molecules like PD-L1.^80^ Monocytes in the TME can diminish DC antigen presentation through paracrine mechanisms.^81^

Stressors from the tumor can skew the DC-driven T-cell differentiation towards a tumor-promoting phenotype. For instance, upregulation of CD31 on DCs can tilt Th differentiation towards Tregs.^82^ Furthermore, DCs can stifle CD8^+^ T cells and natural killer (NK) cell proliferation and aid Treg cell differentiation by expressing IDO1, which degrades essential substrates for immune cell responses.^83^ The tumor milieu can also impede the anti-tumor capability of Plasmacytoid DCs (pDC). The TME impairs the production of type I IFNs in pDCs by relocating TLR9 to late endosomal compartments and bolstering their proclivity to activate Treg cells via the ICOSL (inducible T cell costimulator ligand).^84,85^ Beyond inhibiting the function of DCs, tumors also suppress the recruitment, maturation, and differentiation of DCs within the TME. The TME diminishes the recruitment of DCs by reducing the expression of chemotactic factors such as CCL4, CCL5, and XC-chemokine ligand 1 (XCL1), effectively excluding DCs from the TME. The TME can also evoke the overexpression of Nrf, a redox-sensitive transcription factor, inhibiting DC functional maturation.^86^ Tumor-derived VEGF can inhibit the activity of FMS-related tyrosine kinase 3 ligand (FLT3L), crucial for in-situ DC development and survival.^87^ Low extracellular nutrients and a high AMP/ATP ratio in the TME can activate AMP-activated protein kinase (AMPK) in DCs, leading to the expression of tumor-promoting factors such as VEGF, TGF-β, and IL-10.^88^ Other cell types also modulate the DCs. For example, Treg cells upregulate IL-10 expression when type I IFN declines in the TME, inhibiting the mature status of DCs and limiting their pro-inflammatory cytokine production.^89^

In a word, DCs harbor potent anti-tumor potential, the TME re-educates them, causing immune desensitization, even morphing them into tumor-promoting phenotypes.

### Neutrophils

Neutrophils are the most prevalent immune cells in the bloodstream and have a relatively short lifespan.^90^ There is substantial evidence suggesting that tumor-associated neutrophils (TANs) play a dual role in cancer, both promoting and inhibiting tumor growth according to this, Fridlender et al. categorized TANs into the anti-tumor N1 type and the pro-tumor N2 type.^91^ However, in most tumors, the extent of neutrophil infiltration is associated with a poor prognosis.^92^ Tumor cells induce the pro-tumoral transformation of neutrophils by secreting cytokines. For example, tumor cells induce PD-L1 expression in TANs by secreting GM-CSF and stimulate higher levels of iNOS and ARG1 expression in TANs through TGF-β.^93^^,94^ Additionally, the aberrant TME reshapes the energy metabolism characteristics of TANs, affecting downstream transcriptional and translational processes. Hypoxic TME upregulates the HIF1α pathway, promoting PD-L1 expression in TANs.^70^ In glucose-deprived TME, TANs show higher GLUT1 expression, glucose uptake, and glycolysis.^95^ Enhanced glycolysis promotes the formation of neutrophil extracellular traps (NETs) which are decondensed chromatin combined with content from their azurophilic granules extruded by TANs.^96^ NETs can bolster tumor growth. They can directly isolate cancer cells from cytotoxic cells, shielding the former from destruction.^97–99^ In pancreatic cancer models, NETs have been found to promote tumor growth indirectly by activating stellate cells and promoting desmoplastic stromal cell activation.^100^ NETs also enhance tumor cell motility, aiding metastasis, by binding with the NET-associated DNA receptor CCDC25 on tumor cell surfaces, operating through the ILK-β-Parvin pathway.^101^ Established tumor cells have the ability to recruit neutrophils by secreting factors such as IL-8.^102^ Furthermore, cancer cell-derived factors, such as G-CSF, can induce neutrophils to release NETs.^103^ This process potentially creates a positive feedback loop that may play a significant role in promoting the development of metastatic tumors.

TANs compensate for glucose deficiency by oxidizing fatty acids, supplying sufficient NADPH to promote the production of ROS, which can inhibit T cells.^104,105^ Besides fatty acids, TANs can also utilize the metabolic product of glutamine, alpha-ketoglutarate, to participate in the tricarboxylic acid cycle, maintaining energy metabolism and NADPH production.^106,107^

Despite the protumor inclinations of TANs, some studies indicate that effective interventions can steer TANs toward an anti-tumor polarization. For example, blocking these inducing factors, such as TGF-β or glutaminase, has been found to reverse the suppressive state of TANs.^105,106^ Besides, type I IFNs can promote N1 polarization in TANs, inhibiting tumor metastasis.^108,109^ Recent research has revealed that combining tumor necrosis factor (TNF), a CD40 agonist, and a tumor-binding antibody can activate neutrophils to kill tumor cells through a complement-mediated oxidative mechanism. This therapy strategy employs TNF to promote the recruitment of neutrophils to tumors, coupled with the use of CD40 agonists to activate the tumor-killing functions of neutrophils. Subsequently, anti-tumor antibodies are utilized to stimulate the ADCC of neutrophils. This combined treatment approach also triggers the activation of the complement system, ultimately leading to tumor destruction.^110^ This multi-targeted combination method offers a novel perspective for reactivating the immune system. It involves intervening at various stages of immune cell-mediated tumor elimination, including recruitment, activation, and killing, enabling immune cells to overcome cancer treatment barriers through a predefined intervention pathway. However, an area for exploration is how to select interventions for different targets to achieve optimal synergy. The possibility that this synergistic approach may activate new pathways influencing tumor prognosis and the mechanisms of such synergy represents a direction for future research.

### Natural killer cells

Natural Killer (NK) cells, belonging to the innate lymphoid cells, can kill tumor cells through various mechanisms independent of tumor antigens, such as releasing perforin and granzyme.^111^ However, NK cell function is suppressed by the TME. The hypoxic conditions of the TME induce functional impairments in NK cell effectors, such as downregulation of NKG2D, NKp30, and CD16, upregulation of immunosuppressive molecules like TIGIT, PD-L1, PD1, and TIM3, and promotion of autophagy-induced degradation of granzyme B.^112,113^ The hypoxia also leads to the accumulation of adenosine in the TME, which inhibits the anti-tumor function of NK cells through their adenosine A_2A_ receptor.^114^

A metabolic rearrangement occurs in NK cells within the TME, including impairments in energy metabolism such as downregulation of glucose transporter GLUT1, mitochondrial damage, and reduced glycolysis and oxidative phosphorylation.^112,115^ TGF-β is a key driver of energy metabolic impairment, with blockade of its activity reversing many energy metabolic and functional deficiencies.^116^ In addition to energy metabolism, other metabolisms are also abnormal. It has been found that lung stromal cells can transport lipids to NK cells via exosome-like vesicles, causing lipid accumulation in NK cells.^117^ In lipid-rich environments, accumulated lipids induce NK cells to inhibit the mTORC1 pathway through upregulation of the PPAR pathway.^118^ The inhibition of mTOR, which is thought to be the central metabolic regulator promoting glycolysis, reduces the glycolytic capacity of NK cells.^118,119^ Tumor cells also transmit inhibitory signals through direct cell contact. For instance, the 4-1BB/4-1BBL interaction upregulates the CD73 expression in NK cells. Then CD73^+^ NK cells acquire a regulatory phenotype, promoting IL-10 and TGF-β production through upregulated STAT3 activity.^120^

Despite the extensive suppression in the TME, the anti-tumor phenotype of NK cells can still be rescued. IL-15 is one of the promising molecules in this regard. IL-15 activates mTORC1 through IL-15-PI3K-AKT pathway, reversing glycolysis inhibition.^112,121^ Additionally, IL-12, IL-18, type I IFNs and Nrf2 activators have also been found to enhance the anti-tumor activity of NK cells in the TME.^115,122,123^

### T cells

T cells are a critical part of the immune response against cancer.^124^ They mature in the thymus and can be divided into different types based on their various functions and surface molecule markers. Among them, central T cell populations in tumors are helper CD4^+^, regulatory CD4^+^, and cytotoxic CD8^+^ cells.^125–128^

#### CD8^+^ cytotoxic T lymphocytes

CD8+ cytotoxic T lymphocytes (CTLs) are the primary tumor-killing cells, which can activate themselves through T cell receptor binding to antigens presented by MHC-I molecules, expressing high levels of IFNγ, TNFα, perforin, and granzymes to kill tumor cells.^129^

Elevated levels of T-cell infiltration have been identified to correlate with improved prognostic outcomes in tumor patients, as well as enhanced responsiveness to ICB.^130^ However, as tumors progress, CTLs gradually display functional impairment, losing their tumor-suppressing capabilities.^124^ The TME primarily induces CTL dysfunction through five mechanisms: senescence, tolerance, anergy, exhaustion, and ignorance.^124,131^

Metabolic defects or rearrangements in CTLs are involved in these mechanisms. Tumor cells and their supporting cells compete for glucose, leading to a relative deficiency in glucose uptake by CTLs.^132^ CTLs also downregulate the activity of enolase 1, a critical enzyme in the glycolytic pathway, impairing glycolysis and thus diminishing their anti-tumor functionality.^133^ Additionally, the hypoxic environment of tumors induces the accumulation of lactate. As a byproduct of glycolysis, lactate inhibits the PI3K/Akt/mTOR pathway, further suppressing glycolysis in CTLs.^134^ In vitro experiments have shown that lactate can inhibit the proliferation and cytokine production of human CTLs.^135^ Accumulation of lactate and the acidic environment of the TME are also found to reduce CTL infiltration.^136^ The knockdown of lactate dehydrogenase A (LDHA) in tumor cells, which reduces lactate production, has been successful in reversing the decrease in CTL infiltration. Many solid tumors also accumulate lipids, including cholesterol. The accumulation of cholesterol has been found to upregulate T-cell expression of PD-1, 2B4, TIM-3, and LAG-3, expressing high levels of immune checkpoints and inducing CTL exhaustion.^137^ Other small molecules also contribute to promoting CTL immune suppression, for example, adenosine binding with the A_2A_ adenosine receptor on CD8 + T cells affects their cytokine production activity and upregulates inhibitory checkpoint molecules such as PD1.^138^ Canine urine acid can also promote PD1 expression by activating the CTL’s AHR.^139^ Interestingly, cell necrosis-induced release of potassium ions in the TME can weaken CTL nutrient uptake, simultaneously inducing a starvation response leading to autophagy. Autophagy results in the consumption of Acetyl Coenzyme A (AcCoA) in the nucleus, thus reducing histone acetylation and limiting the expression of effector genes.^140^

Intervening in these inhibitory factors can reverse the anti-tumor function of CTLs. Strategies targeting the insufficient energy metabolism of CTLs have been found to promote their anti-tumor function. For example, using alternatives to glucose, such as inosine, which can be converted into phosphorylated ribose and enter the TCA cycle, combined with immunotherapy, has been shown to reduce tumor burden and increase survival rates.^141^ Using agonists of peroxisome proliferator-activated receptors (PPARs) to enhance fatty acid breakdown metabolism which compensates the inadequate glucose metabolism contributes to increasing the anti-tumor capacity of CTLs.^142^

Notably, early-stage tumor-induced CTL dysfunction is reversible. In contrast, late-stage dysfunction is irreversible, and aside from expressing PD-1 and LAG3, these cells express CD38, CD39, CD101, and TIM3 and remain unresponsive to ICB and vaccine treatments.^124,143^ T-cells exhibiting a late-stage exhaustion phenotype may still potentially suppress tumor growth, but they exist in a “stagnant” state.^144,145^

#### Helper CD4^+^ cells

Under environmental factors and self-secreted cytokines, CD4^+^ T cells can differentiate into functional T helper (Th) cell subsets, primarily including Th1, Th2, and Th17.^146^ Each subtype plays a distinct role in the progression of tumors.

Th1 cells differentiate from CD4^+^ T cells under the induction of IL-12 and IFN-γ.^147,148^ These cells secrete anti-tumoral cytokines such as IL-2, TNF-α, and IFN-γ.^149^ IL-2 originating from Th1 cells promotes the proliferation and activation of NK cells as well as differentiation and expansion sustaining of CTLs.^150–152^ Moreover, Th1 cells are essential for the cross-presentation by DCs to prime CTLs.^153,154^ Th2 differentiation is dependent on IL-4,^155^ and Th2 cells primarily produce cytokines, including IL-4, IL-5, IL-10, IL-13, and IL-17. In contrast to Th1, Th2 mainly promotes tumor progression. A reduced Th1/Th2 ratio is associated with poor patient prognosis.^156,157^ The differentiation of Th17 is mainly triggered by IL-1β, IL-6, IL-21, IL-23 and TGF-β.^154,158,159^ Th17 cells primarily secrete IL-17, IL-21, IL-22, IL-10, IL-23, and CCL20, with their roles remaining a subject of debate in cancer.^149,160^

The functionality of different subgroups of Th cells varies, thereby impacting the prognosis of tumors based on their subgroup composition. However, it’s noteworthy that this composition ratio is also modifiable. VEGF, IL-4, IL-10, and TGF-β have been shown to favor Th2 differentiation, thus elevating the Th1/Th2 ratio.^150,153,161^

#### Regulatory CD4^+^ cells

Regulatory T cells (Tregs) represent a subset of T cells, distinguished by their immunosuppressive functions. Tregs express high-affinity trimeric IL-2 receptor complexes and thus outcompete CTLs for essential activation factors, thereby starving CTLs of IL-2 signals.^162,163^ Moreover, Tregs secrete various immunosuppressive cytokines, including TGF-β, IL-10, and IL-35.^164,165^ Elevated levels of TGF-β can induce CD4^+^ T cells to differentiate into Tregs, thus forming a positive feedback loop that amplifies the Treg population.^146^ Research has shown that Tregs heavily infiltrate human tumors, accounting for 10-50% of CD4^+^ T cells within the tumor, compared to only 2–5% in the peripheral blood of cancer-free individuals.^166^

The TME is specifically tailored to be high in lipids, low in glucose, and hypoxic, favoring the conditions for Tregs. Unlike M1 macrophages and CTLs, which primarily rely on glycolysis for energy, Tregs predominantly depend on oxidative phosphorylation (OXPHOS) and fatty acid oxidation (FAO) for their metabolic needs, with lipid oxidation being the major metabolic source for Treg production.^167^ Inhibiting the key enzyme in fatty acid metabolism, carnitine palmitoyltransferase-1 (CPT1), hampers the proliferation of Tregs.^168^ The lipid-rich environment of the TME facilitates this process. Environments high in glucose impair Treg cell function and stability and enhance the therapeutic activity of CTLA-4 inhibitors.^169,170^ Interestingly, Treg could adapt to a high glucose environment by upregulating the production of glycolytic by-product lactate, and high lactate treatment enhanced Treg tolerance to a high glucose environment.^169^ Moreover, hypoxia helps maintain the function of Treg. Supplemental oxygenation reduces the number of Tregs and the levels of the inhibitory cytokine TGF-β.^171^

Interestingly, Tregs in the TME retains a degree of pro-inflammatory plasticity. IL-12 and IL-6 may be critical factors in encouraging the reprogramming of Tregs to acquire Th1 and Th17 functionalities, respectively.^172^ In murine models of lung cancer, the presence of Th1-like Tregs has been observed, characterized by the co-expression of T-bet (a Th1 marker) and FOXP3. These Tregs regain partial Th1 functionality and express low IL-12, IFN-γ, and TNF-α.^173^

In summary, immune cells within the TME, as illustrated in Fig. 1, exhibit two pivotal characteristics. They show a tendency for both pro-tumoral and anti-tumoral differentiation and polarization, generally leaning towards a pro-tumoral phenotype educated by the TME with the characteristics of hypoxic, lack of sugar, fat-rich, low PH, high lactic acid, high potassium, and high adenosine. Additionally, these cells possess a significant degree of plasticity, allowing for their reprogramming through targeted interventions. The convergence of these features suggests that the targeted reshaping of the immune microenvironment, rather than solely focusing on the elimination of a specific cellular component, holds substantial potential for advancing cancer therapy.

## Innate immune pathways in cancer

In the initial section, we elucidated the landscape of immune suppression within the TME and discussed the anti-tumor potential of various immune components. Existing research provides valuable insights for intervening in this microenvironment to reactivate immune functions, suggesting the feasibility of reprogramming both the immune landscape and immune cells. Considering the diverse constituents of the TME and the complexity of immune suppression mechanisms, therapies targeting specific immune-suppressive molecules such as ICB or direct application of a certain cytokine-like IFNs, are often insufficiently robust and are susceptible to tumor immune evasion.^174,175^ Consequently, focusing on targets that are ubiquitously expressed and have multiple downstream immune-activating effects becomes imperative.

Innate immune pathways, pervasive across different cell types, offer significant potential for enhancing the tumor immune microenvironment.^5^ Their activation releases a plethora of IFNs and pro-inflammatory cytokines, triggering various pathways. The widespread presence in the TME of molecules that stimulate innate immune signaling pathways, such as DAMPs, underscores the durability and broad applicability of targeting innate immune pathways. However, the aberrant intracellular signal transduction networks among immune cells and the adaptive changes in tumor cells within the microenvironment have incapacitated innate immune signaling pathways from performing their regular functions. Therefore, deciphering and modulating these innate immune pathways within the TME is crucial for harnessing them to improve cancer therapy.

**Fig. 2:**
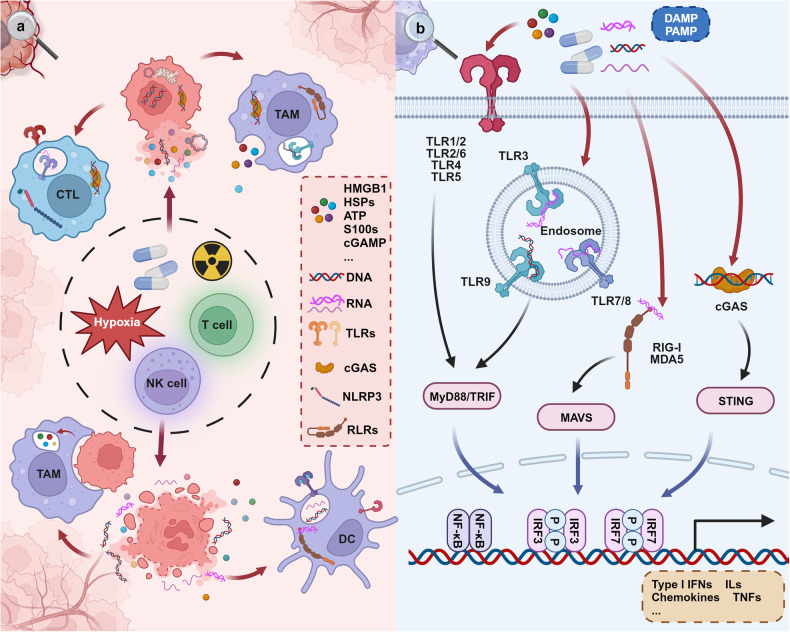
Innate immune pathways in the TME. **a** Sources of initiating factors for the activation of innate immune pathways. Under environmental stressors such as drugs, immune cytotoxic cells, and hypoxia, tumor cells undergo leakage or cell death, leading to the release of DAMPs into the TME. These DAMPs are sensed by PRRs in various cell types within the TME, thereby activating innate immune pathways. In this process, APCs can also directly engulf tumor cells, promoting the generation of DAMPs. **b** Signaling Pathways of Innate Immunity. Innate immune pathways are ubiquitously present in various cell types and are activated by components in the TME. This is primarily driven through three key steps: receptor sensing, signal transduction through adapter molecules, and the initiation of immune-related molecular transcription by transcription factors, thereby modulating the TME. Created with BioRender.com

Therefore, in this section, we delve into the functions of commonly encountered innate immune pathways specifically those pattern recognition receptor (PRR) related pathways sensing DAMPs and PAMPs,^176,177^ at both cellular and molecular levels (Fig. 2.), along with their roles in the progression of tumors. We also attempt to summarize the aberrant regulatory mechanisms that account for the dysfunctional activity of these innate immune pathways within the TME.

Our discussion traverses from how intracellular signaling networks construct the basic functions of innate immune pathways, to how downstream effector molecules of these pathways influence the onset, progression, and metastasis of tumors. This discourse provides insights into the mechanisms of action of innate immune pathways and potential intervention targets, as well as how interventions in these same pathways can lead to heterogeneous downstream outcomes. The innate immune pathway network is not uniformly distributed among various cell types. Instead, it is modulated through cell type-specific pathways, endowing these functional units with distinct downstream outputs in response to the same innate immune pathway stimuli. This cell-specific modulation forms the basis for a spectrum of outcomes that can either support or inhibit tumor growth.

### cGAS-STING pathways

The cGAS-STING pathway can sense double-stranded DNA (dsDNA) in the cytoplasm, playing a pivotal role in innate immunity.^178^ This pathway involves the second messenger cyclic GMP–AMP synthase (cGAS) and the cyclic GMP–AMP receptor stimulator of IFN genes (STING).^179^ Activation of the cGAS-STING pathway leads to the production of Type I IFNs and various cytokines, holding substantial potential for enhancing antitumor immunity.

#### Signaling function of cGAS-STING

##### Signaling of cGAS-STING

**Fig. 3:**
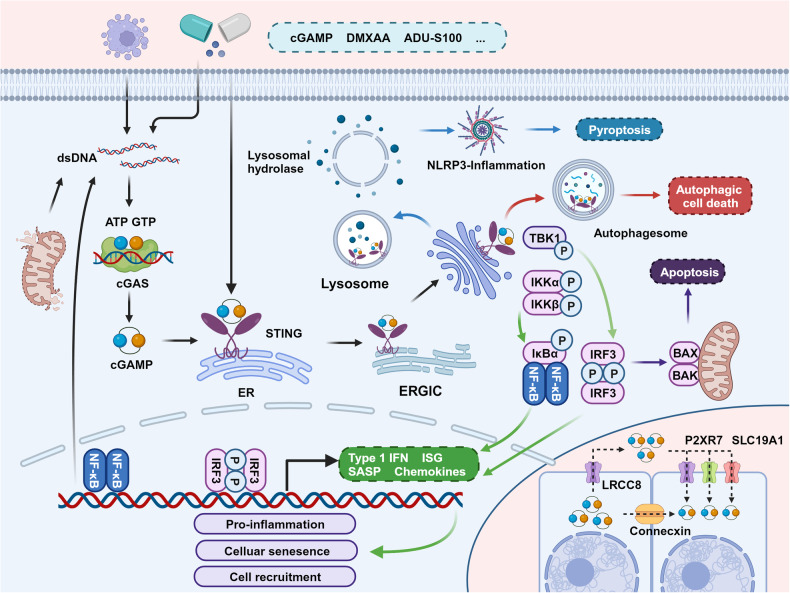
Signaling function of the cGAS-STING pathway. Upon activation, STING primarily exerts its effects through four major pathways. (1) inducing senescence or prompting surrounding cells to eliminate aberrant cells by releasing type I IFN and cytokines; (2) activating pyroptosis via NLRP3; (3) inducing cell death through autophagy; and (4) activating apoptosis. Furthermore, cGAMP can also propagate within the TME, mediating the activation of STING in surrounding cells. Created with BioRender.com

cGAS can detect DNA and initiate a signaling cascade through the cGAS-cGAMP-STING-TBK1-IRF3 axis, ultimately triggering the transcription of type I IFNs^178^ (Fig. 3). In addition, STING can activate the MAPK and NF-κB pathways, although the mechanisms behind this activation remain to be clarified.^180^

Notably, besides type I IFNs, the activation of these pathways also promotes the expression of several molecules, including IFN-stimulated genes (ISGs), pro-inflammatory cytokines, and chemokines. These molecules could induce cellular senescence.^181,182^ This irreversible state of cell cycle arrest gives rise to the senescence-associated secretory phenotype (SASP).^183^ Typical components of SASP include cytokines (such as IL-6, IL-8, IL-1β, and GM-CSF), chemokines (such as MCP-1 to 4, MIP-1α, MIP-3α), proteases (such as MMP 1, 3, 9, and 12), and growth factors (such as TGF-β).^183^ These components can promote inflammation and reinforce cell cycle arrest to eliminate these cells.^184–187^ A deficiency in SASP response has been observed to accelerate tumor growth.^184,186^

##### Induction of autophagy

The cGAS-STING pathway also participates in the process of cellular autophagy. Golgi membranes containing STING can serve as a membrane source for autophagosome biogenesis, and cGAMP stimulation promotes lipidation of the key autophagy biogenesis factor, LC3.^180^ Notably, the STING-autophagy pathway can drive an autophagic cell death program during the cellular replicative crisis, serving as a preventative mechanism against the formation of tumor cells (Fig. 3).^188^

##### Lysosome-mediated cell death

STING can also induce lysosome-mediated cell death, a lytic cell death program. STING can translocate to lysosomes and accumulate, which leads to lysosomal membrane permeabilization. This process subsequently releases lysosomal hydrolases, resulting in cell death.^189^ The damage to the plasma membrane integrity and cell potassium efflux activated by this process triggers the NLRP3 inflammasome, which in turn induces the release of inflammatory factors and cell death (Fig. 3).^189^

##### Promotion of apoptosis

Phosphorylated STING can interact with the pro-apoptotic proteins BAX and BAK, located on the mitochondrial membrane, thus promoting cell apoptosis.^190,191^ In Addition to this pathway, the activation of STING, through the synergistic action of IRF-3 and p53, leads to the upregulation of *Noxa* and *Puma*, thereby promoting cell apoptosis (Fig. 3).^192^

##### Signal transmission

Once activated, cGAS generates cGAMP, which can be propagated to surrounding cells through gap junctions.^193^ Additionally, cGAMP can be released into the extracellular fluid via mechanisms such as cell death, and be taken up by surrounding cells through endocytosis or transport proteins like SLC19a1 and P2XR7, thereby exerting effects within these cells (Fig. 3).^194,195^ For instance, cGAMP released by tumor cells can be taken up by immune cells, leading to their self-activation.^195^ Interestingly, recent studies have shown that activated STING can also be transferred between cells. This process is mediated by RAB22A-mediated non-canonical autophagy and can promote anti-tumor immunity, adding a new dimension to our understanding of cGAS-STING signaling in the TME.^196^

These functions of the cGAS-STING pathway are crucial mechanisms for inducing tumor cell death and also form the basis for our understanding of the role the cGAS-STING pathway plays in tumors.

#### Role of cGAS-STING pathway in tumor

##### cGAS-STING pathway and tumorigenesis

The cGAS-STING pathway can suppress tumorigenesis through the maintenance of cellular homeostasis. As previously mentioned, the cGAS-STING pathway is involved in the autophagic process of cells. Autophagy aids in the clearance of misfolded proteins or damaged organelles, thereby preserving cellular homeostasis.^197^ Disruption of autophagy has been found to promote tumorigenesis.^198^ Studies have revealed that STING-driven autophagy can prevent the proliferation of cells undergoing replicative stress.^188^

An additional way in which cGAS-STING prevents normal cells from transforming into cancerous cells is by controlling the replication of the genome. The cGAS-STING pathway can induce the SASP in cells that produce cytoplasmic DNA under stress conditions, thereby promoting senescence and subsequent clearance of these aberrant cells. Oncogenic RAS has been shown to induce senescence and SASP in a cGAS-STING-dependent manner. Loss of cGAS or STING results in impaired senescence and SASP responses, leading to accelerated spontaneous immortalization and increased tumor growth.^182,184–186^ Interestingly, cGAS can also intervene in cell replication in a STING-independent manner, acting as a “brake” to suppress genomic instability.^199^ Contradictorily, nuclear-localized cGAS has been shown to inhibit homologous recombination repair (HRR) of damaged DNA, thereby promoting tumorigenesis.^200^ However, these two findings offer different implications for cGAS at different stages of tumor development. During tumorigenesis, the genome-stabilizing function of cGAS essentially only slows down the accumulation of DNA-damaged cells, making its role in inhibiting DNA repair dominant. Once the tumor is formed, the error of the genome already exists. The inhibition of cGAS can accelerate tumor cell replication, thereby increasing tumor sensitivity to radiation and chemotherapy.^199^ This implies that activating the nuclear cGAS pathway during radiotherapy and chemotherapy may lead to treatment resistance. The varied functions of cGAS, depending on its cellular location and the cell’s state, suggest that singular interventions targeting cGAS could yield unpredictable outcomes.

Surprisingly, in inflammation-induced colorectal cancer, the cGAS-STING pathway can also inhibit inflammation, thereby limiting tumorigenesis. The cGAS-STING pathway can restrict the activation of NF-κB and STAT3 signaling pathways, thereby downregulating the level of inflammatory cytokines, including IL-6 and keratinocyte-derived chemokine. The observation that mice with a STING^gt/gt^ (point-mutated STING) genotype exhibit reduced levels of caspase-1 activation and IL-18 release in their colonic tissue suggests that STING may mediate the protective effect on the intestinal barrier through crosstalk with caspase-1, facilitating the release of IL-18.^201^ An intriguing question arises as to why STING does not trigger a pro-inflammatory response in this context. This atypical effect could be related to the inflammation-induced mouse cancer model used in the study. Since the colorectal cancer inflammation-induced model simulates long-term chronic inflammation in the gut, it may induce a transformation in the STING pathway within intestinal epithelial cells. The STING pathway is known for its plasticity, and repeated stimulation of STING can lead to the suppression of the IFN pathway.^12^ Indeed, no significant difference in IFN-β expression levels was observed between STING^gt/gt^ and wild-type mice in this model.^201^

Furthermore, the exploration of STING’s tumorigenic effects in the study was conducted in the absence of STING agonists. The application of STING agonists, as acute and potent stimulators of the pathway, could potentially lead to a predominant downstream IFN release and exacerbation of inflammation. Investigating how STING agonists might influence the pathway’s response, especially in terms of inflammation and tumor development, represents a valuable perspective for future research.

##### cGAS-STING pathway and tumor progression

After the onset of tumors, the cGAS-STING pathway plays a crucial role in tumor clearance. As mentioned before, activation of the cGAS-STING pathway can mediate the activation of immune cells by secreting type I IFNs and pro-inflammatory cytokines. This includes 1) promoting the maturation of dendritic cells,^202^ enhancing the expression of MHC-I, MHC-II, and co-stimulatory molecules^203,204^; 2) facilitating T-cell recruitment,^205^ priming, and effector function maintenance^206^; 3) bolstering Th1 responses^207^; reducing intra-tumoral Treg numbers^208^; 4) and activating NK cells for enhanced tumor-killing capabilities.^209^ 5) Moreover, the cGAS-STING pathway can directly induce tumor cell apoptosis and contribute to remodeling tumor vasculature within the TME.^209–211^ 6) Activation of cGAS-STING can also induce immunological memory, limiting tumor recurrence.^212^ Numerous preclinical models have demonstrated the beneficial impact of activating the cGAS-STING pathway on tumor prognosis (Table 1).

**Table 1:** Representative evidence about innate immune pathway effect on tumor

Pathways	Tumor categories	Model	Key involving cell types	Effect on tumor	Main mechanism	Refs.
cGAS-STING	Colorectal cancer	Xenograft murine model of MC38	TAMs		Activation of the STING-IRG1 axis in TAMs by STING agonists promotes their reprogramming into an anti-tumor phenotype	^659^
cGAS-STING	Glioma	Xenograft murine model of GL261/CT2A	Endothelial cells pericytes NK cells	Anti	STING agonists can activate GBM, particularly within the tumor vasculature. NK cells mediate the tumor elimination driven by STING agonist stimulation.	^212^
cGAS-STING	HCC	Spontaneous Murine model induced by the hepatocyte-specific mutagen diethylnitrosamine	Kupffer cells	Anti	cGAS-STING pathways promote the release of TNF-α and type I IFN by Kupffer cells, which activate the T cell and induce cell death in tumor cell	^660^
cGAS-STING	HNSCC	Xenograft murine model of SCC7	CTL	Anti	Application of STING agonists at the resection sites in HNSCC induces type I IFN production by host cells (not tumor cells), thereby facilitating CTL-mediated tumor clearance and inhibiting recurrence.	^661^
cGAS-STING	Non-muscle invasive bladder cancer	Spontaneous Murine model induced by N-methyl-N-nitrosourea	Macrophage	Anti	cGAS-STING activation promotes the pro-inflammatory polarization of macrophages	^662^
cGAS-STING	Fibrosarcoma; Melanoma	Xenograft murine model of MCA205/B16F10	DC; CTL	Anti	DAPK3 enhances the activation of the STING pathway through post-translational modifications. cGAS-STING activation produces IFN-β, which facilitates the infiltration of CTLs and DCs	^266^
cGAS-STING	Lymphoma; Melanoma;	Xenograft murine model of RMA-S/B16-BL6	NK cells	Anti	tumor-derived cGAMP activates the STING pathway in immune cells within the TME, which leads to the secretion of IFN that, in turn, bolsters NK cell-mediated anti-tumor immunity.	^123^
cGAS-STING	NSCLC	Xenograft murine model of LLC	TAMs	Anti	mtDNA activates the cGAS-STING pathway, promoting the reprogramming of TAMs towards an anti-tumoral phenotype.	^663^
cGAS-STING	Metastatic bone cancer	Xenograft murine model of LLC	Neuron; Osteoclast; CTL	Anti	STING-mediated IFN-I signaling exerts its effects through three key mechanisms: 1) Directly inhibiting nociceptor excitability to alleviate bone pain; 2) Directly suppressing osteoclast differentiation to reduce bone degradation; 3) Facilitating the migration of CD8^+^ T cells into the bone marrow TME, thereby enhancing anti-tumor immunity and reducing tumor burden.	^664^
cGAS-STING	Melanoma;	Xenograft murine model of B16F10	Endothelial cells	Anti	Endothelial cells, in response to STING agonists, secrete type I IFN, thereby activating CTLs.	^665^
cGAS-STING	Melanoma;	Xenograft murine model of B16F10	TAMs；Stromal cells	Anti	STING agonists promote activated monocytes (CD11b^+^ Ly6C^+^ MHC-II^+^) and generate TNFα, mediating anti-tumor effects; the activation of STING in stromal cells indirectly leads to the activation of APCs through the production of inflammatory cytokines.	^666^
cGAS-STING	Melanoma;	Xenograft murine model of B16F10	DC; CTL	Anti	STING agonists induce increased infiltration of DCs and CTLs. Concurrently, DCs acquire a phenotype capable of promoting vascular normalization and the formation of tertiary lymphoid structures.	^667^
cGAS-STING	BRCA-mutant breast cancer	Spontaneous murine model driven by concurrent ablation of Brca1 and Trp53 via Cre-loxp system	TAMs	Anti	STING agonists reprogram M2-like pro-tumoral macrophages to an M1-like anti-tumoral state in a macrophage STING-dependent manner.	^600^
cGAS-STING	bladder cancer	Xenograft murine model of Primary BC cell lines (SYBC1)	Tumor cells; fibroblasts	Pro	The activation of the cGAS-STING pathway in tumor cells leads to the release of Type I IFN, which induces the formation of a fibroblast subpopulation expressing the urea transporter SLC14A1. This subpopulation subsequently enhances tumor stemness through the activation of the Type I IFN/IFNAR-STAT1/WNT5A/β-catenin pathway, thereby promoting the stemness of breast cancer tumor cells.	^222^
cGAS-STING	Metastatic brain cancer	Xenograft murine model of MDA231-BrM2/ H2030-BrM3	Astrocytes; Tumor cell	Pro	Metastatic tumor cells form gap junctions with astrocytes to transfer cGAMP, thereby activating the STING pathway in the latter, which subsequently leads to the release of IFN and TNFα. These cytokines, in turn, activate the STAT1 and NF-κB signaling pathways in tumor cells, promoting both tumor growth and chemoresistance.	^225^
cGAS-STING	TNBC	Xenograft murine model of MDA-MB-231/4T1	Tumor cell	Pro	Tumor cells with CIN activate the cGAS-STING-non-canonical NF-κB pathway to produce IL-6, which in turn activates the STAT3 signaling pathway to promote cell survival.	^219^
cGAS-STING	TNBC; NSCLC	Xenograft murine model of MDA-MB-213/4T1/ H2030	Tumor cell	Pro	Tumor cells with CIN activate the cGAS-STING-non-canonical NF-κB pathway to promote metastasis in a tumor cell-autonomous fashion	^13^
TLR1	NSCLC	Xenograft murine model of A549	Tumor cells	Pro	TLR1 mediates the expression of anti-apoptotic proteins BCL-2 and Survivin and cell cycle related protein cyclin D1 through the NF-κB signaling pathway.	^335^
TLR1/2	Leukemia	Xenograft murine model of FBL-3	DCs; Macrophages	Anti	Activation of TLR1/2 on DCs enhances antigen presentation and amplifies T-cell activation, while the stimulation of TLR1/2 on macrophages promotes the activation of NK cells through the secretion of cytokines. Activated CTLs and NK cells collectively mediate tumor killing.	^314^
TLR2	Glioma	Xenograft murine model of GL261	Microglia; CTL	Anti	TLR2 in microglia promotes the proliferation and activation of CD8^+^ T cells through the TLR2-MHC I axis	^648^
TLR2	HCC	Spontaneous Murine model induced by DEN	MDSC;	Anti	TLR2 inhibits the production of IL-18, thereby reducing the infiltration of MDSCs	^322^
TLR2	Breast cancer	Spontaneous Murine model driven by HER2	CSC; Treg	Pro	TLR2 promotes the self-renewal of CSCs and facilitates the proliferation of Tregs.	^668^
TLR2/6	NSCLC	Xenograft murine model of LLC	Macrophages	Pro	Upregulation of proteoglycan versican expression in tumors activates TLR2/TLR6 in myeloid cells, subsequently promoting their secretion of TNF-α and versican, which markedly enhances metastatic tumor growth.	^669^
TLR6	Colorectal Cancer	Spontaneous Murine model induced by azoxymethane	*Lactobacillus*; Tumor cells	Anti	The absence of TLR6 leads to the loss of *Lactobacillus* and induces anti-apoptotic mechanisms in tumor cells, thereby promoting tumor initiation and progression.	^311^
TLR3 and TLR9	Glioma	Xenograft murine model of GL261	Microglia; Macrophages	Anti	Co-activation of TLR3 and TLR9 amplifies the release of pro-inflammatory cytokines, motility, and phagocytic activity in microglia and macrophages.	^670^
TLR3	Lymphoma	Xenograft murine model of EG7	NK cells; CTL	Anti	TLR3 agonists induce significant activation of NK cells and CTLs.	^318^
TLR3 and MDA5	Colon cancer	Xenograft murine model of CT26	Tumor cells	Anti	PolyI:C mediates tumor cell death through TLR3/RIP3-Dependent Necroptosis.	^327^
TLR3	Breast cancer	Xenograft murine model of SUM149 or SUM190	CSCs	Pro	β-Catenin and NF-κB co-activation triggered by TLR3 stimulation facilitates stem cell-like phenotypes	^336^
TLR4	Glioma	Xenograft murine model of RG2	CSCs	Anti	TLR4 activation promotes higher levels of MHC molecules, CXCL10, and TNF-α in CSCs, and recruits an increased number of CD8^+^ T cells.	^671^
TLR4	Breast cancer; melanoma	Xenograft murine model of 4T1 or B16	Macrophages	Anti	Paclitaxel reprograms M2-polarized macrophages toward an M1-like phenotype in a TLR4-dependent manner.	^320^
TLR4	Pancreatic cancer	Xenograft murine model of 4T1 or B16	NK cell	Anti	Agonists of TLR4 enhance the tumoricidal activity of NK cells via the MAPKs/NF-κB signaling pathway.	^319^
TLR4	HCC	Spontaneous murine model induced by combination of DEN, CCl_4_ and 10% alcohol drinking	Tumor cells	Pro	TLR4 signaling promotes self-proliferation of HCC cells through a COX-2/PGE2/STAT3 positive feedback loop.	^672^
TLR5	Colon cancer	Xenograft murine model of DLD-1	Neutrophils	Anti	Activation of TLR5 promotes tumor necrosis and regression by facilitating neutrophil infiltration.	^673^
TLR5	Breast cancer	Xenograft murine model of MDA-MB-468 or MCF-7	Tumor cells; Neutrophils	Anti	Activation of TLR5 inhibits cellular proliferation of tumor by inducing tumor cells to secrete soluble factors, while simultaneously increasing neutrophil infiltration.	^674^
TLR5	Sarcoma	Spontaneous murine model driven by combination of Trp53^flox/flox^; LSL-Kras^G12D/+^ and cre system	MDSCs; γδT cells	Pro	TLR5 signaling drives systemic upregulation of IL-6, which in turn promotes the mobilization of MDSCs and induces the generation of immunosuppressive galectin-1-expressing γδT cells, consequently accelerating tumor growth.	^675^
TLR7	PDAC	Xenograft murine model of KPC	CTL; Treg	Anti	The TLR7 agonist enhances the infiltration and activity of CD8^+^ T cells in the TME, while concurrently reducing the frequency of Treg.	^323^
TLR7	Colon cancer	Xenograft murine model of CT26	MDSCs	Anti	The TLR7 agonist diminishes the quantity of MDSCs both within the tumor and in circulation, while inducing their maturation and driving them to acquire an antigen-presenting phenotype.	^324^
TLR7/8	Colon cancer; melanoma	Xenograft murine model of MC38 or B16F10	Macrophages	Anti	The TLR7/8 agonists drive the anti-tumoral polarization of TAMs.	^321^
TLR7/8	NSCLC	Xenograft murine model of ASB-XIV	DCs; T cells; Macrophages	Anti	The activation of TLR7/8 agonists stimulates dendritic cells, promotes T-cell proliferation, and converts pro-tumoral immune cells into anti-tumoral phenotypes.	^676^
TLR7/8	Bladder cancer; melanoma; renal adenocarcinoma	Xenograft murine model of MB19 or B16F10 or Renca	DCs; CTL	Anti	Activation of TLR7/8 agonists triggers DCs activation and proliferation, which subsequently results in the expansion of antigen-specific CD8^+^ T cells and an enhanced CTL response.	^316^
TLR7/8	NSCLC	Xenograft murine model of A549 or B16F10 or Renca	Tumor cells	Pro	Activation of TLR7/8 in tumor cells enhances tumor survival and elevates resistance to chemotherapy, while concurrently promoting the recruitment of myeloid cells to the TME.	^677^
TLR7/8	PDAC	Xenograft murine model of PANC-1	Tumor cells	Pro	Stimulation of TLR7/8 in tumor cells leads to elevated expression of NF-κB and COX-2, thereby enhancing cancer cell proliferation and reducing chemosensitivity.	^678^
TLR9	Glioma	Xenograft murine model of GL261	Tumor cells; Microglia	Anti	Stimulation of TLR9 induces apoptosis in tumor cells and augments the antigen-presenting capability of microglial cells.	^325^
TLR9	NSCLC; Breast Cancer; Colon Cancer	Xenograft murine model of LLC or 4T1 or CT26; spontaneous murine lung cancer model induced by urethane	Tumor cells; CTL	Anti	Activation of TLR9 in tumor cells promotes the infiltration of CTLs through the secretion of CXCL10.	^317^
TLR9	Breast Cancer	Xenograft murine model of MDA-MB-231	Tumor cells	Anti	Activation of TLR9 within tumor cells induces the expression of inflammatory proteins	^679^
TLR9	Melanoma	Xenograft murine model of B16	pDC	Anti	Activation of TLR9 stimulates pDCs to produce type I IFN, thereby enhancing the antigen-presenting capabilities of cDCs	^315^
TLR9	NSCLC	Spontaneous murine model driven by K-ras mutation	mononuclear cells	Pro	Activation of TLR9 in mononuclear cells promotes angiogenesis through the secretion of VEGF.	^304^
TLR9	HCC	Xenograft murine model of Hepa1-6	Tumor cells	Pro	Hypoxia-induced HMGB1 and mtDNA activate TLR9, leading to the activation of the MAPK and NF-κB pathways as well as the expression of the downstream pro-tumorigenic molecules such as IL-6.	^299^
NAIPs	Colorectal cancer	Spontaneous murine model induced by azoxymethane and dextran sodium sulfate	Epithelial cells; Tumor cells	Anti	NAIPs inhibit the tumorigenesis of the colon by promoting apoptosis in epithelial cells induced by carcinogens.	^430^
NOD1	Breast Cancer	Xenograft murine model of MCF-7	Tumor cells	Anti	The NOD1 receptor induces apoptosis in tumor cells and inhibits the proliferative effects induced by estrogen.	^439^
NOD1	Colorectal cancer	Spontaneous murine model induced by azoxymethane and dextran sodium sulfate	T cells	Anti	Intrinsic NOD1 in T cells restricts colitis-associated tumorigenesis by promoting IFNγ production.	^432^
NOD2	Colorectal cancer	Spontaneous murine model induced by azoxymethane and dextran sodium sulfate	Epithelial cells; Tumor cells; Macrophage	Anti	Activation of NOD2 suppresses colorectal tumorigenesis by inducing IRF4, which in turn downregulates the TLR4-MAPK/NF-κB/STAT3 inflammatory pathway.	^428^
NLRC3	Colorectal cancer	Spontaneous murine model induced by azoxymethane and dextran sodium sulfate; spontaneous mouse model of colon cancer driven by the gene encoding adenomatous polyposis coli	Tumor cells	Anti	The NLRC3 protein restricts tumor cell proliferation by inhibiting the PI3K-AKT-mTOR pathway. It also hinders the degradation of tumor suppressors FoxO3a and FoxO1 though inhibiting PI3K-AKT activation. Additionally, NLRC3 curtails tumor cell stemness by suppressing the expression of stemness-associated genes, including BMI1 and OLFM4.	^440,441^
NLRC4	Colorectal cancer	Spontaneous murine model induced by azoxymethane and dextran sodium sulfate;	Epithelial cells; Tumor cells	Anti	The NLRC4 inflammasome mediates the inhibition of tumor cell proliferation and the promotion of apoptosis through caspase-1 signaling.	^431^
NLRC4	Breast cancer	Xenograft murine model of Py8119 or E0771	Macrophages	Pro	In obese conditions, the TME shows increased myeloid cells with activated NLRC4 inflammasomes. This activation leads to IL-1β release, which, in turn, drives angiogenesis and disease progression via adipocyte-mediated VEGFA expression.	^448^
NLRP1	Colorectal cancer	Spontaneous murine model induced by azoxymethane and dextran sodium sulfate;	Epithelial cells	Anti	The NLRP1 inflammasome inhibits the onset of inflammation-induced colon tumorigenesis through the release of IL-1β and IL-18.	^434^
NLRP3	Liver/lung metastases of colorectal cancer	Xenograft murine model of MC38 or CMT93 or LLC	Kupffer cells; NK cells	Anti	Activation of the NLRP3 inflammasome enhances the production of IL-18, which in turn promotes the maturation of hepatic NK cells, augments FasL expression, and amplifies FasL-mediated tumor-killing capabilities.	^459^
NLRP3	Liver metastases of colorectal cancer	Xenograft murine model of MC38	Macrophage	Pro	The activation of the NLRP3 inflammasome in macrophages enhances the secretion of IL-1β, thereby promoting the migratory capacity of tumor cells.	^458^
NLRP3	Colorectal cancer;	Spontaneous murine model induced by azoxymethane and dextran sodium sulfate;	Epithelial cells; Tumor cells; Macrophages	Anti	The activation of the NLRP3 inflammasome induces the production of IL-18, which subsequently promotes the generation of IFNγ, leading to the activation of the tumor suppressor STAT1 signaling pathway.	^435^
NLRP3	Colorectal cancer;	Spontaneous murine model induced by azoxymethane and dextran sodium sulfate;	Macrophages	Pro	Activation of the NLRP3 inflammasome promotes tumor cell proliferation through the release of IL-1β.	^449^
NLRP3	Gastric cancer	Xenograft murine model of BGC-823	Macrophage; epithelial cells; Tumor cells	Pro	The activation of the NLRP3 inflammasome promotes epithelial cell proliferation and gastric cancer tumorigenesis through IL-1β and the enhancement of cyclin-D1 transcription.	^436^
NLRP6	Gastric cancer	Xenograft murine model of MKN45	Tumor cells	Anti	NLRP6 promotes the senescence of gastric cancer cells through the activation of the P14ARF–Mdm2–P53 pathway	^442^
NLRP12	Colorectal cancer;	Spontaneous murine model induced by azoxymethane and dextran sodium sulfate;	Myeloid cells; epithelial cells; Tumor cells	Anti	NLRP12 serves as a negative regulator of non-canonical NFκB and MAPK-mediated inflammation, thereby inhibiting inflammation-induced colorectal cancer.	^429^
RIG-I	HCC	Spontaneous murine model induced by DEN	Tumor cells	Anti	RIG-I amplifies the IFN-JAK-STAT signaling pathway by enhancing the activation of STAT1, leading to the induction of apoptosis in tumor cells.	^488^
RIG-I	Breast cancer	Xenograft murine model of 4T1	Tumor cells	Anti	In breast cancer cells, RIG-I signaling triggers intrinsic apoptosis and pyroptosis, and it further promotes the infiltration of immune cells through the secretion of cytokines.	^501^
RIG-I	AML	hMRP8-PML/RARα-transgenic mice (spontaneous AML model)	Tumor cells	Anti	RIG-I inhibits Src-mediated AKT activation, thereby restraining leukemic stemness and proliferation.	^493^
RIG-I	PDAC	Xenograft murine model of Panc02	Tumor cells; DCs	Anti	RIG-I induces immunogenic cell death in tumor cells, promoting antigen presentation by DCs and the activation of CD8^+^ T cells.	^492^
RIG-I	Lymphoma; colon carcinoma; melanoma;	Xenograft murine model of MC38 or EL4 or B16F10	T cells	Pro	T cell activation-induced upregulation of RIG-I competitively chelates HSP90, inhibiting STAT5 activation, and subsequently decreasing the survival and cytotoxic ability of T cells.	^504^
MDA5	PDAC	Xenograft murine model of Panc02	Tumor cells; CTL	Anti	MDA5 activation induces immunogenic cell death in tumor cells, while simultaneously promoting the activation of tumor-reactive CD8^+^ T cells.	^680^
LGP2	PDAC; colon carcinoma;	Xenograft murine model of Panc02 or MC38	DCs	Anti	In DCs the lack of LGP2 reduced type I IFN production and weakened the priming ability of DCs.	^509^

*EL4* mouse T-cell lymphoma cell line, *Hepa1-6* mouse hepatoma cell line, *Panc02* mouse pancreatic cancer cell line, *MKN45* human gastric cancer cell line, *BGC-823* human gastric cancer cell line, *CMT93* Mouse rectum carcinoma cell line, *MDA-MB-231* human TNBC cell line, *MCF-7* human breast cancer cell line (estrogen-sensitive), *4T1* mouse TNBC cell line, *Py8119* mouse breast cancer cell line, *E0771* mouse breast cancer cell line, *H2030* human lung adenocarcinoma cell line, *B16F10* mouse melanoma cell line, *B16-BL6* mouse melanoma cell line (poorly immunogenic), *CT26* mouse colorectal carcinoma cell line, *MC38* mouse colon adenocarcinoma cells, *MB49* mouse bladder cancer cell line, *Renca* mouse renal adenocarcinoma, *LLC* mouse Lewis lung carcinoma cell line, *MCA205* mouse fibrosarcoma cell line, *RMA-S* mouse lymphoma cell line (Deficient in antigen processing), *SCC7:* mouse squamous cell carcinoma cell line, *A549* human lung cancer cell line, *GL261* mouse glioma cell line, *CT2A* mouse glioma cell line, *KPC* mouse PDAC cell line, *FBL-3* mouse leukemia cell line, *ASB-XIV* mouse lung carcinoma cell line, *EG7* mouse lymphoma cell line containing ovalbumin, *RG2* Rat Glioma Cell Lines, *A549* Human lung cancer cell line, *MDA231-BrM2* Human Breast Adenocarcinoma Cell Line, *MDA-MB-468* Human TNBC cell line, *MCF-7* human breast cancer cell line with estrogen, progesterone and glucocorticoid receptors, *SUM190* Human Breast inflammatory carcinoma Cell Line, *SUM149* Human Breast inflammatory carcinoma Cell Line, *BxPC-3* human pancreatic cancer cell line, *PANC-1* human pancreatic cancer cell line, *SW1990* human pancreatic cancer cell line, *H2030-BrM3* human lung adenocarcinoma cell line, *DLD-1* human colon cancer cells, *TNBC* triple-negative breast cancer, *HNSCC* head and neck squamous cell carcinoma, *HCC* hepatocellular carcinoma, *CSC* cancer stem cells, *NSCLC* non-small-cell lung cancer, *DEN* diethylnitrosamine, *CCL*_*4*_ carbontetrachloride, *PDAC* pancreatic ductal adenocarcinoma, *AML* acute myeloid leukemia

Clinical data also indicate the anti-tumor function of the cGAS-STING pathway. Transcriptional data from subsets of human tumors reveal that reduced expression of cGAS and STING correlates with poorer patient survival rates.^182,213^ High expression of STING is associated with lower pathological grading of glioma.^214^ Additionally, Tumor Treating Fields, a proven effective method for GBM treatment,^215^ has been found to mediate anti-tumor immunity and a cure rate of 42 to 66% through cGAS-STING and AIM-2 (Absent in Melanoma 2, a DNA sensor that activates the inflammasome) dependent pathways.^216^

Interestingly, recent research has revealed that STING is also involved in regulating metabolism. STING targets Hexokinase II (HK2) to inhibit its hexokinase activity, thereby suppressing aerobic glycolysis, reducing lactate production, and inhibiting tumor growth.^217^ STING agonists have also been found to sensitize tumors to immune checkpoint inhibitors.^218^ This discovery suggests a synergistic potential where activating the STING pathway can enhance the effectiveness of treatments that target immune checkpoints.

Tumors with chromosomal instability (CIN) characteristics exhibit suppressed growth when STING is absent. Studies have revealed that in such tumors, the cGAS-STING pathway tends to activate the non-classical NF-κB pathway. The production of IL-6 through this pathway activates the STAT3 pathway in tumor cells, thereby promoting tumor survival and growth. In chromosomal instability (CIN) tumors, the classical downstream type I IFN pathway is not lost but exists in low concentration. Compared to the specific activation of STAT1 by the high concentration of type I IFN, the low concentration of type I IFN simultaneously activates STAT3.^219^ The reasons behind this pathway transformation are unclear, but it may be related to the plasticity changes in the STING pathway due to CIN-induced chronic activation. Such long-term low-level activation may alter signaling networks, leading to immune escape. For instance, the activation of STING can elevate the expression of PD-L1 in tumor cells, which may be through the mediation of infiltrating CD8^+^ IFN-γ^+^ T cells.^220^ Interestingly, recent studies further corroborate the presence of innate immune pathway plasticity, finding that the type I IFN pathway in STING becomes tolerant quickly after initial cGAMP stimulation, with the downstream pathway transitioning to ER stress-related pathways, thus promoting CIN tumor progression.^12^ The functional transformation of the cGAS-STING pathway in specific tumor backgrounds necessitates caution in the clinical application of STING agonists. It is worth exploring why chronic and acute stimulation of STING leads to different pathway effects, the plasticity mechanisms of the cGAS-STING pathway, and whether the pathway is reversible post-chronic stimulation reshaping, potentially restored to produce type I IFN responses upon STING agonist stimulation. Another pivotal factor is the stage of the tumor; in late-stage tumors, immune cells are often in a state of exhaustion, making it difficult to mount an effective immune response. Consistent with this, an increase in CIN, chronic cGAS-STING activation, and poorer patient survival rates are associated with metastatic rather than primary tumors.^13^

Additionally, a study indicates that STING activation in low immunogenic tumors can induce high expression of IDO in a type I IFN-dependent manner, promoting tumor growth, a phenomenon not observed in high immunogenic tumors.^221^ Interestingly, the expression of IDO is a result of type I IFN-mediated pathway reshaping. This complexity suggests that the impact of the cGAS-STING pathway extends beyond its immediate gene targets, influencing broader signaling networks within the tumor microenvironment. The differential reshaping of pathways in tumors with varying immunogenicity can also be attributed to the diverse activation levels of these pathways. In tumors with low immunogenicity, effector cells are unable to effectively kill tumor cells and expose their DNA, leading to a lower level of activation of the STING pathway. Conversely, in tumors with high immunogenicity, the STING pathway is activated at a higher level. The removal of CD8^+^ T cells, followed by the knockout of STING in tumors with low immunogenicity, leads to the elimination of STING’s inhibitory effect on these tumors.^221^ This finding demonstrates that effector cells play a role in STING-mediated tumor protection. The intricate interplay between the STING pathway and the immunogenicity of the tumor highlights the complexity of immune responses in cancer and underscores the importance of understanding these dynamics for effective cancer therapy.

The response to STING signals in the TME may depend on which cells respond and the intensity of that response. A study focusing on bladder cancer found that cGAMP stimulation of STING can drive the phenotypic transformation of cancer-associated fibroblasts via type I IFN. These transformed fibroblasts activate WNT5A expression through the IFNAR/STAT1 pathway, which in turn promotes β-Catenin expression in tumor cells, enhancing tumor stemness and leading to poor patient prognosis.^222^ The pro-tumoral role of fibroblasts in bladder cancer suggests that the downstream pathways mediated by the cGAS-STING pathway are cell-type-specific. In “cold” tumors lacking T cell infiltration, the primary responders to STING activation might be the stromal cells, potentially promoting tumor growth. Therefore, stratifying patients based on the composition of cells in the TME may benefit in selecting suitable candidates for STING agonist therapy. Additionally, exploring why different cell types exhibit diverse innate immune pathway signaling is also a point of interest.

##### cGAS-STING pathway and metastasis

The impact of the cGAS-STING pathway on tumor metastasis is one of the most contentious topics in this area. Given the pathway’s role in activating the immune system, it should inhibit tumor metastasis. Some studies have shown that tumor cells can selectively degrade extracellular cGAMP through the production of ectonucleotide pyrophosphatase-phosphodiesterase 1 (ENPP1), thereby preventing its transfer from cancer cells to immune cells and promoting tumor metastasis.^223^ In breast cancer, the inhibition of ENPP1 successfully suppressed tumor metastasis.^224^ However, evidence also exists that suggests the cGAS-STING pathway can promote metastasis. In brain metastatic tumors, migratory tumor cells form gap junctions with astrocytes, transferring cGAMP from the former to the latter. This activation of STING in astrocytes subsequently promotes the release of Type I IFN and TNF-α, which in turn activates the STAT1 and NF-κB signaling pathways to facilitate tumor growth.^225^ The diverse downstream effects triggered by the intercellular transfer of cGAMP and subsequent activation of the STING pathway may be dependent on the type of cells activated. Notably, the activation of STING in stromal cells by cGAMP tends to elicit pro-tumoral responses. For instance, mesenchymal stromal cells have been reported to drive lung metastasis in breast cancer mice receiving radiation therapy through the cGAS-STING-CCL5 pathway.^226^ Future research is required to elucidate the reasons for cell-type-specific variations in the cGAS-STING pathway. Additionally, it’s crucial to investigate whether it’s possible to manipulate this pathway to restore its anti-tumoral functionality. Understanding these dynamics could pave the way for more effective and targeted cancer therapies, leveraging the intricate interplay of the cGAS-STING pathway in different cellular contexts.

Another line of research focused on the cGAS-STING pathway in tumor cells characterized by chromosomal instability (CIN). Studies indicate that cGAS-STING can promote metastasis through the activation of non-canonical NF-κB pathways in a tumor cell-autonomous manner.^13^ Contrary to this, research on colon cancer revealed that CIN could promote metastasis in a cGAS/STING-independent fashion, and silencing cGAS/STING had no impact on tumor invasiveness.^227^ Therefore, the role of cGAS-STING in the metastasis of tumors may be tumor-type- or CIN-type-specific, necessitating further in-depth studies to clarify the role of the cGAS-STING pathway in tumor metastasis.

Moreover, recent studies have discovered that when cells at tumor metastasis sites are in a dormant state, using STING agonists can inhibit cancer cells from progressing from a quiescent state to aggressive metastasis in a manner dependent on NK cells and T cells.^228^ Interestingly, the STING pathway undergoes dynamic changes in its expression levels—downregulation, upregulation, and then downregulation again—during the stages of dormancy, proliferation, and macrometastasis, mediated by epigenetic regulation. This variation reflects the different levels of the STING pathway under various microenvironmental states.^228^ Such differential expression levels could be one of the reasons why latent metastatic cancers and advanced metastatic cancers have distinct responses to STING agonists. This phenomenon highlights the complexity of the STING pathway’s role in cancer progression and the importance of understanding its context-dependent behavior for developing effective cancer treatments, especially in targeting different stages of metastasis.

#### Role of cGAS-STING pathway in different types of cells

**Fig. 4:**
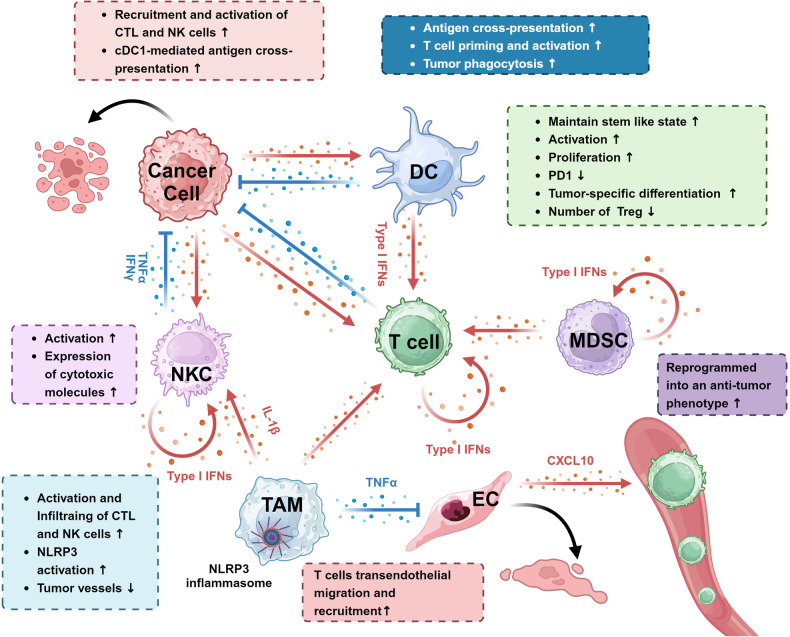
Anti-tumor cell interaction network following activation of the cGAS-STING pathway. The red arrows represent the promotion while the blue arrows represent the inhibition. Created with BioRender.com

Despite the key role of the cGAS-STING pathway in the initiation, progression, and metastasis of tumors, as previously mentioned, the exploration of its functions often focuses on global perturbations, such as using STING knockout models or STING agonists. This leads to the impact of the cGAS-STING pathway on various components within the TME, where they intermingle, making it challenging to discern the specific functions of each component in the TME. The complexity of interactions and the influence of the cGAS-STING pathway across different cell types create a multifaceted and dynamic environment, complicating the understanding of individual contributions to tumor behavior and response to therapies. The specific roles played by different cell types within the TME remain largely unexplored. Previous discussions have highlighted that the functionality of the cGAS-STING pathway may be cell-type specific. In reality, limited studies based on in vitro experiments, chimeric models, and cell-type-specific gene knockouts using systems like Cre-loxP have demonstrated the cell type-dependency of the cGAS-STING pathway in tumors. Different cells, each performing unique functions and communicating with each other, form a network, significantly impacting tumor behavior (Fig. 4).

##### Tumor cells

Activation of the cGAS-STING pathway within tumor cells can play an anti-tumor role. First of all, STING activation within the tumor can directly induce apoptosis of tumor cells.^192,209,210^ Moreover, the activation of the intrinsic cGAS-STING pathway in tumor cells can release type I IFN and cytokines including ISGs, regulating the immune microenvironment. This includes the release of CXCL10 and CCL5, inducing the recruitment and activation of CTL^229^; the release of CXCR3 ligands, such as CXCL10, enhancing the recruitment and cytotoxicity of NK cells,^230^ and promoting conventional (c)DC1s mediated antigen cross-presentation through IFN-I signaling.^231^ The type I IFN released upon STING activation can also regulate tumor cells themselves, enabling the expression of MHC-I.^231^

cGAMP formed after the activation of cGAS in tumor cells can be taken up by non-tumor cells in the TME, activating the STING pathway. For example, the communication of cGAMP could promote tumor killing by activating NK cells by producing downstream type I IFN. In this process, the expression of cGAS in tumor cells rather than in non-tumor cells is necessary for the NK cell-mediated anti-tumor effect.^123^

This evidence indicates that the intrinsic anti-tumor effects of the cGAS-STING pathway in tumors are based on its normal physiological functions, like inducing apoptosis, releasing pro-inflammatory, and transferring cGAMP. Therefore, ensuring the normal operation of the intrinsic cGAS-STING pathway in tumors is a necessary condition for it to exert its anti-tumor effects.

The aberrant STING pathway in tumor cells can paradoxically promote tumor progression. In tumors with chromosomal instability (CIN) characteristics, the STING pathway exhibits a weakened association with canonical NF-κB or type I interferon regulatory factors, while the association with non-canonical NF-κB target genes is enhanced. Consequently, the activation of the cGAS-STING pathway in tumor cells autonomously promotes cellular invasion and migration.^13^ Therefore, future applications targeting the STING pathway in tumor cells should be further stratified based on downstream pathway activity. This stratification would involve selectively using agonists or inhibitors, depending on the specific downstream pathway activated in the tumor context. This approach necessitates a deeper understanding of the molecular mechanisms at play and emphasizes the importance of personalized medicine in cancer treatment. By tailoring the intervention to the unique molecular landscape of each tumor, it becomes possible to exploit the full therapeutic potential of targeting the STING pathway.

##### Macrophages

Macrophages are the primary contributors to the production of cGAS-STING pathway-related cytokines, such as IFN and TNFα within the TME. Knockout of STING in macrophages results in a decrease in IFN-α and IL-6 induced by STING agonists.^208^ Furthermore, tumor-associated myeloid cells are the primary source of TNFα in the TME. They are the primary cells that recruit tumor-infiltrating CD8^+^ T cells to the TME. The TNFα released by the activation of STING in tumor-associated myeloid cells can stimulate the TNFα-TNFR1 pathway in endothelial cells, leading to apoptosis of these cells and thus leading to the destruction of the tumor blood vessels.^211^ The use of STING golden-ticket (point mutation of STING) mice and wild-type mice bone marrow chimeras demonstrates that chimeric tumor-bearing mice formed from Sting golden-ticket-origin bone marrow and wild-type mice nearly lose their ability to respond to STING-mediated cytokines, such as IFNγ. However, chimeric mice formed from wild-type-origin bone marrow and Sting golden-ticket mice retain this response-ability. This suggests that the production of anti-tumor cytokines following STING activation largely depends on the inherent STING pathway in bone marrow-derived hematopoietic cells.^232^

The inherent STING pathway in macrophages is also crucial for the crosstalk of different pro-inflammatory pathways. Studies using the Lyz2^Cre^; STING^flox/flox^ mouse model which specifically knocked out STING in myeloid cells have demonstrated that the intrinsic STING in macrophages can activate NLRP3, inducing the production of IL18 and IL1β. These, in turn, cause the expression of 4-1BBL in macrophages and 4-1BB in NK cells. The 4-1BBL-4-1BB co-stimulatory signal promotes the infiltration of NK cells and enhances their tumor-killing function, such as by increasing perforin expression and extending the survival of mice.^233^

A notable characteristic of macrophages is their potent phagocytic capability, which enables them to significantly enhance the source of cGAS-STING pathway agonists through phagocytosis. For instance, in diseases like multiple myeloma and Acute Myeloid Leukemia (AML), it has been observed that macrophages actively engulf tumor-derived mitochondrial DNA (mtDNA) or tumor cells themselves, thereby promoting the activation of the STING pathway.^234,235^

The intrinsic cGAS-STING pathway in macrophages is characterized by its potent downstream production of pro-inflammatory molecules, such as TNFα and IFNγ. Therefore, STING agonists are expected to produce a strong anti-tumor effect in macrophage-dominant tumors like gliomas. Indeed, many preclinical studies have confirmed this effect, but clinical validation is still lacking.^236^ However, it is important to consider the high heterogeneity of macrophages within the TME and their tendency to undergo tumor-promoting transformations. The possibility of reconfiguration or remodeling of this pathway during tumor development and progression is a significant aspect that warrants further investigation. This reconfiguration could potentially alter the effectiveness of STING-targeted therapies in clinical settings. Understanding these dynamics is crucial for developing effective strategies that leverage the anti-tumor potential of the cGAS-STING pathway in macrophages while minimizing the risk of enhancing tumor-promoting activities.

##### DCs

The specific knockout of STING in cDCs using zbtb46^Cre^; STING^flox/flox^ mice revealed that intrinsic STING in cDCs is necessary for the Th1 CD4^+^ T (IFN-γ^+^ CD4^+^ T) cell response induced by DNA vaccines and also for Th1 type IgG_2C_ antibody response.^237^ Moreover, DCs rely on their inherent STING pathway to effectively initiate IFN-dependent T-cell responses to tumor antigens.^238^ Research has shown that the inherent STING pathway in DC cells is indispensable for the activation of T cells by intracranial injection of virus-like particles delivering cGAMP.^208^

The phagocytosis of tumor cells by DCs is crucial in detection and clearance. Tumor cells can evade immune phagocytosis by expressing CD47.^239,240^ Blocking CD47 can inhibit tumor growth, and multiple studies have shown that the tumor-suppressing effect of CD47 blockade therapy depends on the inherent STING pathway in DC cells.^241–243^ After blocking CD47, DCs, rather than macrophages, preferentially ingest mitochondrial DNA derived from tumors, activating the cGAS-STING pathway, promoting type I IFN production and cross-priming, leading to the activation of CD8^+^ T cells.^241^

Besides priming and activating T cells, the autocrine IFN generated by the activation of STING is also crucial for the maturation and activation of DCs,^202^ and treatment with cGAMP can induce DC maturation.^211^

In conclusion, the intrinsic cGAS-STING pathway in DCs is indispensable for exerting DC cross-presentation and immune cell activation functions, and STING agonists are potent targets for stimulating DC functions. DCs exhibit a unique characteristic in their cGAS-STING pathway activation, specifically their active uptake of tumor-derived DNA. This ability facilitates spontaneous activation of their cGAS-STING pathway.

##### T cells

The STING pathway is crucial for maintaining T cell function. STING and IRF3 lacking in host cells fail to prime CD8^+^ T cells.^244^ In STING knockout mice, CD8^+^ T cells exhibit reduced priming, diminished cytokine polyfunctionality, and an impaired capacity for secondary expansion.^238^ The intrinsic cGAS-STING pathway in T cells can restrain tumor growth, as evidenced by accelerated growth of some tumors, including GL261, in tumor-bearing mice after specific knockout of STING in T cells using the CD4^Cre^; STING^flox/flox^ mouse model.^206,245^ Furthermore, activation of cGAS-STING is essential for CD8^+^ T cells to differentiate and maintain a stem-like state under chronic tumor antigen stimulation. Intrinsic cGAS-STING in T cells can promote TCF1 expression by inhibiting Akt activity, thereby preserving the ‘stemness’ of CD8^+^ T cells, promoting the preservation of CD8^+^ central memory T cell populations, and preventing differentiation towards a terminal exhaustion-like subset, thereby preserving the anti-tumor potential of T cells. STING agonists have been found to sustain human stem-like central memory CD8^+^ T cells, promoting human CAR-T cell therapy in mice.^206^

Interestingly, STING appears to have a role in promoting the differentiation of CD4 + T cells towards a Treg direction. In cervical cancer, CD4^Cre^; STING^flox/flox^ mice carrying tumors exhibited slower tumor growth tendencies and fewer FOXP3^+^ cells but a higher proportion of CD8^+^ cells in the tumor tissue. Further studies found that tumor-derived exosomes (containing TGF-β, cGAS, and cGAMP) can induce FOXP3 transcription, leading to the proliferation of induced Treg, via TBK1-IRF3-mediated SMAD3 and STAT5 phosphorylation, independent of IFN-β.^246^

The dual function of intrinsic STING in T cells for tumor development may be related to different activation levels within T cells in different TMEs because STING’s effect on T cells is dose-dependent. Long-term over-activation of endogenous STING or high-dose STING agonists can reduce T cell responses and impair T cell proliferation.^232,247^ For T cells, varying levels and durations of pathway activation might result in different pathway connections similar to tumor cells. However, there’s currently a lack of quantitative research in this area, particularly concerning the specific level of activation that might trigger a functional transformation in the cGAS-STING pathway of T cells. Understanding this aspect is crucial, as it could provide essential insights into the optimal dosing of STING agonists for therapeutic purposes. Additionally, combination therapy may indeed represent a strategy to enhance the safety and efficacy of immunotherapies targeting the cGAS-STING pathway in T cells. By understanding and subsequently blocking pro-tumoral pathways, it is possible to ensure that the cGAS-STING pathway in T cells is steered towards anti-tumor activity.

##### NK cells

STING agonists can directly stimulate NK cells, promoting the production of cytokines such as IFN-β, TNF-α, and IFN-γ, and the expression of cytotoxic molecules, thus enhancing NK activation.^209^ NK cells may also be the primary cell type in STING agonist-induced anti-glioma responses, as the anti-glioma effect induced by STING agonists is abolished after depleting NK cells with anti-NK1.1 antibodies.^212^ These findings suggest that in gliomas, specifically targeting the cGAS-STING pathway in NK cells could potentially eliminate interference from innate pathways in other cell types, thereby inducing a potent anti-tumor immune response.

##### MDSCs

Intrinsic STING has been reported to reprogram MDSCs. After STING activation, the autocrine type I IFNs reeducate MDSCs into an anti-tumor phenotype that acquires the ability to activate CD8^+^ T cells.^248^ It is important to note that in MDSCs, the PKR-like endoplasmic reticulum (ER) kinase (PERK) can inhibit the activation of the cGAS-STING pathway.^248^ Therefore, when targeting the cGAS-STING pathway in MDSCs for therapeutic purposes, attention must be paid to this aberrant suppression within the pathway. Overcoming this inhibition is crucial for the effective application of cGAS-STING pathway-targeted therapies in these cells.

##### Endothelial cells

The STING pathway in endothelial cells aids in the recruitment of T cells. Using inducible endothelial-cell-specific-deficient (Cdh5^Cre/ERT2^; STING^flox/flox^) mice, researchers have found that T-cell recruitment in response to TNF-α-induced inflammation is significantly reduced. Endothelial cells generate type I IFNs through STING activation, which in turn activate downstream CXCL10 via the type I IFN/IFNAR pathway. The interaction between CXCL10 and CXCR3 expressed in T cells facilitates T-cell recruitment and plays a pivotal role in T-cell transendothelial migration.^249^

##### B cells

Interestingly, the intrinsic cGAS-STING pathway in B cells demonstrates the negative regulatory effect on immunity. The specific knockout of endogenous STING in B cells through CD19^Cre^; STING^flox/flox^ mice increased the numbers of antigen-specific plasma cells and antibody levels.^250^ In the PDAC model, it was also found that the activation of intrinsic STING in B cells can increase the frequency of IL-35^+^ and IL-10^+^ Breg cells, thereby reducing the frequency of NK cells and impairing anti-tumor immunity. Consistent with this, after B cell-specific knockout of STING, the survival time of mice is significantly prolonged.^245^ The activation of the STING pathway in B cells and its surprising role in promoting tumors necessitates further investigation into the state of the classical cGAS-STING pathway in B cells and the reasons for its suppression.

In summary, the cGAS-STING pathway exhibits cell type-specific functions, which may be suitable to their unique gene expression profile. Interestingly, the cGAS-STING pathway can have contradictory impacts on tumor development even within the same cell type. This complexity is likely influenced by the state of the pathway in cells within different tumor environments and the response of the tumor to these conditions.

Currently, it is encouraging that research based on chimeric models or gene editing technologies on the autonomous functions of the cGAS-STING pathway in cells is being actively pursued. However, it is regrettable that few studies focus on the autonomous functions of the cGAS-STING pathway in specific cell types at different stages of tumor development. Commonly, research involves the deletion of the cGAS-STING pathway in specific subtypes of cells before or at the early stages of tumor formation, with scant exploration of its functions in the middle or later stages of tumors. As some intriguing clinical trials have shown, in the later stages of tumor development, agonists of the cGAS-STING pathway, acting as unbiased perturbations to the entire tumor, paradoxically have little effect on tumor progression.^251,252^ This is contrary to the significant anti-tumor tendency of the autonomous cGAS-STING pathway in immune cells.

Therefore, it is crucial to further refine the analysis of the cGAS-STING pathway’s functions in the same cell type under different states and environmental conditions. This nuanced understanding is essential for bringing breakthroughs in precision cancer therapy, highlighting the importance of considering the diverse and complex roles of this pathway in various tumor contexts.

#### Abnormal regulation of cGAS-STING pathway in tumor

The functionality of the cGAS-STING pathway in the TME is compromised through various mechanisms, rendering it ineffectual. In multiple tumors, including colorectal cancer and melanoma, the expression of cGAS or STING is epigenetically silenced.^253–255^ Recent reports also reveal that in GBM, the promoter region of STING is methylated, consistent with the dysfunctional cGAS-STING pathway in glioma cell lines.^212,256,257^ Interestingly, this silencing is not observed in tumor-associated immune cells or the extracellular matrix.^256^ Aberrant protein expression in tumors also drives this process. For instance, in *KRAS-LKB1* mutated lung cancer, the loss of LKB1 leads to a pronounced silencing of STING expression. This is partially attributed to the hyperactivation of DNMT1 and EZH2, enzymes associated with epigenetic modifications, induced by elevated levels of S-adenylmethionine.^258^ Interestingly, this particular type of cell also limits the accumulation of cGAMP within the cell and is especially sensitive to the accumulation of cGAMP. Compared to cells with *KRAS-Tp53* mutations, these cells can more effectively activate the STING pathway at equivalent levels of cGAMP. Consequently, this sensitivity becomes a vulnerability, which can be exploited by promoting STING expression and the accumulation of cGAMP to enhance cell death.^259^ Aside from epigenetic silencing, mutations in cGAS or STING genes have been observed in various human tumors, resulting in impaired induction of pro-inflammatory cytokines.^255^

Abnormal protein interactions within the TME also contribute to the inhibition of the cGAS-STING pathway. For example, aberrantly amplified proteins such as HER2 can recruit and bind to AKT1, inhibiting the regular binding of TBK1-STING and thereby weakening STING-mediated signaling to suppress anti-tumor immunity.^260^ The tumor cell-intrinsic cGAS-STING pathway also has negative regulation of anti-tumor cells. For example, the enhancement of STING pathway ubiquitination degradation mediated by Galectin-9 in tumor cells can promote the expression of IL-1β and IL-6, thereby promoting the expansion of MDSCs.^261^ In GBM, under the influence of TME, the binding between PP2Ac and MST1/2 in TAMs cannot be dissociated by cGAMP. As a result, MST1/2 cannot be released to phosphorylate YAP. This inhibits the degradation of YAP. YAP then acts as an inhibitor of TBK1 and IRF3, which are downstream of the STING pathway, thereby inhibiting IFN production.^262^ Furthermore, it has also been observed that the high expression of PCBP2 (Poly(rC) Binding Protein 2) in GBM is able to inhibit the cGAS-STING pathway by binding to cGAS.^263^

Additionally, in cervical cancer, the human papillomavirus (HPV), a key causative agent, produces oncoproteins E1A and E7 that bind to STING, inhibiting its functionality, thereby undermining host immunity and facilitating tumorigenesis.^264^ Beyond directly affecting STING, mutations within the tumor can also interfere with other proteins in the cGAS-STING pathway. Mutant P53, for example, can bind to TBK1, preventing the formation of the TBK1-STING-IRF3 trimeric complex and thereby disrupting downstream IFN production.^265^

Post-translational modifications also regulate the cGAS-STING pathway. The DAPK3 (death-associated protein kinase 3) modulates the ubiquitination status of STING, maintaining steady-state levels of STING protein and promoting STING-TBK1 interaction. The observed loss of DAPK3 in certain tumors drives immune escape.^266^

In the TME, microRNAs (miRNAs) also play a role in regulating the cGAS-STING pathway. For instance, in breast cancer, the expression of miR-93 inhibits STING expression, thereby promoting tumor proliferation.^267^ Moreover, miRNA interference with STING is implicated in tumorigenesis. During the development of ovarian cancer, miR-181a suppresses STING, mediating the transformation of fallopian tube secretory epithelial cells. This leads to the expansion of cells with high genomic instability, thereby promoting tumor formation.^268^

Metabolic abnormalities also affect the STING pathway. Recent research has found that in tumor-infiltrating DCs, aerobic glycolysis can promote the production of ATP in DCs, thereby facilitating the activation of STING.^269^ However, the low-glucose environment typical of tumors inhibits the activation of STING in DCs. Another study has shown that metabolic substrates like glucose can act as a signaling molecule to inhibit the cGAS-STING pathway. Glucose achieves this by binding to the methyltransferase NSUN2, promoting its oligomerization and activation. Activated NSUN2 further stabilizes TREX2, an exonuclease, which then limits the accumulation of cytoplasmic double-stranded DNA and, consequently, the activation of the cGAS-STING pathway.^270^

It is noteworthy that tumors can also inhibit the activation of the cGAS-STING pathway in immune cells by suppressing the intercellular transfer of cGAMP, thereby promoting metastasis.^223^

All these findings underscore that rectifying the dysfunctional cGAS-STING pathway is a critical consideration in the therapeutic application of STING agonists.

### Toll-like receptor pathway

Toll-like Receptors (TLRs) are members of the PRRs family, with ten different types expressed in humans.^271,272^ They are expressed in various types of immune cells and can recognize different patterns of PAMPs and DAMPs, such as DNA, RNA, and LPS (lipopolysaccharides).

#### Signaling function of TLR pathway

##### Signal transduction

Apart from TLR3, Toll-like receptors (TLRs) activate the NF-κB pathway or the MAPK pathway through a MyD88-dependent pathway, leading to the expression of pro-inflammatory cytokines. However, TLR3 and TLR4 can activate the NF-κB pathway or the MAPK pathway through a MyD88-independent pathway, namely TRIF.^267^ Interestingly, TLRs are also involved in the activation of IRF3, IRF5, and IRF7^273,274^ (Fig. 2).

##### Regulation of metabolism

TLRs are involved in regulating metabolism. TLR downstream TBK1 can also activate the kinase AKT, leading to the phosphorylation of hexokinase and the induction of glycolysis.^275^ This triggers metabolic changes, such as increased glucose utilization and ATP production, thereby promoting protein synthesis and secretion.^275^ Early research has demonstrated that in CD8 + T cells, the absence of TRAF6, a key adapter protein in the TLR pathway, can lead to defects in fatty acid metabolism. This metabolic alteration results in impairments in the immune memory function of T cells.^162^ Additionally, in vitro experiments have observed that TLR4, by inducing the activation of the TRAF6-STAT3 axis, promotes mitochondrial respiration in macrophages. This leads to the accumulation of tricarboxylic acid (TCA) cycle metabolites, which play a key role in the production of inflammatory cytokines.^276^ For instance, succinate, one of the TCA cycle metabolites, can enhance the expression of IL-1β.^277^

Interestingly, the activation of TLRs can also reverse the immunosuppressive functions of tumor-associated cells through metabolic regulation. In vitro studies targeting CD4+ Tregs have found that activating TLR8 can decrease glucose uptake by downregulating GLUT1/3 and suppress glycolysis by downregulating the mTOR signaling pathway, which leads to reduced expression of genes and proteins related to glucose metabolism. This metabolic reprogramming results in the decrease of Treg-mediated suppression of effector T cell proliferation. This indicates that the immunosuppressive function of Tregs is reversed through TLR8 activation.^278,279^ In macrophages, the activation of TLR9 can promote FAO and initiate a shift away from the complete utilization of carbon from glucose towards glutamine anaplerosis, which is used for generating TCA cycle intermediates. This metabolic shift empowers macrophages to overcome the “don’t eat me” signal transmitted by the CD47 molecule on tumor cells.^33^

In summary, TLRs play a significant role in regulating cellular metabolism. This regulation can alter the supply of energy and the molecular substrates of biochemical reactions within cells. Consequently, the activation of TLRs holds the potential to reshape immune cells into an anti-tumor phenotype.

##### Epigenetic regulation

As mentioned above, the impact of TBK1 on metabolic activation can also suppress mitochondrial respiration and the tricarboxylic acid cycle, generating ample acetyl-CoA moieties to promote histone modification.^280,281^ This represents a path worth exploring in cancer research. Indeed, evidence has been found in tumors that TLR signaling modulates epigenetic changes affecting tumor development. For example, in TAMs, it has been discovered that TRIF-mediated signaling in TLRs can induce high levels of extracellular signal-regulated kinase (ERK)-1/2 MAPK phosphorylation. This leads to ERK-1/2-dependent histone phosphorylation at the IL-10 promoter, promoting high expression of IL-10 and consequently causing immune suppression.^282^

Beyond histone modifications, TLRs also influence DNA methylation. In colorectal cancer cells, activation of TLR4 can lead to the activation of the nuclear transcription factor nuclear factor of activated T cells 5 (NFAT5), which in turn promotes the expression of JmjC-domain-containing histone demethylase 2B (JMJD2B). JMJD2B causes demethylation at the promoter of core stemness transcription factors Nanog homeobox (NANOG), thereby promoting the expression of NANOG, which enhances tumor stemness.^283^ Additionally, in colon cancer cells, TLR4 mediates immunosuppressive Siglec-7 ligands’ promoter methylation through the NF-κB pathway, leading to epigenetic silencing of its expression.^284^

These findings underscore the complex role TLRs play in cancer, impacting immune responses and tumor progression through various epigenetic mechanisms. Understanding these processes is vital for developing new cancer therapies. This understanding introduces a new dimension to the mechanisms by which TLRs regulate tumor progression, and exploring these pathways could potentially lead to novel therapeutic approaches.

Downstream signaling molecules of TLRs can modify the expression of regulatory genes through histone modifications and methylation changes. This expands the regulatory scope of the TLR signaling pathway. Future research can delve into more epigenetically regulated genes and explore the differences and similarities in epigenetic regulation under tumor and physiological conditions. This exploration will enhance our understanding of how TLRs influence gene expression beyond traditional signaling pathways, particularly in the context of immune responses and cancer development.

##### Induction of non-coding RNAs

TLRs can also interfere with non-coding RNAs (ncRNAs) functions. For instance, TLR signaling can induce lincRNA-Cox2. LincRNA-Cox2 is a type of long ncRNAs that can interact with the SWI/SNF chromatin remodeling complex to promote the expression of inflammatory factors, or work with hnRNPA2/B1 to inhibit chemokine gene transcription.^285^ Some lncRNAs can also regulate the NF-κB and MAPK pathways.^286^ Research has found that the activation of TLR4 in hepatic progenitor cells -derived myofibroblasts can induce the upregulation of specific lncRNAs, which in turn regulates the expression of the *epidermal growth factor receptor (EGFR)* and *phosphatase and tensin homolog (PTEN)*. This regulation promotes the proliferation and malignant transformation of these cells.^287^ In addition, the activation of TLRs can influence the levels of miRNAs, which, in turn, can affect the transmission of TLR signals, targeting proteins and transcription factors along the pathway.^288^ In pancreatic cancer in vitro experiments, it has been discovered that the activation of TLR4 decreases the level of miR-29C through the NF-κB pathway. Consequently, this reduction in miR-29C levels leads to an increase in its inhibitory target, MMP-9, which promotes tumor invasion.^289^

##### Regulation of inflammasome and cell death

TLRs also participate in the regulation of inflammasomes. Firstly, TLRs can regulate the expression of inflammasome components. On the one hand, TLR activation can stabilize HIF-1α through metabolic changes, thereby inducing the expression of pro-IL-1β, an important component of the inflammasome.^290^ On the other hand, the NF-κB pathway controlled by TLR can promote the expression of NLRP3 and pro-IL1β.^291^ Furthermore, TLR activation can mediate the rapid assembly of the NLRP3 inflammasome through IRAK1.^292^ The downstream signals of TLR can also affect the activity of NLRP3 by participating in its post-translational modification.^293^ In addition, TLRs can mediate non-canonical inflammasome activation, whereby the signal transduction of TRIF can promote the expression of caspase-11, leading to limited proteolysis of gasdermin D, facilitating IL-1 release and pyroptotic cell death.^294,295^ TRIF can recruit RIPK1 and RIPK3, which can cause RIPK3-dependent programmed necrosis and the activation of the MLKL-dependent necroptotic death pathway by RIPK1/RIPK3 complexes when caspase-8 is blocked.^296^

#### Role of TLRs in tumor

##### TLR pathway and tumorigenesis

TLRs influence tumorigenesis by regulating inflammation. The activation of TLRs in innate immunity plays a crucial role in immunosurveillance, eliminating aberrant cells.^297^ TLRs also participate in DNA repair mechanisms, thereby suppressing tumor formation. Specifically, studies have demonstrated that TLR4 enhances the expression and function of the DNA repair protein Ku70, thereby inhibiting chemically induced hepatocarcinogenesis in a mouse model.^298^

Conversely, tumors can exploit the activation of their TLRs to suppress apoptosis and promote proliferation via pathways such as NF-κB and MAPK.^299^ This has been corroborated in various types of tumors, including gliomas, hepatocellular carcinoma, lymphoma, and breast cancer.^299–303^ Additionally, these pathways facilitate the secretion of factors like IL-6, GM-CSF, and MMPs, promoting immune escape and a pro-tumoral niche.^299,302,304,305^ Inflammation induced by TLR-associated pathways also stands as one of the risk factors for various cancers.^306^

Numerous natural ligands for TLRs originate from microbes. For example, TLR1/2 and TLR2/6 detect lipoproteins and lipopeptides, while TLR4 is activated by LPS and TLR5 by flagellin.^307^ Therefore, in tissues with natural microbial communities, such as the gastrointestinal tract and lungs, microbial components can influence tumorigenesis by modulating tissue inflammation through TLR activation. For instance, *Fusobacterium nucleatum* potentiates intestinal tumorigenesis via a TLR4/p-PAK1/p-β-catenin S675 cascade.^308^ Gastric cancer is closely related to *Helicobacter pylori* infection, which stimulates innate immune responses via TLRs, activating the NF-κB pathway to induce the COX-2/PGE2 cascade. This subsequently promotes the secretion of a series of pro-tumorigenic molecules like IL-11, CXCL1, CXCL2, and CXCL5. Moreover, the TLR2/MyD88 axis also maintains cancer stemness.^309^ *Helicobacter pylori* also activates TLR9 to promote gastric inflammation and hyperplasia, thereby inducing the onset of gastric cancer.^310^ Conversely, alterations in microbial populations mediated by TLRs can also influence tumorigenesis. For example, the absence of TLR6 leads to a loss of gut *Lactobacillus*, which promotes anti-apoptotic activity in tumor cells.^311^

The impact of TLRs on tumorigenesis remains inconclusive and is likely influenced by multiple factors, such as the inflammatory status of the tissue and the microbial community.

##### TLR pathway and tumor progression

The phenomenon of TLRs negatively regulating tumor growth was observed quite early on. For example, at the end of the 19th century, William Coley observed that repetitive injections of a mixed bacterial toxin from Gram-positive *Streptococcus pneumoniae* and Gram-negative *Serratia marcescens* effectively combated tumors.^297^ An intriguing clinical prospective study revealed that postoperative infections in GBM patients improved their prognosis, with the median survival period for those with infections extending to 30 months, significantly exceeding the 15 months seen in the control group.^312^ These results either directly or indirectly suggest the role of microbial components in anti-tumor activities.

Currently, we understand that the activation of TLRs can effectively trigger downstream pathways such as NF-κB, MAPK, and IRF3/5/7, inducing an array of inflammation-regulating molecules like Type I IFNs, TNF-α, and IL-1.^313^ These molecules reshape the TME and exert anti-tumor effects through multiple pathways (Table 1). Notably, the activation of TLRs can: (1) Enhance dendritic cell secretion of Type I IFNs and upregulate co-stimulatory molecules, thereby increasing their antigen-presenting capabilities.^314,315^ (2) Foster T-cell recruitment, activation, and proliferation while reducing inhibitory molecule expression.^316,317^ (3) Activate NK cells to kill tumor cells.^318,319^ (4) Reprogram macrophages to an anti-tumor phenotype.^320,321^ (5) Inhibit recruitment of immune-suppressive cells like Tregs and MDSCs, even converting them to an anti-tumor phenotype.^322–324^ (6) Induce various forms of tumor cell death, such as apoptosis, autophagic cell death, and necrosis, thereby promoting tumor clearance.^325–327^

Consistently, in certain types of tumors, the higher expression levels of TLRs are correlated with better patient prognosis. Specifically, low expression of TLR9 portends an unfavorable prognosis in cases of TNBC and Renal Cell Carcinoma.^328,329^ Moreover, robust expression of TLR2 and TLR4 is an independent predictor for a more favorable prognosis in patients with stage I-II PDAC.^330^

Conversely, some evidence suggests that TLR activation may contribute to tumor progression. Clinical data indicate that elevated expression of TLR4 is associated with poor prognosis in patients with liver and colorectal cancer.^331,332^ Similarly, high expression levels of TLR9 have been linked to unfavorable outcomes in patients with liver cancer, glioma, HCC, and esophageal adenocarcinoma.^331,333,334^ Preclinical studies indicate that activation of TLRs can generate pro-tumorigenic inflammatory signals and survival cues for tumor cells. For instance, TLR1-mediated activation of NF-κB is associated with the upregulation of anti-apoptotic molecules such as BCL-2 and survivin.^335^ Moreover, activation of TLR3 has been found to promote cancer stemness.^336^ In cancer stem cells (CSC), it has been reported that TLR activation elicits anti-apoptotic and proliferative pathways through upregulation of NOS2, COX2, and EGFR ligand.^337^ Additionally, existing research suggests that TLR activation can sculpt a tumor-promoting microenvironment by fostering angiogenesis and reconfiguring the extracellular matrix to favor invasion and metastasis.^163,338,339^

Scrutinizing these divergent results, the cell-type-specific functions of TLRs play a crucial role. The anti-tumor activation of immune cells and pro-tumor existence in tumor cells coexist under the influence of TLR agonists. Further research is needed to delineate the downstream pathways of TLRs in different cell and tumor types at various developmental stages to activate TLR pathways associated with anti-tumor activities selectively.

The important feature of the TLR signaling pathway is its ability to respond to various microbial components in the environment. The presence of the gut microbiota may result in cells being in a chronically activated state for an extended period. Considering the influence of the gut on various distant organs, an interesting avenue for exploration is the impact of chronic TLR activation in the gut on tumor development in distant organs. The heterogeneity of the gut microbiota could be one of the reasons for these contradictory results.

##### TLR pathway and tumor metastasis

Evidence suggests that TLR activation in malignant cells can facilitate tumor metastasis through various pathways. In NSCLC, cancer cells can enhance cell adhesion and metastasis via the TLR4-MAPK-ERK1/2 pathway.^340^ Additionally, the inhibition of TLR4 in cancer cells has been shown to reduce tumor metastasis in colon and gallbladder cancer, potentially by attenuating the activation of the NF-κB pathway.^341,342^ Furthermore, HCC cells can promote tumor migration and epithelial-mesenchymal transition (EMT) through the TLR4/STAT3 axis.^343^ EMT refers to the biochemical changes that epithelial cells undergo to acquire a mesenchymal, motile phenotype. This transition serves as the initial step in tumor dissemination.^344^ Activation of TLR7 in tumor cells also mediates the infiltration of MDSCs thus promoting the expression of genes related to invasion and metastasis, such as ICAM-1 (intercellular adhesion molecule 1), cytokeratins 7 and 19, thereby enhancing EMT.^345^

TLR activation within other tumor-associated cells also contributes to tumor metastasis. Evidence from infection models suggests that epithelial TLRs can encourage tumor dissemination. Gram-positive and negative bacteria can stimulate bronchial epithelial TLR2 or TLR4 respectively, thereby enhancing the adhesion of NSCLC cells to liver sinusoids via IL-6 secretion, ultimately promoting liver metastasis.^346,347^ Activation of epithelial TLR4 also fosters the recruitment of PMN-MDSCs, setting the stage for pre-metastatic niches.^348^ Furthermore, activation of TLR4 in M2 TAMs can induce EMT in pancreatic cancer cells partially through IL-10 signaling.^349^ In lung epithelial cells, TLR3 can be activated by RNA from exosomes derived from primary tumors. This activation induces the secretion of chemokines in the lungs, which in turn promotes the recruitment of pro-tumoral neutrophils, thus forming a pre-metastatic niche.^350^

In contrast to these findings, TLR agonists can also suppress tumor metastasis. For instance, TLR7/8 agonists stimulate dendritic cell activation and the production of type I IFNs and other pro-inflammatory cytokines, thereby initiating T-cell cytotoxicity and enhancing tumor immunogenicity, which inhibits TNBC metastasis.^351^ Activation of anti-tumor cell TLRs has also demonstrated anti-metastatic effects. Endogenous activation of TLR3 in NK cells stimulates IFN-γ production and enhances NKG2D-mediated cytotoxicity, independently of dendritic cells, thereby suppressing tumor metastasis.^352^ TLR5 agonists activate anti-tumor immunity through the NK-dendritic-CD8^+^ T-cell axis.^353^ Moreover, the presence of NK cells is indispensable for the inhibitory effects on melanoma liver metastasis mediated by TLR5 agonists.^354^

These findings suggest a dual role for TLR activation in promoting and inhibiting tumor metastasis. The influence of TLR activation seems to vary depending on whether it occurs in pro-inflammatory like CTLs, DCs, and NK cells, or anti-inflammatory cells such as MDSCs and M2 TAMs. Therefore, strategies that selectively target pro-inflammatory cells or increase their presence in the TME may be one of the most effective approaches for maximizing the anti-metastatic effects of TLR agonists. More importantly, it is crucial to consider the reasons behind the cell-type heterogeneity in the TLR pathway. Whether this cell-type heterogeneity in the TLR pathway exists physiologically or is influenced differentially by tumors during the process of tumor development needs to be explored. Discussing this issue will contribute to our understanding of the role played by TLRs in the evolution of tumors. Especially in metastatic tumors, they exhibit differential tissue characteristics. Whether these distinct tissue backgrounds contribute to shaping different TLR pathways is a question that needs to be addressed. The answer to this question will impact our approach to medication. If the TLR pathways of the same cells in different tissue niches have varying effects on tumors, it may limit the systemic application of drugs.

#### Role of TLR pathway in different types of cells

##### Tumor cells

Tumor cells ubiquitously express various types of TLRs.^355^ The activation of TLRs within tumor cells can autonomously induce cell death through multiple pathways.

Inherent TLRs in tumor cells can induce apoptosis to exert anti-tumor effects directly. For example, the activation of TLR9 promotes apoptosis in the glioma.^325^ In hepatocellular carcinoma (HCC), activation of TLR3 can downregulate anti-apoptotic proteins such as Bcl-xL and survivin, and synergistically augment TRAIL-induced apoptosis, thereby promoting cancer cell death.^356^ Stimulation of TLR3 in tumor cells derived from human head and neck cancer has also been found to downregulate the anti-apoptotic protein survivin.^357^ Additionally, both TLR4 and TLR7 have been implicated in the direct induction of cellular apoptosis. In acute monocytic leukemia, malignant monocytic cells undergo apoptosis in a TLR4-dependent manner.^358^ Moreover, Bacillus Calmette-Guérin (BCG) can directly induce apoptosis in urothelial carcinoma cells by activating TLR7.^359^ Interestingly, in vitro experiments have shown that the activation of TLR4 in lung cancer cells can inhibit TNF-α or TRAIL-induced apoptosis via the NK-κB pathway.^360^ This suggests that the induction of tumor cell apoptosis by TLRs is dependent on the type of tumor.

Furthermore, the activation of TLRs can indeed induce cellular autophagy.^361^ However, its anti-tumorigenic efficacy remains a subject of debate.^362^ For example, the TLR7 agonist imiquimod can induce autophagic cell death in melanoma cells during radiotherapy, inhibiting tumor growth.^326^ However, it has been reported that autophagy triggered by TLR3 or TLR4 activation enhances the production of various cytokines. This ultimately promotes the migration and invasion of radiation-induced lung cancer cells.^363^

Besides apoptosis and autophagy, TLRs also induce necroptosis in tumor cells. For example, TLR3 has also been reported to induce ROS-mediated necroptosis of tumor cells via the TLR3-TICAM-1-RIP3 axis.^327^ Cellular processes such as apoptosis, autophagy, and necrosis are characterized by the generation of DAMPs. Therefore, the cell death induced by TLRs in tumors is likely to impact the innate immune pathways of surrounding tumor-associated cells in vivo. The distinction in the TME may account for the differential roles of TLR in different tumors. Investigating how the heterogeneity of the TME contributes to the downstream effects of TLR-induced cell death in tumor cells is a direction worth exploring.

Other evidence also raises caution about the pro-tumorigenic roles of intrinsic TLR pathways in tumor cells. For instance, the activation of TLR4 in melanoma cells fosters tumor progression and metastasis.^364^ Conversely, the knockout of TLR2 in intestinal epithelial cells hinders the onset and development of tumors.^365^ In non-small cell lung carcinoma (NSCLC), tumor cells expressing TLR7 promote cancer progression and metastasis by recruiting bone marrow-derived immunosuppressive cells.^345^ These findings suggest that the impact of TLRs on tumor cells may be contingent upon both the specific type of TLR and the classification of the tumor involved, with the molecular expression characteristics of different types of tumors potentially causing diverse crosstalk with the TLR pathway, leading to a variety of downstream effects. Therefore, further analysis of the signaling interaction network is needed to clarify the relationship between TLRs and TLR-induced cell death in cancer cells.

##### Macrophages

Monocytes strongly express TLR2, intermediate levels of TLR1 and TLR4, and moderate levels of TLR5, TLR6, and TLR8,^366^ while microglia, significantly express TLR2 and TLR3, while also expressing TLR1, TLR4, TLR5, TLR6, TLR7, and TLR8, along with low levels of TLR9 at the mRNA level.^367^

Activation of TLRs can promote anti-tumor polarization of macrophages. In vitro experiments have shown that TLR4 activation can promote the polarization of macrophages towards the M1 phenotype via the TLR-4/MyD88/NF-κB pathway.^368^ Furthermore, in a mouse model of peritoneal carcinomatosis of colorectal cancer, a high-fat diet was found to promote M1 polarization of adipose tissue macrophages (ATMs) by activating TLR4. The intrinsic knockout of TLR4 in macrophages significantly reduced the expression of TNFα, IL-1β, and CXCL10 induced by a high-fat diet. Activation of the TLR4-CXCL10 axis in ATMs can also promote the recruitment and activation of T cells, leading to tumor suppression.^369^ Another preclinical study has demonstrated similar findings. Activation of TLR4 promotes the reprogramming of M2 TAMs to the M1 phenotype, increasing the expression of TNFα, IL1β, and CCL3, and damaging IL4/STAT6-dependent M2 polarization through the TLR4/NF-κB pathway.^320^ TLRs also promote the recruitment of macrophages to tumors. In a melanoma model, it was found that the absence of the downstream molecule MyD88 in macrophages impaired the recruitment of F4/80^+^ CD11b^+^ macrophages to tumors.^370^

The activation of macrophage intrinsic TLRs can promote its antigen presentation ability. For example, TLR2 activation in microglia increases the expression level of MHC-I, thus, promoting the proliferation of CD8^+^ T cells. Furthermore, the activation of TLR4 in macrophages can lead to a reduction in the expression of microRNAs targeting MHC-I chain-related protein A (MICA), thus upregulating MICA expression. MICA is part of a group of NKG2D ligands that can interact with NKG2D in NK cells, leading to NK cell activation.^371^

MyD88 in myeloid cells also inhibits tumorigenesis. The absence of MyD88 activated the Wnt and STAT3 pathways, resulting in more β-catenin gene mutations and less DNA repair, thereby promoting the development of colon cancer.^372^ TLRs in myeloid cells remodel the TME. The deletion of MyD88 in myeloid cells caused an increase in Foxp3^+^Tregs, and IL-1β-producing neutrophils, and reduced IFN-γ expression by CTL.^372^

Interestingly, the intrinsic activation of TLR4 or TLR2 within macrophages can facilitate metastasis through modifications of the extracellular matrix (ECM) by downstream products. The activation of TLRs/Myd88/TRAF6 can induce SIRT2 (Sirtuin 2) to undergo autophagosome translocation, leading to its release into the ECM where SIRT2 promotes cancer cell metastasis, presumably by deacetylating multiple extracellular proteins, including ITGB3 (Integrin Subunit Beta 3) and collagens.^373^

In summary, macrophages exhibit a unique expression profile of TLRs. Limited studies have shown that intrinsic TLRs in macrophages, such as TLR4, can promote the polarization of macrophages towards the M1 phenotype and enhance their antigen-presenting capabilities. The integrity of the MyD88-mediated TLR pathway is crucial for maintaining the intrinsic functions of TLRs. The absence of MyD88 can induce tumor development. But paradoxically, autophagy regulation mediated by the TLR/MyD88 pathway can promote tumor metastasis. Whether this dual action exists in all TAMs or is dependent on specific characteristics of TAMs is an important aspect of understanding the role of the intrinsic TLR pathway function in TAMs. And how these different pathways are constructed in macrophages is worth investigating.

##### DCs

Different types of DCs have distinct TLR expression profiles. pDCs primarily express TLR1, TLR6, TLR7, and TLR9, while cDCs primarily express TLR1, TLR6, and TLR8.^366^

TLRs are crucial for the anti-tumor functions of DCs.^374^ DC maturation depends on the TLR signaling pathway, and activation of TLR4 or TLR7/8 can induce DC maturation.^375,376^ The loss of the TLR adapter molecule MyD88 results in poor DC maturation, characterized by significantly reduced proliferation and survival capabilities of antigen-specific naive CD4^+^ T cells. Moreover, the lack of MyD88 also impairs the ability of DCs to polarize antigen-specific naïve CD4^+^ T cells to the Th1 phenotype.^377^

Activation of TLR4 not only induces DC maturation but also promotes the proliferation of CD8^+^ T cells mediated by DCs.^378^ Besides, TLR4 can upregulate the expression of co-stimulatory molecules and inhibit phagolysosome fusion of DCs, thereby enhancing cross-presentation and promoting the priming of CD8^+^ T cells.^376^ In addition to influencing DC-T cell interactions, TLRs also impact the anti-tumor immunity of NK cells. After specifically disrupting the TLR pathway in DC cells via MyD88^flox/flox^; CD11c^Cre^, it was found that the production of IL-12 and type 1 IFN in DC cells was significantly reduced, and IL-12 is crucial for NK activation.^379^ Consistently, The TLR3-TRIF axis in DC cells can promote DC-NK cell contact and activation, leading to the regression of tumors with low MHC expression.^380^

The activation of DC-intrinsic TLRs can also favor ablation of tolerogenic cDC1s, which show high expression of B- and T-lymphocyte associated/attenuator (BTLA). This killing mechanism is autonomous to DCs and the activation of DC-intrinsic TLR3 can induce BTLA^hi^ DCs to express and release a significant amount of TNFα. This TNFα, mediated through TNF receptor 1 (TNFR1) on BTLA^hi^ DCs, leads to their death.^381^ Notably, while the source of TNFα does not affect its cytotoxic effect on DCs, BTLA^hi^ DCs exhibit higher expression of TNFR1 compared to BTLA^lo^ DCs, thereby making TNFα selectively lethal to the immunosuppressive BTLA^hi^ DCs. This suggests that the cell type-specific functions of TLRs partially depend on different cell types’ distinct responses to downstream effector molecules of TLR signaling but not only the signaling itself.

In summary, TLRs play a multifaceted role in immune regulation, particularly within the TME. On one hand, TLRs are involved in modulating the distribution of DC subgroups, leading to a reduction in suppressive and immature DC subpopulations. On the other hand, DC-intrinsic TLRs enhance the anti-tumor functions of immune cells like T cells and NK cells through interactions with these immune cells. Specifically targeting the TLR pathway of DCS tends to produce an overall antitumor effect.

##### T cells

Analyses of human tonsillar T cell subsets have demonstrated a broad expression of TLRs, with TLR1, TLR2, TLR5, TLR9, and TLR10 being particularly prominent. CD4^+^ T cells exhibit high expression of TLR1 and TLR9, while CD8^+^ T cells predominantly express TLR2, TLR3, TLR4, and TLR5.^382,383^ Moreover, the regulatory T cells, CD4^+^ CD25^+^ Tregs, express TLR4, TLR5, TLR7 and TLR8 at high levels.^384^

The activation of TLRs has a profound influence on the activity of naïve CD4^+^ T-cells. In vitro experiments indicate that the activation of TLR3 and TLR9 can promote their survival^385^ while the activation of TLR2 promotes their differentiation towards Th17.^386^ Specifically, TLR2 enhances the production of IL-2 and Th1-associated cytokines, while reducing the inhibitory cytokine IL-10.^387^ Additionally, the activation of TLR5 and TLR7/8 augments the proliferation of CD4^+^ T-cells and the production of cytokines such as IFN-γ, IL-8, and IL-10.^388^ TLRs also play a pivotal role in fostering T-cell memory. Co-stimulation of TLR2 with IL-2 or IL-15 in vitro amplifies the proliferation of CD4^+^ memory T-cells.^389^

TLRs participate in reversing the exhausted phenotype of T-cells. Activation of TLR2 in CD4^+^ T-cells notably elevates the expression of T-bet, IFN-γ, IL-2, and the anti-apoptotic molecule Bcl-2, while concurrently reducing the expression of PD-1 and LAG-3. Besides, the ability to activate B-cells are also enhanced in TLR2-activated CD4^+^ T cell.^390^

The shared adapter molecule MyD88, common to both IL-1R and TLRs, plays a pivotal role in the activation of CD4^+^ T-cells. Utilizing genetically engineered mice with a CD4^cre^; MyD88^flox/flox^ genotype, it was observed that an inherent deficiency of MyD88 in T cells leads to diminished T cell proliferative responses, along with a significant reduction in the cellular secretion of IFN-γ and IL-17. IL-1, induced by intrinsic TLR activation in naïve CD4^+^ T cells, can promote Th1 differentiation, counteracting the effects of regulatory T cells, via the inherent IL-1/IL-1R/MyD88 pathway within these cells.^391^

As for CD8^+^ T-cells, activation of either TLR2 or TLR5 enhances their cytotoxic activity and upregulates the expression of IFN-γ, TNF-α, and granzyme B. These two TLRs also amplify the proliferation of T-cells and exhibit a synergistic effect.^392^ In vitro studies also reveal that activation of TLR1/2 in tumor-specific CTLs augments their proliferative capacity and cytotoxicity.^393^ Notably, TLR2 on CD8 T-cells also participates in lowering the threshold for TCR signaling activation, enabling these cells to effectively generate memory cells even in response to weak TCR signals.^394^

Furthermore, TLRs play a role in modulating the functionality of Treg cells. Activation of TLR1/2 in Foxp3^+^ Tregs is capable of impairing their immunosuppressive function.^393^ Activation of TLR8 in CD4^+^ Tregs can reverse their immunosuppressive function via the TLR8-MyD88-IRAK4 axis. TLR8 ligand-stimulated Treg cells, when adoptively transferred to tumor-bearing mice, enhance anti-tumor immunity.^395^

In summary, the activation of intrinsic TLR signaling pathways in T cells tends to generate anti-tumor effects, and the specific phenotype of these effects is related to the differentiation type of T cells. Exploring how this heterogeneity of TLR signaling pathways is established during the differentiation process of T cells is a direction worth investigating.

##### Neutrophils

TLRs play a role in the immunosuppression mediated by TANs. Research has found that Annexin A2, derived from NSCLC, can activate the TLR2/MYD88 pathway in neutrophils, leading to the expression of ARG1, which induces immunosuppression. The ability of tumor secretions to induce ARG1 is completely lost in neutrophils isolated from mice with TLR2 or MyD88 knockouts.^396^

##### NK cells

NK cell-autonomous TLR signaling directly contributes to their tumor-killing effect. NK cells strongly express TLR1 and moderately express TLR2, TLR3, TLR5, TLR7/8, and TLR9.^366,397^ Activation of TLR2-5 and TLR7/8 can induce NK cells to produce a large amount of IFN-γ and stimulate cytotoxicity, but TLR9 cannot.^398–402^ The stimulation of TLR3 can restrict tumor metastasis in an NK cell-dependent manner.^352^ In the presence of IL-12, in vitro experiments have demonstrated that poly (I:C) can activate TLR3 and TLR9 in NK cells, inducing the surface expression of activation markers including CD69 and CD25, generating high levels of IFN-γ, TNF-α, and GM-CSF, and enhancing their lytic capacity against tumor cells. Moreover, these cells also acquire the ability to kill immature dendritic cells.^403^

##### Endothelial cells

The activation of TLRs in endothelial cells has been demonstrated to promote tumor metastasis. Mouse models of breast and lung cancer revealed that endothelial cells can induce the expression of the axon-guidance gene *Slit2* via TLR3, which facilitates the migration of cancer cells towards endothelial cells and intravasation.^404^ Moreover, studies in melanoma have reported that the activation of TLR4 in endothelial cells within the lungs can drive metastasis by releasing G-CSF and CXCL5, thereby recruiting PMN-MDSCs to lung tissue and creating a pro-metastatic niche.^348^

As mentioned, TLRs exhibit cell type-specific functions, and although most TLRs use MyD88 as their adapter molecule, research indicates that these functions are also dependent on the subtype of TLR. Currently, studies on TLR functions in different cell types in vivo largely rely on the Cre-loxP system. However, research often focuses on the specific deletion of MyD88, with insufficient attention to the loss of different TLR subtypes, impeding our understanding of the diverse roles of TLRs in cancer.

While there are many studies using mouse models with specific TLR subtype knockouts to investigate TLR functions, the broad-spectrum nature of these knockouts can introduce confounding effects. For example, systemic application of TLR7/8 agonists can increase the number of cDCs in the bone marrow. Interestingly, the specific knockout of MyD88 in DC cells by Zbtb46^Cre^; Myd88^flox/flox^ does not affect the increase in the cDC population, suggesting that TLR signaling pathways in other cells play a role in the expansion of the cDC population.^405^

Therefore, gene editing models that knock out specific receptors within specific cell types are crucial for understanding the intricate mechanisms of innate immune pathways in cancer. This approach is a key focus for future research, as it allows for a more precise dissection of the roles of TLRs and their signaling pathways in different cellular contexts within the TME.

#### Abnormal regulation of TLR pathway in tumor

The expression levels of TLRs tend to be elevated in tumor cells,^406^ and their intracellular distribution undergoes changes within the TME.^407^ Normally, TLR2, TLR4, and TLR5 are predominantly expressed on the cell membrane; however, during malignant transformation, their distribution shifts increasingly toward intracellular locations.^407^ For instance, in healthy esophageal columnar epithelium, TLR5 is primarily basolateral, but it extends into the cytoplasm and loses its polarity in the atypical proliferative epithelium.^408^ This cytoplasmic bias in expression is reminiscent of the altered intracellular localization of TLRs’ endogenous ligands, DAMPs, within tumor cells. For example, the DAMP molecule HMGB1 is overexpressed in the cytoplasm of tumor cells and can activate TLR2, TLR4, and TLR9.^409^ This could initiate downstream pathways that promote tumor invasion and metastasis via mechanisms such as immune suppression and angiogenesis.^410^

The fluctuating expression levels of TLRs are regulated by various proteins within the tumor. For example, the negative regulator of TLR, TOLLIP (toll-interacting protein), is downregulated in colorectal and gastric cancers, thereby contributing to the upregulation of TLR expression.^411,412^ Moreover, cytokines such as TNF-α, glucocorticoids, IL-6, and IFN-γ in the TME also enhance TLR expression.^413,414^ miRNAs play a pivotal role in this process. In chronic lymphocytic leukemia, IL-6 elevates miR-17 and miR-19a, targeting TLR7 and TNFA mRNA, inducing tumor cell tolerance to TLR7 agonists.^415^ In patients with colorectal carcinoma, downregulation of miR-143 has been observed, which facilitates the upregulation of TLR2 expression, thereby promoting tumor cell migration.^416^ Interestingly, metabolism also plays a role in regulating TLR expression. For instance, in colorectal cancer (CRC), palmitic acid upregulates TLR4 expression in CRC cells in a PU.1-dependent manner (where PU.1 binds to the TLR4 promoter). And this upregulation finally leads to faster tumor growth.^417^

Therefore, TLR expression in tumors is modulated through a multitude of pathways. Targeting these pathways in concert may provide an effective strategy to optimize the activation levels of TLRs within the TME effectively.

### NOD-like receptor pathway

Nucleotide oligomerization domain (NOD)-like receptors (NLRs) are cytosolic cellular sensors that play a crucial role in infection and autoinflammation. NLRs can be subdivided into four families: NlRA, NLRB, NLRC, and NLRP, performing different function.^418^

#### Signaling function of NLR pathway

##### Inflammasome formation

Inflammasomes are large multimolecular complexes that consist of a cytosolic sensor, such as NLR, an adapter protein speck-like protein containing a CARD (ASC), and an effector pro-caspase-1.^419^ The NLRP3 inflammasome is one of the most extensively studied inflammasomes. Upon recognition of PAMPs/DAMPs by NLRP3, NLRP3 molecules dimerize and activate ACS via homophilic CARD–CARD interaction. Subsequently, pro-caspase-1 binds to ASC via the CARD-CARD domain to complete the formation of the inflammasome, which activates caspase-1.^420^ Caspase-1 can cleave pro-IL-1β and pro-IL-18 into active IL-1β and IL-18. Caspase-1 also initiates pyroptosis by cleaving gasdermin D.^420^

##### Signaling transduction

The NLR family plays a crucial role in signal transduction within the immune system. The activation of NOD1 and NOD2 can trigger the RIPK2-mediated activation of the NF-κB and MAPK signaling pathways, promoting the transcription of pro-inflammatory genes.^421^

##### Transcription activation

The NAIP family member CIITA and the NLRC family member NLRC5 (CITA) can respond to IFN-γ, mediating the expression of MHC II and MHC I molecules, respectively.^422,423^ Given the essential role of MHC I and MHC II in the immune response, interfering with CIITA and NLRC5 may have positive effects on anti-tumor immunity. There are currently attempts to use CIITA as an adjuvant for constructing anti-tumor vaccines.^424^

##### Induction of autophagy

NLRs can mediate autophagy targeting intracellular bacteria and viruses. NOD1 and NOD2 can recruit ATG16L1 to the site of pathogen invasion to mediate autophagy.^425^ NLRX1, located in mitochondria, regulates virus-induced autophagy through interaction with mitochondrial Tu translation elongation factor (TUFM).^426^ NLR-mediated autophagy has been linked to tumorigenesis. Specifically, NOD2 promotes the onset of liver cancer by facilitating the degradation of key proteins involved in DNA repair through autophagy.^427^

#### Role of NLR pathway in tumor

##### NLR pathway and tumorigenesis

Research on the impact of NLRs on tumorigenesis has largely focused on inflammation-induced tumors, particularly in the case of colon cancer. NLRs modulate the proliferation and survival of tumor precursor cells, such as intestinal epithelial cells, through various pathways. For instance, NOD2 can suppress colon inflammation by inducing the downregulation of TLRs through IRF4, which in turn diminishes the TLR4-MAPK/NF-κB/STAT3 inflammatory pathway.^428^ NLRP12 also serves an anti-inflammatory function by negatively regulating non-canonical NF-κB and MAPK-mediated inflammation.^429^ Furthermore, NLRs can directly induce apoptosis; NAIPs, for example, promotes apoptosis in aberrantly proliferating intestinal epithelial cells, thereby inhibiting tumor formation.^430^ NLRC4 has been shown to inhibit the proliferation of colonic epithelial cells and promote apoptosis through the caspase-1 pathway, thereby suppressing the onset of colorectal cancer.^431^ NLRs also influence inflammation-induced tumorigenesis through their effects on immune cells. Intra-cellular activation of NOD1 in T cells enhances the production of the tumor-suppressing cytokine IFNγ, thereby inhibiting tumorigenesis.^432^

In inflammation-induced tumors, inflammasomes regulated by NLRs exert a modulatory effect partially through the release of IL-1β and IL-18. For instance, the NLRP1 inflammasome-modulated secretion of IL-1β and IL-18 by non-hematopoietic derived cells, likely intestinal epithelial cells, help to maintain the integrity of the epithelial barrier in the colon, thus suppressing tumor formation in inflammation-related mouse colon carcinogenesis models.^433,434^ Besides, IL-18 promotes the secretion of IFN-γ, which subsequently activates the STAT1 pathway in tumor cells to exert a tumor-suppressive effect.^435^

However, the influence of IL-1β and IL-18 on tumorigenesis is still debatable and may vary depending on the tumor context. In gastric cancer, for example, NLRP3-mediated IL-1β has been shown to promote tumorigenesis.^436^ Conversely, in lymphomas, NLRP3-mediated IL-18 promotes lymphoma cell proliferation and inhibits apoptosis by upregulating *c-MYC* and *BCL-2* expression, while downregulating *TP53* and *BAX* expression.^437^

In addition to their complex roles in suppressing tumorigenesis, NLRs have also been implicated in promoting tumorigenesis. Beyond IL-1β and IL-18, NLRP3 can promote colon tumorigenesis and proliferation by enhancing the transcription of cyclin-D1.^436^ Moreover, NOD2 has been reported to play a role in promoting hepatocarcinogenesis. Activation of NOD2 in liver cells by PAMPs originating from gut microbiota can enhance hepatic inflammation through Rip2-dependent activation of MAPK, NF-κB, and STAT3 pathways. Additionally, NOD2 translocates to the nucleus, where it interacts directly with nuclear lamina components such as lamin A/C, facilitating their proteolytic degradation. This dual action—both inducing inflammation and promoting genomic instability—contributes to liver cancer onset.^427^ In lymphomas, a high frequency of CIITA gene alterations has been observed and is associated with poor patient prognosis. These alterations in the CIITA gene result in the downregulation of surface HLA class II expression and overexpression of PD-L1 and PD-L2. Consequently, this diminishes the immunogenicity of the tumor and suppresses antitumor immunity.^438^

Interestingly, the same receptors or downstream effector molecules may exert opposing roles in tumorigenesis. This may be attributed to different pathway preferences in varying TMEs. Additionally, the contrasting roles may also stem from different regulatory factors inducing the tumors. For instance, in colitis-associated cancers, inflammation serves as a driving factor, whereas in other types of cancer like hepatocellular carcinoma, resistance to inflammation may lead to defects in immune surveillance.

##### NLR pathway and tumor progression

In tumor development, NLR signaling can serve a tumor-suppressive role (Table 1). For instance, NOD1 activation in breast cancer has been observed to induce apoptosis in tumor cells and reduce their sensitivity to estrogen-induced proliferative stimuli.^439^ In colorectal cancer, NLRC3 has been shown to inhibit cell growth mediated by the PI3K-AKT signaling axis and mTOR, while stabilizing tumor suppressor proteins FoxO3a and FoxO1. Furthermore, NLRC3 mitigates tumor cell stemness by downregulating the expression of stemness-associated genes such as BMI1 and OLFM4.^440,441^ In gastric cancer, NLRP6 promotes senescence and clearance of tumor cells through the P14ARF–Mdm2–P53 pathway.^442^ NLRs can also enhance the immunogenicity of tumors; NLRC5 enhances MHC-I expression, thereby augmenting CD8^+^ T cell activation and cytotoxicity,^443,444^ while CIITA boosts MHC II expression, facilitating the induction of tumor-specific CD4^+^ T cells.^445^ Additionally, NLRs modulate the anti-tumor activity of immune cells; in bladder cancer, for example, NLRP3 promotes the reprogramming of TAMs to an M1 phenotype.^446^

Conversely, NLRs have also been implicated in tumor progression (Table 1). They directly affect the characteristics of tumor cells; upregulation of NLRP3 in glioma cells promotes cell growth, apoptosis, and metastasis through the PTEN/AKT signaling pathway, and its expression correlates with an increased WHO grade.^447^ NLRs can also modulate the TME to foster tumor progression. For example, in breast cancer under obese conditions, activation of myeloid cell NLRC4 induces IL-1β release, promoting adipocyte-mediated VEGFA expression and angiogenesis.^448^ In a mouse model with a high-cholesterol diet, cholesterol crystals activate NLRP3 in macrophages, inducing IL-1β secretion and facilitating tumor development.^449^ In pancreatic cancer, NLRP3 signaling in macrophages directs the differentiation of CD4^+^ T cells into tumor-promoting Th2, Th17, and Treg cell subsets while suppressing Th1 cell polarization and CD8^+^ T-cell activation.^450^ NLRP3 also promotes the expansion and recruitment of MDSCs to melanoma, inhibiting the function of CTLs.^451–453^ The absence of NLRP3 has been observed to enhance the recruitment of NK cells in invasive breast cancer.^454^

In a word, NLRs play multifaceted roles in tumor progression depending on the context and the specific molecular pathways involved.

##### NLR pathway and tumor metastasis

Many studies suggest that NLR-mediated chronic inflammation can drive tumor metastasis. The activation of NLRs within tumor cells can directly enhance their metastatic potential. For instance, the activation of NOD1 in tumor cells enhances cell adhesion functions through p38 MAPK, promoting the interaction of circulating tumor cells with the extracellular matrix and thus facilitating metastasis.^455^ In oral squamous cell carcinoma, activation of NLRP3 downregulates the epithelial adhesion marker E-cadherin and upregulates mesenchymal markers such as vimentin and N-cadherin.^456^ Intriguingly, in colon cancer, upregulation of NLRP3 could promote EMT in an inflammasome-independent manner. This may be mediated by the upregulation of Snail1, which in turn inhibits the adhesion molecule E-cadherin.^457^ The activation of NLRs in tumor cells can also shape the pre-metastatic niche at distant sites through the secretion of specific molecules. For instance, activation of NLRP3 in melanoma cells can lead to the release of HSP70, which stimulates the recruitment of PMN-MDSCs in lung epithelial cells.^348^

The intrinsic NLR pathways within immune cells also regulate tumor metastasis. In colorectal cancer, activation of NOD1 in macrophages promotes hepatic metastasis.^455^ Besides NOD1, activation of NLRP3 in TAMs also increases the migratory capacity of colon cancer cells through the secretion of IL-1β, promoting liver metastasis.^458^

Conversely, another study demonstrated that IL-18 secretion mediated by NLRP3 in hepatic macrophages inhibits liver metastasis of colorectal cancer by promoting the maturation and function of NK cells.^459^ These conflicting viewpoints might be reconciled by considering different contexts: the former emphasizes the pro-metastatic role of NLRP3 in primary tumors, whereas the latter underscores its anti-tumor effects at metastatic sites. NLRP3 may exert different functions depending on the tissue-specific characteristics at the site of metastasis and the original tumor, as well as the different stages of tumor development.

#### Role of NLRs in different types of cells

##### Tumor cells

The tumor-intrinsic NLR pathway mainly mediates anti-tumor immunity through two mechanisms. Firstly, it can induce cell death directly. Many members of the NLR family are constituents of the inflammasome. For instance, the NLRP3 inflammasome can induce pyroptosis mediated by GSDMD, which has been found to induce apoptosis in a variety of tumor cells, including non-small cell lung cancer (NSCLC) and gastrointestinal tumors.^460,461^ The suppression of NLRP3 can lead to resistance in NSCLC to the drug gefitinib, an EGFR inhibitor.^462^ Secondly, the activation of the NLR pathway reshapes the immune microenvironment through the secretion of pro-inflammation cytokines such as IL-1 and IL-18.^463^ The IL-1 induced by NLRP3 inflammasomes could activate the DCs and enhance their cross-priming ability, while IL-18 participates in tumor suppression by inducing the production and activation of the tumor suppressors IFN-γ and STAT1.^435,464^

However, activation of tumor intrinsic NLRs also participates in promoting tumor progression and metastasis. In human oral squamous cell carcinoma, NLRP3 is overexpressed compared to normal oral mucosal epithelial cells and is correlated with the tumor size and lymphatic node metastatic status. In vitro and in vivo experiments have demonstrated that the knockdown of NLRP3 in tumor cells not only inhibits cellular proliferation but also suppresses the migratory capabilities of these cells partially through the upregulation of EMT-related gene.^456^ Additionally, in metastatic cervical squamous cell carcinoma cells, the upregulation of NOD1 and NOD2 expression promotes proliferation, invasion, and migration. Mechanistically, this is partly mediated through the activation of NF-κB and ERK signaling pathways, as well as enhanced IL-8 secretion.^465^

The intrinsic NLR pathways within tumor cells also contribute to tumor development and metastasis by facilitating the recruitment of immunosuppressive cells. For instance, the activation of NLRP3 in melanoma cells stimulates the recruitment of PMN-MDSCs, mediating immune suppression and encouraging in situ tumor growth.^451^ Furthermore, the activation of NLRP3 can trigger the release of HSP70, which in turn activates TLR4 in distant lung epithelial cells. The latter then promotes the recruitment of PMN-MDSCs in lung tissue through the release of G-CSF and CXCL5, thereby creating a pro-metastatic niche.^348^

##### Immune cells

In immune cells, NLRs can orchestrate a reprogramming of the immune cell phenotype. For instance, the activation of NLRP3 aids in promoting the polarization of macrophages towards the M1 type.^446,466^ Experiments conducted in vitro have indicated that the knockout of NLRP3 in macrophages encourages a shift to the M2 phenotype, significantly diminishing ROS production, and consequently fostering tumor growth. The removal of NLRP3 also leads to a reduced phagocytic ability in M1 macrophages. Furthermore, preclinical data show that specific knockout of NLRP3 in myeloid cells enhances the growth and metastasis of endometrial cancer.^467^ TAMs can mediate anti-tumor immunity by generating active caspase-1 and IL-1β. The inhibition of the NLRP3 inflammasome in TAMs compromises the therapeutic response to cisplatin chemotherapy which may be attributed to the impaired production of IL-1β by TAMs.^468^

Additionally, the activation of the NLRP3 inflammasome in DCs, through the production of IL-1β and IL-18, amplifies stem-like CD8^+^ T cells and strengthens antigen-specific anti-tumor immunity of CD8^+^ T cells.^469^ NLRP3 can activate bone marrow dendritic cells, and by secreting IL-1β, it promotes the differentiation of CD4^+^ T cells into Th1 cells, which then exert anti-tumor effects by secreting IFN-γ.^470^ Besides, the release of IL-1β from DCs also primes IFN-γ–producing CD8^+^ T cells in an IL-1β dependent manner.^471^

However, the activation of NLRs in immune cells may also contribute to tumor progression, which is likely dependent on the type of tumor and cells involved. For instance, in pancreatic carcinoma, the NLRP3-IL10 signaling axis in macrophages drives the differentiation of CD4^+^ T cells into tumor-promoting Th2 cells, Th17 cells, and Treg populations, while simultaneously inhibiting Th1 cell polarization and cytotoxic CD8^+^ T cell activation.^450^ NOD1 activation in TAMs has also been reported to facilitate hepatic metastasis of colorectal cancer through the secretion of cytokines such as IL-6, CCL1, and CCL2.^472^ Furthermore, in gliomas, a specific subtype of high-grade glioma-associated microglia has been observed to promote glioma progression by secreting IL-1β, which is mediated by the NLRP1 inflammasome via the APOE pathway.^473^

In a specific subtype of high-grade glioma-associated microglia, tumor progression is promoted via the APOE/NLRP1/IL-1β axis.^473^ The activation of NOD1 in macrophages initiates the secretion of inflammatory cytokines (such as IL-6) and chemokines (such as CCL1 and CCL2), promoting the hepatic metastasis of colorectal cancer.^472^ The expression of NLRP3 in the TME disrupts the function of CD8^+^ T cells by activating IL-18.^474^

#### Regulation of NLR pathway in tumor

The expression of NLRs undergoes alterations in various types of cancer and correlates with patient prognosis. For instance, elevated expression of NLRP3 is observed in colorectal cancer and is linked to poor clinical outcomes.^475,476^ In glioma, NLRP3 has been positively correlated with higher histological grades.^447^ Multiple mechanisms are involved in the regulation of NLRs. In colorectal cancer, 5-HT plays a role in promoting NLRP3 activation. Tumor cell-derived 5-HT activates the HTR3A ion channel receptor on TAMs, leading to a Ca2^+^ influx, followed by the phosphorylation and activation of CaMKIIα. This subsequently induces NLRP3 phosphorylation and inflammasome assembly. The NLRP3 inflammasome activates IL1β, which stimulates *tryptophan hydroxylase 1* (*TPH1*, gene coding the 5-HT biosynthesis rate-limiting enzyme) transcription to increase 5-HT production in colorectal cancer cells, forming a positive feedback loop between 5-HT and NLRP3 signaling. Suppression of TPH1 effectively inhibits the progression of colorectal cancer.^477^ Additionally, estrogen receptor (ER) β upregulates the NLRP3 inflammasome via the MAPK pathway. In liver cancer, the downregulation of ERβ leads to the inhibition of the NLRP3 inflammasome, thereby promoting the progression of liver cancer.^478^

Non-coding RNAs also participate in NLR regulation. In prostate cancer, circAR-3—a circular RNA (circRNA) derived from the androgen receptor gene—mediates NLRP3 acetylation by KAT2B, promoting inflammasome assembly and advancing tumor progression.^479^ Both lncRNAs and miRNAs are implicated in NLR regulation as well. In NSCLC, neutrophil extracellular traps downregulate the expression of lncRNA MIR503HG, activating the NF-κB/NLRP3 pathway to promote EMT and NSCLC metastasis.^480^ Tumor cells deliver exosomal miR-21 that represses macrophage *PTEN* and *BRCC3*, facilitating NLRP3 phosphorylation and lysine-63 ubiquitination, thus inhibiting NLRP3 inflammasome assembly. This repression enhances cisplatin resistance in TAMs.^468^ Moreover, during gastric carcinogenesis, *Helicobacter pylori* suppresses the expression of miR-22 in gastric mucosa, weakening its inhibitory effect on NLRP3 and thus promoting gastric cancer progression.^436^

In summary, the dysregulation of NLRs in cancer serves as a driving force in tumor progression, making the pathways involved in these regulatory mechanisms potential targets for therapeutic intervention.

### RIG-I-like receptor pathway

The retinoic acid-inducible gene-I (RIG-I)-like receptors (RLRs) family is a protein family of cytoplasmic viral RNA detectors, consisting of three members: Retinoic acid Inducible Gene 1 (RIG-I), melanoma differentiation-associated factor 5 (MDA5), and laboratory of genetics and physiology 2 (LGP2).^481^ RIG-I recognizes short 5′ tri-phosphorylated double-strand RNAs (dsRNAs), single-strand RNAs (ssRNAs) forming secondary structures, as well as RNAs with uncapped diphosphate groups at the 5’ end. Meanwhile, MDA5 and LGP2 identify long dsRNA.^482^ RLR activation can be triggered by both viral and host-derived RNAs.^483^ Notably, LGP2 does not directly participate in signal transduction but can regulate RIG-I and MDA5 signaling in a concentration-dependent manner.^484^ Specifically, it promotes MDA5 signal activation at low concentrations and inhibits RIG-I and MDA5 signaling at high concentration.^484^

#### Signaling function of RLR pathway

##### Signaling of RLR pathway

The activation of RLRs mainly involves the NF-κB pathway and cell apoptosis. After RNA binds to RLRs, they interact with the adapter protein mitochondrial antiviral-signaling protein (MAVS), located on the mitochondrial outer membrane, peroxisome, or mitochondrial-associated membrane. This interaction forms prion-like filaments and activates TRAF, TBK1, and IKK kinases.^482^ Subsequently, the nuclear translocation of primary transcription factors IRF3/7 and NF-κB is activated, leading to the expression of type I IFN and other genes, such as ISGs.^481^

##### Promotion of apoptosis

The activation of MAVS leads to the expression of pro-apoptotic genes *Noxa* and *Puma*, whose expression products cause the cleavage of caspases 3, 7, and 9 into active forms, ultimately leading to cell apoptosis.^485,486^

#### Role of the RLRs in tumor

RLRs influence tumorigenesis. A diminished expression of RIG-I promotes the onset of HCC.^487,488^ During HCC onset, the demethylase JMJD4 acts on RIG-I, inhibiting necrotic inflammation and the subsequent onset of liver cancer induced by nonalcoholic steatohepatitis.^487^ Furthermore, RLR activation can suppress tumor development through multiple pathways. (1) Activation of RIG-I and MDA5 in tumor cells induces immunogenic cell death, promoting antigen presentation by DCs and activation of CTLs.^489–492^ (2) RIG-I activation can also inhibit the stemness of tumor cells.^493,494^ (3) RLR agonists promote IFN secretion, activating cytotoxic cells against the tumor.^489,490,495^ (4) The activation of RLRs enhances the cytotoxic ability of NK cells.^496^ (5) Stimulating RLRs leads to an increase in ROS formation in endothelial cells and the release of a significant amount of pro-inflammatory cytokines, causing endothelial dysfunction.^497^

Moreover, RLRs can sensitize the effects of radiotherapy and immunotherapy checkpoint inhibitors.^490,498,499^ An in vitro study demonstrated that the RLR pathway is necessary and sufficient for the cytotoxic response and IFN-β production triggered by radiotherapy and chemotherapy.^498^ Activation of RLRs increases PD-L1 expression in tumor cells, establishing sensitivity to PD-1 checkpoint blockade in vivo.^490^

Preliminary clinical applications of RLR agonists have demonstrated tumor control (Table 1). For instance, using the RIG-I receptor agonist SLR14 results in an increased population of CD8^+^ T lymphocytes, NK cells, and CD11b^+^ cells in B16 Melanoma, suppressing tumor growth and subsequently generating immune memory and a systemic anti-tumor response.^500^ In a mouse model of breast cancer, the RIG-I receptor agonist SLR20 was observed to increase tumor-infiltrating lymphocytes and trigger the extrinsic apoptosis pathway and pyroptosis in breast cancer cells, mitigating tumor growth and metastasis.^501^

Interestingly, the recognition of 5′-triphosphate RNA by RIG-I is largely independent of the RNA sequence. This allows for the concurrent activation of RIG-I and gene silencing by creating single short interfering RNA (siRNA) with 5′-triphosphate ends (3p-siRNA).^502^ For example, siRNAs targeting the anti-apoptotic gene *Bcl-2* or *TGF-β1* have exhibited enhanced anti-tumor effects in preclinical studies compared to treatments that merely stimulate RIG-I.^502,503^

Nevertheless, there are some contrasting views on the pro-tumorigenic role of RLRs. For example, increased RIG-I expression in T cells suppresses T-cell survival and anti-tumor cytotoxicity.^504^ Increased expression of RIG-I in PDAC promotes tumor growth and is associated with a poor prognosis in patients.^505^ LGP2, upon stimulation by radiotherapy-induced IFNβ during radiotherapy, can be overexpressed. This high expression of LGP2 can shield tumors from radiation damage by silencing the expression of IFNβ.^506^

This evidence suggests that variations in RLR expression levels regulate tumor progression and may be distinct from the direct activation effects of RLRs. The impacts of post-RLR activation on tumor progression warrant more in-depth exploration.

#### Role of RLR pathway in different types of cells

##### Tumor cells

Intrinsic activation of RLRs within tumor cells exerts diverse effects. Firstly, the activation of intrinsic RIG-I and MDA-5 in tumor cells can directly induce cell apoptosis, independent of type I IFNs.^489–491^ Significantly, RIG-I-mediated apoptosis is observed predominantly in tumor cells, while non-malignant cells are shielded from this pro-apoptotic signaling through the expression of Bcl-xL.^491^ This selective action underscores the therapeutic potential of targeting RIG-I in cancer treatment, as it may offer a safety margin by sparing normal cells from apoptotic induction. Additionally, in AML, RIG-I has been shown to induce AML cell death through the suppression of the Srt-AKT-mTOR signaling pathway and subsequent autophagy induction.^493^

The activation of intrinsic RLRs in tumor cells also enhances immune recognition and activation. Compared to tumor cell death mediated by chemotherapy or mechanical damage, apoptosis induced by RIG-I triggers caspase-3-mediated immunogenic cell death, characterized by the release of HMGB1 and translocation of calreticulin to the outer cell membrane. This process facilitates the effective phagocytosis of apoptotic tumor material by APCs, especially DCs, and subsequent activation via cross-presentation to prime CD8^+^ T cells.^490,492^ Importantly, intrinsic RIG-I signaling and caspase-3-mediated programmed tumor cell death are essential for the success of anti-CTLA-4 immunotherapy.^490^

Tumor cells often evade CD8^+^ T-cell recognition by downregulating MHC-I expression. The activation of intrinsic RIG-I in tumor cells induces MHC-I expression, thereby promoting CD8^+^ T cell-mediated recognition and killing.^502,507^ In human melanoma, RIG-I activation enhances MHC-I expression primarily through two pathways: IFNβ-mediated intrinsic expression and an IFN-independent IRF1 and IRF3 salvage pathway.^507^

Intrinsic activation of RIG-I in tumor cells can also promote cellular differentiation and reduce tumor stemness. For instance, the knockdown of RIG-I in human HCC cell lines induces increased stemness of HCC cells.^494^ In AML, RIG-I restrains leukemic stemness by inhibiting Srt-induced AKT-mTOR activation, even without foreign RNA priming.^493^

Furthermore, activation of RIG-I and MDA-5 mediates the production of type I IFNs, which contribute to the recruitment and activation of immune cells.^489,490,495^ Furthermore, RIG-I can amplify IFN-α effector signaling by promoting the activation of STAT1. In HCC, the low expression of RIG-I is significantly associated with a shorter survival period in patients and a poorer response to IFN-α treatment.^488^ Intrinsic RIG-I also limits the release of pro-tumorigenic cytokines; for example, the knockdown of RIG-I in HCC cell lines enhances TGF-β1 secretion, weakening the monocyte-to-DC differentiation and fostering the production of immune-tolerant, tumor-infiltrating DCs.^494^

RIG-I’s anti-tumorigenic properties also show a noteworthy advantage of being hypoxia-insensitive. Activation of RIG-I in melanoma cells continues to elicit anti-tumor effects from NK cells and CD8^+^ T cells, even under hypoxic conditions, whereas IFN-α significantly loses its CD8^+^ T cell-activating ability under hypoxia. Vitamin C-mediated clearance of hypoxia-induced ROS enhances the expression of RIG-I and further amplifies the anti-tumor effects of NK cells and CD8^+^ T cells. Interestingly, these effects depend entirely on RIG-I expression in tumor cells.^508^

##### Immune cells

RLRs also exert anti-tumor effects by directly activating intrinsic RLRs within immune cells. For instance, the activation of RIG-I on NK cells increases the surface expression of the membrane-bound TNF-related apoptosis-inducing ligand (TRAIL). This, in turn, induces death-receptor-pathway-mediated apoptosis in melanoma cells, including those negative for HLA class I. Interestingly, the activation of intrinsic RIG-I receptors within NK cells has been shown to generate a stronger tumoricidal effect than direct Type I IFN stimulation on NK cells.^496^ Moreover, the expression of LGP2 in DCs can enhance the radiotherapy efficacy for breast cancer. A deficiency of LGP2 in DCs impairs their production of Type I IFN and consequently hampers their priming capacity.^509^

Surprisingly, the activation of intrinsic RLRs in immune cells can mediate pro-tumoral effects. Specifically, the activation of intrinsic RIG-I in MDMs promotes the expression of IDO via the RIG-I/IL-6/TNF-α signaling pathway, thereby inhibiting the proliferation and function of effector T cells.^510^ Moreover, The upregulation of RIG-I in CD8^+^ T cells can inhibit the activation of STAT5, thereby suppressing the survival and cytotoxicity of CD8^+^ T cells.^504^

Research on the intrinsic role of RLRs in immune cells within the context of cancer is relatively limited. This gap in the literature highlights the need for further investigation to understand the complexities of RLR signaling in immune cells and its potential implications for cancer therapy.

#### Regulation of RLR pathway in tumor

It is important to note that the expression of RIG-I can be suppressed in tumor cells. The hypoxic environment in tumors induces HIF-1α, which downregulates the expression of RIG-I.^508^ In melanoma’s tumor-repopulating cells, a highly tumorigenic subpopulation with self-renewing capabilities, the activation of integrin β3/c-SRC/STAT3 pathway suppresses RIG-I, subsequently affecting STAT1 activation and resulting in resistance to apoptosis induced by IFN-α.^511^

The modification of the DDX58 mRNA that encodes RIG-I also participates in affecting the activation of RIG-I within tumors. The m6A demethylase ALKBH5 is highly expressed in various types of tumor cells, including HNSCC. High expression of ALKBH5 can inhibit the m6A modification of DDX58 mRNA, impair its maturation, and cause damage to the RIG-I/IFNα axis, resulting in immunosuppression, including a reduction in the number of tumor-infiltrating lymphocytes.^512^ Additionally, a suggestive study showed that the knockout of MEX3A, a protein that binds and ubiquitylates RIG-I, can increase the content of RIG-I protein in GBM and is related to tumor growth inhibition.^513^ miRNA also plays a role in regulating RLRs and influencing tumor prognosis. For instance, the 3’UTR of RIG-I is targeted by miR-545. In PDAC tissues, reduced levels of miR-545 elevate RIG-I expression, promoting tumor progression.^505^

Collectively, these findings underscore the multifaceted mechanisms that modulate RIG-I expression and activity in tumors, highlighting the potential therapeutic avenues targeting this axis for improved cancer treatment outcomes.

### Nuclear innate sensor pathway

**Fig. 5:**
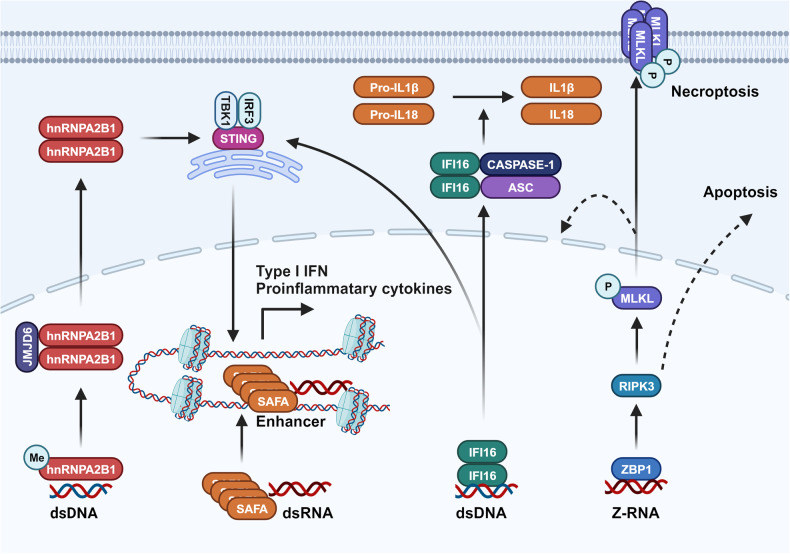
Timeline of the milestones regarding the research on the innate immune pathway. Since the proposal of the PRRs concept in 1989, the TLRs pathway, NLRs pathway, RLRs pathway, and cGAS-STING pathway have been successively discovered and associated with oncology and cancer treatment. Created with BioRender.com

In recent years, several molecules within the cell nucleus have been discovered to act as innate sensors, mediating the activation of the innate immune pathway (Fig. 5). Given that a key characteristic of tumors is genomic instability, it naturally raises the question of whether these innate sensors can detect abnormal nucleic acids in the nuclei of tumor cells, thereby activating innate immunity and inflammation, which in turn could influence tumor development. Indeed, some of these nuclear innate sensors are emerging as new potential targets in the immunotherapy of cancer.

#### Z-DNA-binding protein 1

Z-DNA binding protein 1 (ZBP1), initially discovered as a cytoplasmic DNA sensor that activates type I IFN production,^514^ has more recently been found to also act as a nuclear sensor. It detects viral-generated Z-form dsRNA (Z-RNA) and mediates RIPK3-dependent apoptosis and MLKL-dependent necroptosis.^515^ The mediation of necroptosis by ZBP1 suggests a potential role in cancer therapy, as nuclear necroptosis is significantly more immunogenic than cytoplasm-induced necroptosis due to the release of numerous DAMPs such as HMGB-1, IL-33, and DNA itself.^515^

In fact, research has found that the application of CBL0137, a small molecule that promotes the formation of Z-RNA in cells, can induce ZBP1-mediated necroptosis in tumor fibroblasts in melanoma. This drives the recruitment of CD8 + T cells to the tumor and reverses ICB resistance.^516^ Follow-up studies have shown that CBL0137 can also induce Z-DNA-mediated necroptotic cell death in liver cancer, demonstrating the potential of CBL0137 as a cancer therapeutic agent.^517^

Additionally, the ZBP1 pathway can interact with the cGAS-STING pathway. In irradiated tumor cells, the ZBP1-MLKL necroptotic cascade induces cytoplasmic accumulation of mitochondrial DNA (mtDNA), which activates the cGAS-STING pathway, thereby enhancing the type I IFN response. The MC38 mouse model of colon cancer shows that the ZBP1-MLKL necroptotic cascade is essential for the anti-tumor effect of radiotherapy.^518^

By leveraging the immunogenic properties of nuclear necroptosis and the interaction between ZBP1 and the cGAS-STING pathway, it may be possible to develop novel strategies for cancer treatment that enhance immune responses against tumors.

#### Scaffold-attachment-factor A

Scaffold-attachment-factor A (SAFA) has recently been discovered to act as a nuclear sensor for viral dsRNA. It oligomerizes and activates enhancers of antiviral genes including *IFNB1*, initiating a cytoplasm-dependent downstream antiviral immune response.^519^

The role of SAFA in tumor progression, however, remains largely unexplored. Limited studies suggest that in proliferating cells, the interaction between SAFA and the lncRNA PANDA can inhibit the transcription of pro-senescent genes, thus maintaining the cells in their current proliferative state.^520^ In Esophageal Squamous Cell Carcinoma, SAFA is significantly more expressed in tumor tissues compared to normal tissues. It can bind to PANDA to switch on the tumor proliferation program through the CyclinD1/2-Cyclin E1 and Bcl-2 pathways.^521^ However, the role of SAFA as an enhancer of immune genes in the progression of tumors has not yet been studied. This could represent an interesting direction for future research. The nuclear immune activation function of SAFA may enable it to bypass the abnormal and variable cytoplasmic pathways in tumors, potentially offering sustained efficacy in cancer therapy.

#### Heterogeneous nuclear ribonucleoprotein A2B1

Heterogeneous Nuclear Ribonucleoprotein A2B1 (hnRNPA2B1), traditionally known as an RNA-binding protein and a mediator of N6-methyladenosine(m(6)A)-dependent nuclear RNA processing events,^522^ has recently been discovered to act as a nuclear sensor of viral DNA, initiating and amplifying the innate immune response to DNA viruses.^523^ hnRNPA2B1 can recognize viral DNA and, upon dimerization and demethylation, translocate to the cytoplasm to activate TBK1-IRF3 in a STING-dependent manner, promoting the production of type I IFN. Additionally, it further facilitates m(6)A modification and translocation of cGAS–STING–related mRNAs after DNA virus infection, thereby further amplifying the immune response.^523^

The role of hnRNPA2B1 as a nuclear DNA sensor in cancer is currently in the early stages of research but has shown potential anti-tumor benefits. One study demonstrated that delivering DNA-damaging drugs to the cell nucleus could activate hnRNPA2B1, which, through a downstream cGAS-dependent pathway, promotes the production of IFN-β, mediating an anti-tumor effect. In contrast, the application of the same drug without nuclear delivery did not exhibit anti-tumor activity. Interestingly, the induced IFN-β enhanced CTLs’ PD-1 expression, showing significant benefits in combination with PD-1 inhibitors and suggesting the potential advantages of hnRNPA2B1 agonists in tumors resistant to ICB therapy.^524^

In fact, small molecule agonists of hnRNPA2B1, such as pac5, have already been developed and shown benefits in treating HBV and SARS-CoV-2 omicron infections.^525^ Applying these developments to cancer therapy is a promising direction for future exploration.

#### Interferon gamma-inducible protein 16

Interferon gamma-inducible protein 16 (IFI16) was discovered as early as 2010 to be capable of detecting viral DNA within the nucleus. It then translocates to the cytoplasm to recruit and activate the STING pathway, promoting the expression of type I IFN.^526^ It was later found that IFI16, upon sensing viral DNA, can also bind with the adapter molecule ASC and procaspase-1 to form a functional inflammasome within the nucleus.^527^

Building on the understanding of IFI16’s immune functions, researchers have discovered that its activation can also play a role in combating tumors. In HNSCC, the expression of IFI16 in tumor cells can promote apoptosis, inhibit angiogenesis, and enhance anti-tumor activity in vivo through the release of chemokines that increase macrophage recruitment.^528^ In TNBC, IFI16 has been observed to recruit damaged DNA in tumor cells, facilitating its translocation to the cytoplasm and thereby activating the STING pathway to promote an anti-tumor response.^529^ IFI16’s role in inducing inflammasome formation also contributes to tumor suppression. Experiments in hepatocellular carcinoma tissues from patients have shown that IFI16 induces the formation of inflammasomes in tumor cells, promoting cell death, an effect that can be inhibited by a caspase-1 inhibitor. Additionally, in hepatocellular carcinoma, IFI16 has been found to activate p53 to suppress tumor growth and migration.^530^

Interestingly, DNA methylation sequencing of circulating tumor cells from prostate cancer patients revealed that four IFI16-related interferon-inducible genes were epigenetically silenced through low methylation. Re-expression of IFI16 in prostate cancer cells of a mouse model relieved tumor growth and simultaneously induced anti-tumor immune activation.^531^

However, the IFI16 pathway has also been implicated in tumor drug resistance. Epigenetic regulation that downregulates the expression of IFI16 has been found to inhibit the STING-CXCL10/11 signaling pathway, which can enhance the anti-tumor effect of anti-HER2 trastuzumab.^532^ Notably, in cervical cancer, IFI16 can upregulate PD-L1 in the immune microenvironment through the STING-TBK1-NF-kB pathway, thus promoting tumor progression. This, however, also suggests the potential of IFI16 to sensitize tumors to PD-L1 inhibitors.^533^

These findings indicate that IFI16 plays a complex role in cancer, contributing to both tumor suppression and progression. Targeting the IFI16 pathway in cancer therapy offers potential avenues for enhancing immune responses against tumors and overcoming resistance to existing treatments.

#### Non-POU domain-containing octamer-binding protein

Non-POU domain-containing octamer-binding protein (NONO) is a multifunctional nuclear protein capable of binding to DNA, RNA, and proteins.^534^ Previous research has shown that NONO can retain cGAS in the cell nucleus and, during HIV infection, promotes cGAS’s DNA sensing by detecting and binding the nuclear viral capsid, thus facilitating the activation of innate immunity mediated by cGAS.^535^ The role of NONO as a nuclear sensor in cancer has been less explored.

One study demonstrated that small molecule ligands of NONO could inhibit oncogenic transcription networks.^536^ However, in TNBC, NONO can detect overexpressed moesin (MSN) in the cytoplasm of tumor cells, forming a PKC-MSN-NONO complex that translocates to the nucleus, thereby activating CREB signaling and promoting tumor progression. Targeting inhibition of NONO can suppress tumor progression.^537^ Therefore, further research is needed to understand the types of molecules sensed by NONO in cancer and its consequent roles.

The ability of nuclear sensors to recognize RNA and DNA, and subsequently activate downstream IFNs and pro-inflammatory molecules, demonstrates their potential in anti-tumor activity. This is particularly relevant given that the genomic instability inherent in many cancers naturally provides the nucleic acid substrates that activate these sensors. However, a key focus of future research is how to promote the activation of these pathways. Developing delivery tools to specifically target and transport relevant drugs directly to the cell nucleus is a promising direction.

## Cancer therapeutic strategies targeting innate immune pathway

The previous discussion has highlighted the potential of targeting the innate immune pathway to improve cancer prognosis. Innate immune pathway receptor agonists have exhibited robust anticancer efficacy in in vitro and in vivo animal studies across various malignancies (Table 1). In this section, we will explore the evidence from clinical trials for activating innate immunity to enhance patient outcomes, as well as combination treatment strategies adopted to better unleash the immunostimulatory role of the innate immune pathway.

### Therapeutic efficacy: evidence from clinical trials

Numerous agonists targeting the innate immune pathway have been developed (Table 2.), some of which are currently undergoing clinical trials and have shown translational potential.

**Table 2:** Agonists of innate immune pathway receptors

Drugs	Targets	Type	Route of administration	Regulatory status and indications	Illustration	Refs.
ADU-S100 (MIW815 FMW9ZVF53N1 1638750-95-4)	STING	Small molecule	Intratumoral	Approved for clinical trials in advanced/metastatic solid tumors or lymphomas	Synthetic CDN compounds	^251,252^ NCT02675439 NCT03937141 NCT03172936;
MK-1454 (Ulevostinag)	STING	Small molecule	Intratumoral	Approved for clinical trials in lymphoma and solid tumors	Synthetic CDN compounds	NCT04220866 NCT03249792
SB 11285	STING	Small molecule	Intravenous	Approved for clinical trials in advanced solid tumors	Synthetic CDN compounds	NCT04096638
BMS-986301	STING	Small molecule	Intravenous Intramuscular	Approved for clinical trials in advanced solid tumors	Synthetic CDN compounds	NCT03956680
BI STING (BI 1387446)	STING	Small molecule	Intratumoral	Approved for clinical trials in advanced or metastatic solid tumors	Synthetic CDN compounds	NCT04147234
IMSA101	STING	Small molecule	Intratumoral	Approved for clinical trials in NSCLC, RCC, oligoprogressive solid tumor and refractory malignancies	Synthetic CDN compounds	NCT05846646 NCT05846659 NCT04020185 NCT06026254
MK-2118	STING	Small molecule	Intratumoral Subcutaneous	Approved for clinical trials	Synthetic Non-CDN Small Molecule	NCT03249792
GSK3745417	STING	Small molecule	Intravenous	Approved for clinical trials in advanced solid tumors	Synthetic Non-CDN Small Molecule	NCT03843359
TAK-676 (Dazostinag disodium)	STING	Small molecule	Intravenous	Approved for clinical trials in advanced or metastatic solid tumors	Synthetic Non-CDN Small Molecule	NCT04879849 NCT04420884
E7766	STING	Small molecule	Intratumoral	Approved for clinical trials in advanced solid tumors and lymphoma	Synthetic Non-CDN Small Molecule. Compared to traditional STING agonists, MBSA E7766 offers a more stable and binding affinity to STING due to its rigid macrocycle bridge. This increases its effectiveness in enhancing the immune response.	NCT04144140
SNX281	STING	Small molecule	Intravenous	Approved for clinical trials in advanced solid tumors and lymphoma	Synthetic Non-CDN Small Molecule	NCT04609579
KL340399	STING	Small molecule	Intratumoral	Approved for clinical trials in advanced solid tumors	Synthetic Non-CDN Small Molecule	NCT05549804 NCT05387928
CRD5500	STING	Small molecule	Intravenous Subcutaneous	Proven efficacy in CT26 syngeneic murine model	Synthetic Non-CDN Small Molecule. CRD5500 can Activate all five common human STING variants.	^681^
CDK-002	STING	Exosomes	Intratumoral	Approved for clinical trials in advanced/metastatic, recurrent, injectable solid tumors	CDK002 exosomes carry a STING agonist and CD315 glycoprotein. When administered intratumorally, they selectively bind to STING on antigen-presenting cells in the TME, triggering the STING pathway. This targeted approach avoids off-target toxicity and enhances the systemic anti-tumor immune response mediated by tumor-resident APCs.	NCT04592484
XMT-2056	STING	Antibody-drug conjugates	Intravenous	Approved for clinical trials in advanced/recurrent solid tumors that express HER2	Antibody-drug conjugates consisting of HT-19, a monoclonal antibody targeting the tumor-associated antigen HER2, attached to a payload containing STING agonist.	NCT05514717
SYNB1891	STING	Bacterial Vectors	Intratumoral	Approved for clinical trials in metastatic solid neoplasm lymphoma	SYNB1891 is a genetically modified version of the probiotic Escherichia coli Nissle 1917 (EcN). It produces cyclic dinucleotides when oxygen levels are low.	^538^ NCT04167137
ONM-500 nanovaccine	STING	Novel pH-sensitive polymer	Subcutaneous	Proven efficacy in TC-1 cervical cancer murine model	A new polymer acts as a carrier for delivering antigens to DCs while also functioning as an adjuvant by activating the STING pathway. This pH-sensitive polymer forms antigen-encapsulating nanoparticles.	^682^
STING-NPs (STING-activating nanoparticles)	STING	Nanoparticles	Intratumoural	Proven efficacy in B16.F10 melanoma murine model and freshly isolated human melanoma tissue	STING-NPs are designed to enhance cytosolic delivery of the endogenous CDN ligand for STING	^683^
Bacillus Calmette-Guerin (BCG)	TLR2 TLR4	Attenuated vaccine	Intratumoural (intravesical) Intramuscular	Approved for the treatment of bladder cancer	BCG is a weakened strain of Mycobacterium bovis, used as a vaccine for tuberculosis. It is a live, attenuated version closely related to M. tuberculosis.	^541^
Pam3CSK4	TLR1/2	Small molecule	Intratumoural	Approved for clinical trials (Non-oncologic treatment); Proven efficacy in ALL in vitro	Synthetic analog of the triacylated N-terminal part of bacterial lipoproteins	^684^ NCT01143480
XS15 (Pam3Cys-GDPKHPKSF)	TLR1/2	Small molecule	Subcutaneous	Approved for clinical trials in cancers, including GBM	Synthetic analog of the triacylated N-terminal part of bacterial lipoproteins	NCT04842513 NCT05014607
Pam2CSK4	TLR2/6	Small molecule	Intratumoural Inhalation	Approved for clinical trials (Non-oncologic treatment)	Synthetic analog of diacylated lipopeptide	^685^ NCT02124278 NCT04312997
Poly I:C	TLR3 RIG-I MDA5	dsRNA	Intratumoral Intramuscular Intravenous Subcutaneous	Approved for clinical trials in various cancers, including gliomas	A synthetic polyinosinic-polycytidylic acid double-stranded RNA	^307^
Poly ICLC	TLR3 RIG-I MDA5	dsRNA complex	Intratumoral Intramuscular Intravenous Subcutaneous	Approved for clinical trials in various cancers, including gliomas	A synthetic complex of carboxymethylcellulose, polyinosinic-polycytidylic acid, and poly-L-lysine double-stranded RNA. More resistant to RNase degradation and thus more stable than Poly I:C	^307^ NCT00374049
YS-ON-001	TLR3 RIG-I MDA5	Inactivated vaccine	Intramuscular	Approved for clinical trials in advanced solid tumors	YS-ON-001 is a combination of an inactivated purified rabies virus (IPRV) and Poly I:C IPRV can activate the immune system by promoting Th1 cells, DCs, B cells, NK cells, and M1 macrophage polarization. It also decreases Tregs and increases anti-tumor cytokine production.	NCT03131765
BO-112	TLR3 RIG-I MDA5	Nanoparticles	Intravenous	Approved for clinical trials in resectable soft tissue sarcoma	A nanoplexed form of poly (I:C) that aims to mimic viral particles loaded with dsRNA of viral features	^686^ NCT04420975
Rintatolimod (Ampligen)	TLR3	dsRNA	Intravenous	Approved for clinical trials in metastatic PDAC	A mismatched, dsRNA molecule. Does not activate MDA5 and RIG-I; Development for the treatment of a variety of chronic diseases and viral disorders, such as chronic fatigue syndrome	^687^ NCT05927142 NCT00215813
G100 (GLA-SE)	TLR4	Lipid A derivative	Intratumoural	Approved for clinical trials in various cancers, including sarcoma, lymphoma	A synthetic lipid A derivative composed of glucopyranosyl lipid-A (GLA) in a stable, oil-in-water emulsion (GLA-SE)	NCT02387125 NCT02501473
GSK1795091	TLR4	Lipid A derivative	Intravenous	Approved for clinical trials in cancers	A synthetic analog of bacterial lipid A. Side effects include chills, fatigue, pyrexia, nausea, and vomiting.	NCT02798978 NCT03447314
Monophosphoryl lipid A (MPL)	TLR4	Monophosphoryl lipid A	Intramuscular	Approved for the usage as adjuvants in some vaccines	a TLR4 agonist derived from the cell wall of nonpathogenic Salmonella, commonly used as a nontoxic, FDA-approved adjuvant in viral vaccines	^688^ NCT01584115 NCT02038907
HSPPC-96	TLR2 TLR4	Polypeptide vaccine	Intradermal	Approved for clinical trials in various cancers, including gliomas	HSPPC-96 is a protein peptide complex consisting of a 96 kDa heat shock protein (Hsp), gp96, and associated cellular peptides. It interacts with cell surface receptors on APCs, including CD36/CD91/CD40/CD14/TLR2/TLR4 leading to activation of the NF-κB pathway.	^689,690^ NCT03018288 NCT02722512 NCT01814813
M-VM3 (Mobilan)	TLR5	Engineered adenovirus	Intratumoural	Approved for clinical trials in prostate cancer	A nanoparticle-based formulation with a non-replicating adenovirus carries TLR5 and its ligand protein 502 S. This selectively targets cells with the Coxsackievirus and adenovirus receptor (CAR), commonly found in certain human tumors. TLR5 and 502 S combined activate ongoing TLR5 signaling.	NCT02654938 NCT02844699
Entolimod (CBLB502)	TLR5	Polypeptide	Intramuscular Subcutaneous	Approved for clinical trials in advanced or metastatic solid tumors	Entolimod is a polypeptide from Salmonella’s flagellin protein and may inhibit the growth of TLR5-expressing tumor cells in a radiation-independent manner	NCT01527136 NCT02715882 NCT01728480
Imiquimod (R837 Aldara)	TLR7	Small Molecule	Topical	Approved for the treatment of superficial basal cell carcinoma and certain skin conditions, including actinic keratosis, External genital warts and perianal warts	It is a cream for topical use only	^691^ NCT00821964 NCT00504023
852 A (CPG-52852)	TLR7	Small Molecule	Intravenous	Approved for clinical trials in various cancers, including melanoma, neoplasms, breast cancer, ovarian cancer, and cervical cancer	A synthetic imidazoquinoline TLR7 agonist. Grade 3–4 toxicities included nausea, dyspnea, fever, myalgia, malaise, and cough.	NCT00821964
MBS8	TLR7	Nanoparticles	Intravenous	Approved for clinical trials in advanced solid tumor	A micelle nanoparticle formulation composed of TLR7 agonist phospholipid conjugate 1v270 encapsulated within 1,2-Dioleoyl-sn-glycero-3-phosphoethanolamine (DOPE)-polyethylene glycol (PEG)-2000, with potential immunostimulating activity. The micelle formulation was engineered to reduce systemic cytokine production which may lower systemic toxicity.	NCT04855435
NKTR-262	TLR7/8	Polymer-modified molecule	Intratumoural	Approved for clinical trials in advanced or metastatic solid Tumors	A formulation composed of an agonist of TLR7/8 that is attached to polyethylene glycol (PEG) via a hydrolyzable glycine linker. Pegylation of the TLR7/8 agonist improves its retention in the TME. It also allows for localized and sustained release, resulting in increased effectiveness and reduced systemic exposure compared to the naked TLR7/8 agonist.	NCT03435640
Resiquimod (R848)	TLR7/8	Small molecule	Topical	Approved for clinical trials in skin cancer and other skin disease as well as some other cancers, including glioma, melanoma, lymphoma	A type of imidazoquinoline and a type of immunomodulator. It has been reported to add to a tumor vaccine to improve the antitumor immune response.	NCT01676831 NCT01808950 NCT01204684
TransCon TLR7/8 Agonist	TLR7/8	Hydrogel carrier-based formulation	Intratumoral	Approved for clinical trials in advanced or metastatic solid Tumors	A hydrogel carrier-based, sustained intra-tumoral release formulation of resiquimod, a TLR7/8 agonist and an imidazoquinolinamine	NCT04799054
ANA773 tosylate	TLR7/8	Small molecule	Oral	Investigational Approved for clinical trials in HCV Infection	The tosylate salt form of ANA773, a TLR7 agonist prodrug with potential immunostimulating activity. Upon oral administration, ANA773 is metabolized into its active form that binds to and activates TLR7.	NCT01211626
APR003	TLR7	Small molecule	Oral	Approved for clinical trials in advanced colorectal carcinoma	Upon oral administration, APR003 concentrates in the GI tract and liver. This localized distribution enhances local immune responses and tumor elimination, while minimizing potential side effects throughout the body.	NCT04645797
BNT411	TLR7	Small molecule	Intravenous	Approved for clinical trials in extensive-stage small cell lung cancer	Enhanced responses when combined with cytotoxic treatments and immune checkpoint inhibitors	NCT04101357
CAN1012	TLR7	Small molecule	Intratumoral	Approved for clinical trials in solid tumor and cancer metastatic	A TLR7 agonist with potential immunostimulating and antitumor activities.	NCT05580991
AL 034 (JNJ-64794964 TQA 3334)	TLR7	Small molecule	Oral	Approved for clinical trials in hepatitis B	An orally bioavailable TLR7 agonist	NCT03285620
LHC165	TLR7	Small molecule	Intratumoral	Approved for clinical trials in solid tumor	A benzonapthyridine TLR7 agonist that is adsorbed to aluminum hydroxide	NCT03301896
RO7119929	TLR7	Small molecule	Oral	Approved for clinical trials in unresectable advanced or metastatic hepatocellular carcinoma, biliary tract cancer, or solid tumors with hepatic metastases	An orally bioavailable TLR7 agonist	NCT04338685
SHR2150	TLR7	Small molecule	Oral	Approved for clinical trials in solid tumor	An orally bioavailable TLR7 agonist	NCT05141422 NCT04588324 NCT04802811
MEDI9197	TLR7/8	Small molecule	Intratumoral	Approved for clinical trials in HIV infection and solid tumor	A novel TLR7/8 agonist	NCT02556463 NCT05828095
DSP-0509	TLR7	Small molecule	Intravenous	Approved for clinical trials in advanced solid tumors	A synthetic, small molecule, TLR7 agonist	NCT03416335
SBT6050	TLR8	Antibody-drug conjugate	Intravenous	Approved for clinical trials in HER2-positive solid tumors	An immunotherapeutic composed of a monoclonal antibody directed against the tumor-associated antigen human epidermal growth factor receptor 2 (EGFR2; HER2; ErbB2) conjugated to a TLR8 agonist. The anti-HER2 monoclonal antibody specifically targets and attaches to HER2 found on tumor cells, effectively localizing the TLR8 agonist directly to the tumor site.	NCT04460456 NCT05091528
Motolimod (VTX-2337 VTX-378)	TLR8	Small molecule	Intratumoral	Approved for clinical trials in HNSCC, HER2-positive solid tumors	A synthetic, small molecule, TLR8 agonist	NCT03906526 NCT04272333 NCT02650635 NCT02431559 NCT02124850
SBT6290	TLR8	Antibody-drug conjugate	Intravenous	Approved for clinical trials in HNSCC, HER2-positive solid tumors	An immunotherapeutic treatment comprising a monoclonal antibody that targets the cell surface adhesion molecule and tumor-associated antigen nectin-4 (PVRL4), conjugated with a TLR8 agonist. Upon administration of SBT6290, the nectin-4-directed TLR8 agonist monoclonal antibody specifically binds to nectin-4 on tumor cells, delivering the TLR8 agonist directly to the tumor site. Consequently, the TLR8 agonist molecule binds to TLR8 receptors on myeloid cells within the TME	NCT05234606
DN-A1 (DN1508052-01)	TLR8	Small molecule	Subcutaneous	Approved for clinical trials in advanced solid tumors	A synthetic, small molecule, TLR8 agonist	NCT03934359
Cavrotolimod (AST 008)	TLR9	Nanoparticle	Intratumoral	Approved for clinical trials in advanced solid tumors	A spherical nucleic (SN) acid consists of densely arranged nucleic acids (DNA) on the surface of liposomal nanoparticles, forming a compact 3D structure. This unique design allows for efficient cellular uptake and enhanced presentation of the DNA to stimulate TLR9. Furthermore, SN confers protection against degradation by nucleases and prolongs the construct’s half-life compared to linear oligonucleotides lacking the SN acid format.	NCT03684785
CpG-ODN	TLR9	CpG-ODNs	intratumor	Approved for clinical trials in advanced solid tumors	CpG oligodeoxynucleotides (CpG ODNs) are short single strands of synthetic DNA that contain a cytosine triphosphate deoxynucleotide (‘C’) linked through a phosphodiester (‘p’) to a guanine triphosphate deoxynucleotide (‘G’). the most common toxicities were lymphopenia, mild fever, seizures, and transient neurological worsening.	^692^ NCT04952272 NCT00190424
DV281	TLR9	CpG-ODNs	Inhaled	Approved for clinical trials in advanced NSCLC	A proprietary synthetic, aerosolized C-class CpG ODNs agonist of TLR9	NCT03326752
Tilsotolimod sodium (IMO 2125)	TLR9	CpG-ODNs	Intradermal	Approved for clinical trials in solid tumors, including melanoma	A proprietary synthetic oligonucleotide-based agonist of TLR9	NCT04126876 NCT03052205 NCT03445533 NCT03865082
DUK-CPG-001	TLR9	CpG-ODNs	Intravenous	Approved for clinical trials in myeloid and lymphoid malignancies	A synthetic CpG-rich oligonucleotide-based agonist of TLR9	NCT02452697 NCT02115126
EMD 1201081 (IMO-2055)	TLR9	CpG-ODNs	Subcutaneous	Approved for clinical trials in HNSCC, NSCLC, RCC as well as colorectal cancer	A synthetic oligonucleotide containing phosphorothioate, unmethylated CpG containing oligodeoxynucleotide	NCT01040832 NCT01360827 NCT00719199 NCT00633529 NCT00729053
MGN1703 (lefitolimod)	TLR9	CpG-ODNs	Subcutaneous	Approved for clinical trials in melanoma, SCLC as well as colorectal cancer	A synthetic oligonucleotide based on a proprietary double stem-loop immunomodulator design with potential immunostimulating activity. Side effects include fatigue and activated partial thromboplastin time prolongation.	NCT02668770 NCT02200081 NCT01208194 NCT02077868 NCT03837756
SD-101	TLR9	CpG-ODNs	Intratumoral	Approved for clinical trials in various cancers, including pancreatic carcinoma, liver cancer, melanoma	A proprietary oligonucleotide with immunostimulatory activity. Furthermore, this agent does not cause a generalized activation of the immune system. The most common adverse events related to SD-101 were injection-site reactions and transient, mild-to-moderate “flu-like” symptoms.	NCT05607953 NCT05220722 NCT04935229 NCT03410901
Vidutolimod (ARB-1598 CMP-001 CYT-003 QbG10)	TLR9	Virus-like particle	Intratumoral	Approved for clinical trials in advanced cancer or metastatic cancer, especially melanoma	A virus-like particle (VLP) composed of the Qbeta bacteriophage capsid encapsulating the TLR9 agonist G10, an unmethylated CpG-A oligodeoxynucleotide (ODN), with potential immunostimulating and antineoplastic activities. Upon administration, the VLPs are preferentially assimilated by APCs, facilitating intracellular TLR9 activation via the delivered oligonucleotide. Vidutolimod inducing a distinct pattern of pDC differentiation, possess a stronger immune stimulating ability than traditional TLR9 agonists.	NCT04916002 NCT04401995 NCT05445609 NCT04695977 NCT02680184
CpG-STAT3 siRNA CAS3/SS3	TLR9	Small molecule	Intratumoral	Approved for clinical trials in Lymphoma	A conjugate composed of a TLR9 agonist and a siRNA targeting STAT3. This conjugate, which combines a CpG-ODN with siRNA directed against STAT3, holds the potential for both immunostimulation and anti-cancer activities.	NCT04995536
EG-70	RIG-I	Nanoparticle	Intratumoral (intravesical)	Approved for clinical trials in superficial bladder cancer	A nanoparticle-based formulation composed of a non-viral plasmid DNA vector encoding the human pro-inflammatory cytokine IL-12 and a RIG-I activating moiety encapsulated in dually derivatized chitosan (DDX) nanoparticles.	NCT04752722
RGT100 (MK-4621)	RIG-I	RNA oligonucleotide	Intratumoral	Approved for clinical trials in advanced solid tumors	A synthetic RNA oligonucleotide and selective agonist of RIG-I	NCT03065023
Mifamurtide (Muramyltripeptide; L-MTP-PE)	NOD2	Small molecule	Intravenous	Approved. Approved for the treatment of osteosarcoma	Mifamurtide is a synthetic derivative of muramyl dipeptide (MDP), a bacterial wall motif that activates immune cells like macrophages and monocytes through recognition by NLRs and TLRs, especially NOD2. This recognition triggers proinflammatory cytokine production, contributing to antibacterial and anticancer effects	^570^ NCT00631631
BMS-986299	NLRP3	Small molecule	Intratumoral	Approved for clinical trials in advanced solid tumors	A synthetic NPRP3 agonist	NCT03444753

*ALL* acute lymphoblastic leukemia, *CDNs* cyclic dinucleotides, *HNSCC* head and neck squamous cell carcinoma, *TME* tumor microenvironment

#### cGAS-STING agonists

Clinical research on the cGAS-STING pathway is still in its infancy, but the limited evidence available suggests the efficacy and safety of targeting this pathway for stimulation. A study that administered intra-tumoral injections of the STING agonist MIW815 (ADU-S100) to patients with advanced/metastatic solid tumors or lymphomas revealed that MIW815 was well tolerated in these patients. In 94% of assessable injected lesions, there was either stabilization or reduction in lesion size. Moreover, an increase in inflammatory cytokines and peripheral blood T cell clonal expansion indicated systemic immune activation (NCT02675439).^251^

Another clinical study developed an Engineered E. coli Nissle Strain called SYNB1891 that produces cyclic dinucleotides under hypoxic conditions. By injecting SYNB1891 into tumors of patients with refractory advanced cancers, the intention was to activate the STING pathway. Phase I clinical trials showed that patients treated with SYNB1891 did not experience any serious adverse events related to SYNB1891, nor were any infections associated with SYNB1891 observed. Furthermore, upregulation of ISGs, chemokines/cytokines, and T cell response genes were observed in core biopsy tissues. Additionally, there was a dose-related increase in serum cytokines, and stable disease was observed in four participants who were previously resistant to PD-1/L1 antibodies (NCT04167137).^538^

Moreover, there are currently over a dozen STING agonists in clinical trials (Table 3), and the outcomes of these trials are eagerly anticipated.

**Table 3:** Ongoing clinical trials involving innate immune pathway receptor agonists

NCT Code	Drugs	Targets	Conditions or diseases	Combined therapy	Phase	Masking	Allocation	Estimated/actual enrollment	Status
NCT04096638	SB 11285	STING	Advanced solid tumors	Atezolizumab	I	Open label	Non-randomized	146	Recruiting
NCT03956680	BMS-986301	STING	Advanced solid tumors	Nivolumab + ipilimumab	I	Open label	N/A	190	Active, not recruiting
NCT04147234	BI 1387446	STING	Advanced or metastatic cancer (solid tumors)	Ezabenlimab	I	Open label	Non-randomized	39	Active, not recruiting
NCT04020185	IMSA101	STING	Refractory malignancies	ICI/IO therapy	I & II	Open label	Non-randomized	115	Recruiting
NCT05846646	IMSA101	STING	Oligometastatic NSCLC and RCC	PULSAR + ICI	II	Open label	Randomized	46	Recruiting
NCT05846659	IMSA101	STING	Oligoprogressive solid tumor malignancies	PULSAR + ICI	II	Open label	Randomized	51	Recruiting
NCT03843359	GSK3745417	STING	Advanced solid tumors	Dostarlimab	I	Open label	Non-randomized	97	Active, not recruiting
NCT04879849	TAK-676	STING	NSCLC; TNBC; SCCHN	Pembrolizumab + image-guided radiation therapy	I	Open label	N/A	65	Recruiting
NCT04420884	TAK-676	STING	Advanced or metastatic solid tumors	Pembrolizumab + platinum + 5-fluorouracil	I	Open label	Non-randomized	288	Recruiting
NCT04609579	SNX281	STING	Advanced solid tumors and lymphoma	Pembrolizumab	I	Open label	Non-randomized	134	Recruiting
NCT05549804	KL340399	STING	Advanced solid tumors	None	I	Open label	Non-randomized	30	Recruiting
NCT05387928	KL340399	STING	Advanced solid tumors	None	I	Open label	Non-randomized	30	Not yet recruiting
NCT05514717	XMT-2056	STING	Advanced/recurrent solid tumors that express HER2	None	I	Open label	N/A	171	Suspended (Clinical Hold by the FDA)
NCT04167137	SYNB1891	STING	Metastatic solid tumors and lymphoma	Atezolizumab	I	Open label	Non-randomized	70	Unknown
NCT04842513	XS15	TLR1/2	Newly diagnosed HLA-A2-positive MGMT-methylated GBM	Multi peptide vaccine + radiation +tem ozolomide	I	Open label	N/A	15	Recruiting
NCT05937295	XS15	TLR1/2	Fibrolamellar HCC or other cancer entities carrying the DNAJB1-PRKACA fusion transcript	Fusion-VAC (DNAJB1-PRKACA fusion transcript-based peptide vaccine) + Atezolizumab	I	Open label	N/A	20	Not yet recruiting
NCT05014607	XS15	TLR1/2	Advanced solid and hematological malignancies without any approved treatment options.	Personalized multi-peptide vaccine	N/A	N/A	N/A	N/A	Available
NCT04688385	XS15	TLR1/2	Chronic lymphocytic leukemia patients undergoing Ibrutinib-based Regimes	Personalized multi-peptide vaccination	I	Open label	N/A	20	Recruiting
NCT03732547	Poly I:C	TLR3 RIG-I MDA5	Unresectable HCC	PD-1 mAb	II	Open label	N/A	60	Recruiting
NCT03392545	Poly I:C	TLR3 RIG-I MDA5	Malignant gliomas	Radiation + GM-CSF	I	Open label	N/A	30	Unknown
NCT04420975	BO-112	TLR3 RIG-I MDA5	Resectable soft tissue sarcoma	Nivolumab	I	Open label	N/A	14	Active, not recruiting
NCT05927142	Rintatolimod	TLR3	Metastatic PDAC	Durvalumab	I & II	Open label	N/A	43	Not yet recruiting
NCT05494697	Rintatolimod	TLR3	Locally advanced PDAC	None	II	Open label	Randomized	90	Recruiting
NCT01720836	Poly ICLC	TLR3 RIG-I MDA5	NSCLC	MUC1 (Mucin1) peptide vaccine	I & II	Open label	Non-randomized	30	Recruiting
NCT05254184	Poly ICLC	TLR3 RIG-I MDA5	NSCLC	Mutant-KRAS peptide vaccine + Nivolumab + ipilimumab	I	Open label	N/A	12	Recruiting
NCT05098210	Poly ICLC	TLR3 RIG-I MDA5	Stage IIIC-IV melanoma or Her2 negative metastatic refractory breast cancer	Personalized neo-antigen peptide vaccine	I	Open label	N/A	20	Recruiting
NCT03606967	Poly ICLC	TLR3 RIG-I MDA5	Metastatic TNBC	Personalized synthetic long peptide vaccine + Nab-paclitaxel + durvalumab +tremelimumab +carboplatin + gemcitabine hydroch loride	II	Open label	Randomized	70	Recruiting
NCT04616248	Poly ICLC	TLR3 RIG-I MDA5	Unresectable and metastatic solid tumors with injectable palpable disease	CDX-1140 (CD40 agonistic mAb) + CDX-301 (recombinant Flt3 ligand) + Radiation therapy	I	Open label	Non-randomized	18	Recruiting
NCT03206047	Poly ICLC	TLR3 RIG-I MDA5	Recurrent ovarian, fallopian tube, or primary peritoneal cancer	DEC-205/NY-ESO-1 fusion protein CDX-1401 vaccine + atezolizumab +guadec itabine	I & II	Open label	Randomized	75	Active, not recruiting
NCT04201873	Poly ICLC	TLR3 RIG-I MDA5	Surgically accessible recurrent GBM	Dendritic cell tumor cell lysate vaccine (ATL-DC vaccine) + pembrolizumab	I	Double (Participant, Investigator)	Randomized	40	Recruiting
NCT02549833	Poly ICLC	TLR3 RIG-I MDA5	WHO grade II glioma	GBM6-AD lysate protein vaccine	I	Open label	Randomized	28	Active, not recruiting
NCT03665545	Poly ICLC	TLR3 RIG-I MDA5	Relapsing GBM	Multipeptide vaccine IMA950 + pembrolizumab	I & II	Open label	Randomized	18	Active, not recruiting
NCT05557240	Poly ICLC	TLR3 RIG-I MDA5	Newly diagnosed GBM	NeoPep vaccine	N/A	Open label	N/A	10	Recruiting
NCT05457959	Poly ICLC	TLR3 RIG-I MDA5	Recurrent and/or progressive diffuse hemispheric glioma, H3 G34-mutant	Dendritic cell tumor peptide vaccine + ipilimumab +niv olumab	I	Single (Participant)	Randomized	15	Not yet recruiting
NCT03223103	Poly ICLC	TLR3 RIG-I MDA5	GBM	Tumor treating fields + Peptides	I	Open label	N/A	13	Active, not recruiting
NCT03131765	YS-ON-001	TLR3 RIG-I MDA5	Advanced solid tumors	None	I	Open label	N/A	41	Recruiting
NCT02406781	G100	TLR4	Advanced sarcomas	Pembrolizumab (MK3475) + metronomic cyclophosphamide	II	Open label	Non-randomized	227	Unknown
NCT03650257	HSPPC-96	TLR2 TLR4	GBM	Temozolomide + radiation	II	Open label	Randomized	150	Recruiting
NCT01204684	Resiquimod	TLR7/8	Glioma Anaplastic astrocytomaanaplastic astro-oligoden droglioma GBM	Tumor lysate-pulsed DC vaccination	II	Open label	Randomized	60	Active, not recruiting
NCT02126579	Resiquimod	TLR7/8	Melanoma	Peptide vaccine (LPV7) + tetanus peptide + Poly ICLC + incomplete Freund’s adjuvant	I & II	Open label	Randomized	62	Unknown
NCT05710848	Resiquimod (STM-416)	TLR7/8	Non-muscle-invasive bladder cancer	None	I & II	Open label	N/A	75	Recruiting
NCT04799054	TransCon TLR7/8 Agonist	TLR7/8	Advanced or metastatic solid tumors	Pembrolizumab	I & II	Open label	Non-randomized	220	Recruiting
NCT04859361	Imiquimod	TLR7	Cervical intraepithelial neoplasia	None	N/A	Single (Investigator)	Randomized	104	Active, not recruiting
NCT03196180	Imiquimod	TLR7	High-grade cervical intraepithelial neoplasia	Fluorouracil	Early I	Open label	N/A	13	Active, not recruiting
NCT04883645	Imiquimod	TLR7	Oral cancer	None	Early I	Open label	N/A	18	Recruiting
NCT03370406	Imiquimod	TLR7	squamous cell carcinoma	5-Fluorouracil	I	Open label	Randomized	30	Recruiting
NCT03276832	Imiquimod	TLR7	Stage IIIB-IV melanoma	Pembrolizumab	Early I	Open label	N/A	7	Active, not recruiting
NCT02059499	Imiquimod	TLR7	HIV-positive patients with high-grade anal squamous skin lesions	Fluorouracil	III	Single (Outcomes Assessor)	Randomized	91	Active, not recruiting
NCT03534947	Imiquimod	TLR7	Basal cell carcinoma	Sonidegib	II	Open label	Non-randomized	10	Recruiting
NCT00799110	Imiquimod	TLR7	Ovarian cancerprimary peritoneal cancerfallopian tube cancer	Dendritic cell/tumor fusion vaccine + GM-CSF	II	Open label	Randomized	23	Active, not recruiting
NCT01795313	Imiquimod	TLR7	Recurrent ependymomas	vaccination with HLA-A2 restricted peptides	I	Open label	N/A	24	Recruiting
NCT01803152	Imiquimod	TLR7	Sarcoma	Dendritic cells vaccine +gemcitabine	I	Open label	Non-randomized	19	Active, not recruiting
NCT05055050	Imiquimod (UGN-201)	TLR7	Bladder cancer	None	I	Open label	N/A	10	Recruiting
NCT02600949	Imiquimod	TLR7	Advanced pancreatic cancer or colorectal cancer	Synthetic tumor-associated peptide vaccine +pembrolizumab +sotigalimab (CD40 agonistic mAb)	I	Open label	Non-randomized	150	Recruiting
NCT03872947	Imiquimod	TLR7	Advanced solid tumors	TRK-950 + irinotecan+ leucovorin+ 5-FU+ gemcitabine+ cisplatin+ carboplatin+ ramucirumab + paclitaxel+ nivolumab+ pembrolizumab+ bevacizumab+ topotecan+ PLD	I	Open label	Non-randomized	169	Recruiting
NCT02135419	Imiquimod	TLR7	Patients with HIV and anal high-grade lesions	Fluorouracil + infrared photocoagulation therapy + thermal ablation therapy + laser therapy	III	Open label	Randomized	4446	Active, not recruiting
NCT05375903	Imiquimod (UGN-201)	TLR7	Recurrent non-muscle invasive bladder cancer	Zalifrelimab (UGN-301) + gemcitabine	I	Open label	Non-randomized	60	Recruiting
NCT05641545	Imiquimod	TLR7	Advanced renal cell carcinoma	Individual peptide vaccination + GM-CSF	I	Open label	N/A	10	Recruiting
NCT04808245	Imiquimod	TLR7	Newly diagnosed H3-mutated glioma	Tecentriq + H3K27M peptide vaccine	I	Open label	N/A	15	Recruiting
NCT04642937	Imiquimod	TLR7	Recurrent GBM	hP1A8 (a new adjuvant CD200 activation receptor ligand) + GBM6-AD vaccine	I	Open label	N/A	24	Active, not recruiting
NCT03893903	Imiquimod	TLR7	Progressive diffuse glioma	IDH1R132H peptide vaccine + avelumab	I	Open label	Randomized	60	Recruiting
NCT04855435	MBS8	TLR7	Advanced solid tumor	None	I	Open label	N/A	69	Recruiting
NCT04101357	BNT411	TLR7	Solid tumorextensive-stage small cell lung cancer	Atezolizumab + carboplatin +et oposide	I & II	Open label	Non-randomized	60	Recruiting
NCT05580991	CAN1012	TLR7	Solid tumors	None	I	Open label	N/A	96	Recruiting
NCT04987112	CAN1012	TLR7	Solid tumorcancer met astatic	None	I	Open label	N/A	36	Recruiting
NCT04460456	SBT6050	TLR8	HER2 positive solid tumors	Pembrolizumab + cemiplimab	I	Open label	Randomized	58	Active, not recruiting
NCT04126876	IMO-2125	TLR9	Pathological tumor stage (p) T3-4 cN0M0 melanoma	None	II	Triple (Participant, Care Provider, Investigator)	Randomized	214	Recruiting
NCT02452697	DUK-CPG-001	TLR9	Myeloid malignancieslymphoid malignancies	NK cell-enriched donor lymphocyte infusions	II	Open label	Randomized	100	Active, not recruiting
NCT02668770	MGN1703	TLR9	Advanced solid malignancies	Ipilimumab	I	Open label	Non-randomized	28	Active, not recruiting
NCT05607953	SD-101	TLR9	Locally advanced PDAC	Pembrolizumab	I	Open label	N/A	60	Recruiting
NCT05220722	SD-101	TLR9	Primary liver tumors	Pembrolizumab + nivolumab +ipili mumab	I & II	Open label	N/A	89	Recruiting
NCT04935229	SD-101	TLR9	Metastatic uveal melanoma in the liver	Nivolumab + relatlimab +ipilimumab	I	Open label	N/A	60	Recruiting
NCT03831295	SD-101	TLR9	Metastatic solid malignancies	Anti-OX40 antibody BMS 986178	I	Open label	N/A	12	Recruiting
NCT03410901	SD-101	TLR9	Low-grade B-cell non-Hodgkin lymphomas	Anti-OX40 antibody BMS 986178 + radiation therapy	I	Open label	N/A	15	Active, not recruiting
NCT02927964	SD-101	TLR9	Relapsed or refractory grade 1–3 A follicular lymphoma	Ibrutinib + radiation therapy	I & II	Open label	N/A	30	Active, not recruiting
NCT03007732	SD-101	TLR9	Hormone-naïve oligometastatic prostate cancer	Pembrolizumab + leuprolide acetate +abiraterone acetate +prednisone +stereotactic body radiation therapy	II	Open label	Randomized	23	Active, not recruiting
NCT01042379	SD-101	TLR9	Breast cancer	Pembrolizumab	II	Open label	Randomized	5000	Recruiting
NCT04916002	Vidutolimod	TLR9	Advanced cancer or metastatic cancer	Cemiplimab	II	Open label	Non-randomized	200	Recruiting
NCT04401995	Vidutolimod	TLR9	Melanoma	Nivolumab	II	Open label	Randomized	36	Recruiting
NCT05445609	Vidutolimod	TLR9	Metastatic castration resistant prostate cancer	Nivolumab	II	Open label	N/A	10	Recruiting
NCT04695977	Vidutolimod	TLR9	Advanced melanoma	Nivolumab	II & III	Open label	Randomized	20	Active, not recruiting
NCT04698187	Vidutolimod	TLR9	Advanced melanoma	Nivolumab	II	Open label	N/A	44	Active, not recruiting
NCT04633278	Vidutolimod	TLR9	HNSCC	Pembrolizumab	II	Open label	N/A	44	Active, not recruiting
NCT03983668	Vidutolimod	TLR9	Relapsed and refractory lymphoma	Pembrolizumab	I & II	Open label	N/A	39	Recruiting
NCT04952272	CpG-ODN	TLR9	Advanced solid tumors	None	I	Open label	N/A	50	Recruiting
NCT04995536	CpG-STAT3 siRNA CAS3/SS3	TLR9	Relapsed/refractory B-cell non-Hodgkin lymphoma	Radiation Therapy	I	Open label	N/A	18	Recruiting
NCT04752722	EG-70	RIG-I	Superficial bladder cancer	None	I & II	Open label	Non-randomized	222	Recruiting

*PD-L1 inhibitor* Atezolizumab; Durvalumab; Avelumab, *PD-1 inhibitor* Nivolumab; Cemiplimab; Ezabenlimab; Dostarlimab; Pembrolizumab, *CTLA-4 inhibitor* Ipilimumab; Tremelimumab; Zalifrelimab, *LAG-3 inhibitor* Relatlimab, *VEGFR2 inhibitor* Ramucirumab, *mAb* monoclonal antibodies, *PULSAR* personalized ultra-fractionated stereotactic adaptive radiotherapy, *ICI* immune checkpoint inhibitor, *IO* immuno-oncology, *N/A* not avaliable, *Unknown* study has passed its completion date and status has not been verified in more than two years, *Available* expanded access is currently available for this investigational treatment, and patients who are not participants in the clinical study may be able to gain access to the drug, biologic, or medical device being studied

#### Agonists of TLRs

Long before the discovery of TLRs in the 1970s, Bacillus Calmette-Guérin (BCG), acting as an agonist for TLR2 and TLR4, was found beneficial in treating bladder cancer.^539,540^ This discovery was subsequently confirmed in a series of clinical trials.^541–543^ Presently, the FDA has approved BCG for the treatment of superficial bladder cancer. In addition to BCG, two other TLR agonists, monophosphoryl A, and imiquimod, have been approved for clinical use. Monophosphoryl A, an agonist for TLR4, is approved as an adjuvant for vaccines but has seen limited clinical trials concerning tumors.^544^ Imiquimod, targeting TLR7, is approved for treating genital warts and basal cell carcinoma, with its scope of application steadily expanding.^545,546^ A multicenter, randomized, phase 3 non-inferiority trial compared topical imiquimod to surgical treatment for vulvar intraepithelial neoplasia. The results indicated that imiquimod’s efficacy was comparable to surgery (NCT01861535).^547^ Moreover, the invasive disease was found in five patients who underwent initial or secondary surgeries, but none in those treated with imiquimod, suggesting possible advantages of imiquimod over surgery.^547^ Furthermore, imiquimod monotherapy has been found to combat skin metastases of breast cancer (NCT00899574).^548^ Similarly, another clinical trial demonstrated that combining a TLR7 agonist with albumin-bound paclitaxel could effectively induce remission in treatment-refractory metastatic breast cancer, albeit the response was transient (NCT00821964).^549^

Many other TLR agonists are actively undergoing clinical trials, some yielding promising results. The TLR3/RIG-I/MDA5 agonist, poly (I: C), has improved long-term survival rates in superficial bladder cancer patients.^550^ Intratumoral G100, a TLR4 agonist, demonstrated potential benefits in tumor treatment. Intratumoral injections of G100 resulted in an overall response rate of 33.3% and abscopal tumor regression in 72.2% of follicular lymphoma patients (NCT02501473).^551^ Additionally, G100 has been shown to induce antitumor immune responses and tumor regression in Merkel cell carcinoma patients.^552^ Other TLR4 agonists, such as HSPPC-96, a heat shock protein derived from patient GBM cells, may improve the prognosis of newly diagnosed GBM patients. A phase II single-arm clinical study showed that the standard GBM therapy combined with subcutaneous administration of HSPPC-96 resulted in a PFS of 18 months and OS of 23.8 months, improving prognosis (NCT00905060). Clinical trials targeting metastatic soft tissue sarcomas have shown promising results with the intratumoral injection of the TLR4 agonist glycopyranosyl lipid A in a stable-emulsion formulation, combined with concurrent radiotherapy, achieving effective local control of the tumor. All 12 patients involved in the study achieved local control after eight injections, including one case of complete remission. A notable observation in patients who experienced durable local responses was the enhanced clonal expansion of T cells. These T cells exhibited markedly increased expression of Tbet, indicating a Th1 phenotype (NCT02180698).^553^

Resiquimod, a TLR7/8 agonist, has shown potential benefits in treating early-stage cutaneous T-cell lymphoma. In a cohort of 12 recruited patients, 75% showed significant lesion improvement post-treatment, and 30% exhibited complete lesion clearance. Additionally, half of the patients displayed increased activation of circulating dendritic cells (NCT01676831).^554^ Moreover, clinical trials have revealed that the TLR8 agonist, Motolimod, can still effectively induce immune activation in late-stage cancer patients. The plasma levels of various biomarkers, including IL6, G-CSF, MCP-1, and MIP1-β, increase concomitantly with the escalating doses of Motolimod.^555^ Furthermore, combining cetuximab with Motolimod reduced MDSC infiltration in HNSCC, and enhanced infiltration of M1 TAMs and CD8^+^ T cells. These infiltrating T cells exhibited increased activation, including TCR clonal expansion, upregulation of the co-stimulatory receptor CD27, and downregulation of the inhibitory receptor TIGIT. Reduced induction of Tregs and a decrease in Treg suppressive markers like CTLA-4, CD73, and membrane-bound TGFβ were also observed (NCT02124850).^556^ This suggests that Motolimod might help overcome immune suppression in cancer patients. Using Motolimod on top of the standard therapy for recurrent and/or metastatic HNSCC did not improve progression-free survival (PFS) or overall survival (OS) in the intention-to-treat population, but significant benefits were observed in HPV-positive patients and those showing a response at the injection site, suggesting potential benefits of TLR8 stimulation in select patient subsets with specific biomarkers (NCT01836029).^557^

In a study investigating the intratumoral injection of the TLR9 agonist CpG-ODN for the treatment of recurrent GBM, it was observed that the number of long-term survivors increased among patients receiving CpG-ODN treatment. Specifically, a 1-year survival rate was 24% and a 2-year survival rate was 15%, compared to the typically observed rate of <15% in patients undergoing standard treatment. Consequently, although there was no overall improvement in median survival and PFS, the findings suggest that some patients may benefit from TLR9 agonist treatment.^558^ Furthermore, in a small-scale clinical trial, CpG-ODN was shown to successfully induce a Th1 adaptive immune response and cytotoxic activity in lung cancer patients. Interestingly, there was an increase in effector memory CD8^+^ T cells in patients receiving CpG-ODN.^559^

Another TLR9 agonist, MGN1703, exhibited immune activation and demonstrated anti-tumor efficacy in heavily pretreated patients with metastatic solid tumors.^560^ Further randomized controlled trial (RCT) results indicated that in small-cell lung cancer, MGN1703 was beneficial in two specific patient populations: those with a low frequency of activated CD86^+^ B cells and those who reported having chronic obstructive pulmonary disease.^561^ In a Phase II RCT for colorectal cancer, MGN1703 induced durable and prolonged PFS and disease control in a subgroup with relatively high levels of activated NKT cells.^562^ Additionally, a newly developed spherical nucleic acid compound targeting TLR9, known as cavrotolimod, exhibited the capability to effectively induce immune activation in a healthy population in a Phase I study, highlighting its potential anti-tumor effects (NCT03086278).^563^

Combined treatment with agonists of TLRs has also demonstrated good safety and potential anti-tumor activity (NCT00633529) (NCT00719199).^564,565^ It warrants further clinical investigation to ascertain if it can offer patients an improved prognosis than the standard therapy.

#### Agonists of RLRs

Several clinical trials have demonstrated the clinical benefits of poly-ICLC, which acts as a co-agonist for TLR3, RIG-I, and MDA5. For instance, In a phase II single-arm clinical trial, the median survival of newly diagnosed GBM patients treated with radiotherapy combined with poly-ICLC was 65 weeks, compared to 40 weeks for those receiving chemotherapy alone.^566^ Another multi-institutional phase II clinical trial showed that by combining temozolomide (TMZ) and poly-ICLC treatment, patients aged 18-70 had a median overall survival of 18.3 months (95% CI: 15.9–19.8 months), a benefit compared to 14.6 months (95% CI: 13.2–16.8) reported by the EORTC 26981/22981 trial.^567^

Clinical trials for MK-4621, a selective RIG-I agonist, have indicated that patients treated with either MK-4621 monotherapy or in combination with pembrolizumab exhibited tolerable safety profiles and moderate anti-tumor activities. Analyses of serum and tumor biomarkers provided evidence that MK-4621 treatment induced an increase in the gene expression of IFN signaling pathway members, associated chemokines, and cytokines. Regrettably, at the tested dosages, MK-4621 did not confer meaningful clinical benefits (NCT03065023) (NCT03739138).^568^

#### Agonists of NLRs

Clinical studies utilizing NLRs agonists for tumor interventions remain limited. Nevertheless, past results have offered some encouraging findings. Mifamurtide, a NOD2 agonist employed as an immunoadjuvant, has secured approval in the European Union for the treatment of osteosarcoma.^569^ By adding mifamurtide to the conventional chemotherapy regimen of cisplatin, doxorubicin, and methotrexate, there has been an enhancement in the event-free survival (EFS) for osteosarcoma patients, with the 6-year OS rate elevating from 70 to 78% (NCT00631631).^570^

### The safety of targeted interventions

Reviewing existing clinical trials, it has been observed that innate immune pathway agonists generally have good tolerability. The most common adverse reactions include local reactions at the injection site, such as pain, redness, itching, or induration, as well as systemic flu-like symptoms, which mainly manifest as fever, chills, headache, fatigue, and sometimes a combination of these.

Additionally, different types of drugs have their specific adverse reactions. For instance, the TLR8 agonist Motolimod can cause anemia and acne-like rashes, with severe treatment-related adverse events including vomiting and dehydration.^557^ In trials using poly-ICLC with TMZ for treating glioblastoma, common treatment-related grade 3–4 toxicities included neutropenia, leukopenia, thrombocytopenia, and rash.^567^ The dose-limiting toxicity of the RIG-I agonist MK-4621 was manifested as pleural effusion, and its combination with pembrolizumab could induce cytokine release syndrome (20%), anemia, hypertension, lymphopenia, dyspnea, and pleural effusion.^568^ The STING agonist MIW815 (ADU-S100) combined with Spartalizumab, in addition to fever and injection site pain similar to other innate immune agonists, characteristically causes diarrhea.^252^

Different agonists of the same receptor can also cause different adverse effects. The adverse reactions of the TLR9 agonist CpG-OND mainly manifest as lymphocytopenia, mild fever, seizures, and transient neurological deterioration.^558^ The TLR9 agonist, MGN1703, in addition to common adverse reactions, can also cause cough, erythema, dyspnea, neutropenia, nausea, asthenia, back pain, anemia, decreased appetite, and prolonged activated partial thromboplastin time. Severe adverse reactions include ileus, hypertension, worsening of hypertension, neutropenia, aspartate aminotransferase increase, sepsis, atypical pneumonia, and sensory polyneuropathy. Notably, although the polyneuropathy was thought to be possibly related to MGN1703, it was more likely related to previous oxaliplatin chemotherapy. Leukoencephalopathy has also been reported in a patient treated with platinum-based chemotherapy.^560–562^ The TLR9 agonist IMO-2055 has also been known to cause diarrhea, nausea, hypomagnesemia, dehydration, and stomatitis.^561^ The combination of the TLR9 agonist SD-101 with Pembrolizumab can induce vomiting, constipation, and increased γ-glutamyltransferase. Treatment-related serious adverse events include atrial fibrillation, systemic inflammatory response syndrome, infusion reactions, and dehydration. Newly developed immune-related adverse reactions include pneumonia, polymyalgia rheumatica, hypothyroidism, and hypophysitis.^571,572^

Despite most clinical trials reporting good safety profiles for innate immune pathway agonists, most of these trials have small sample sizes. Therefore, caution should still be exercised in assessing their safety, and larger-scale trials are needed to fully evaluate their safety profiles.

### Function as adjuvant of vaccines

In recent years, therapeutic cancer vaccines have made significant advances. However, tumor-induced immune suppression and immune resistance present significant challenges to its therapeutic efficacy.^573^ Vaccine adjuvants play a crucial role in enhancing the immune activation capabilities of vaccines. They activate the innate immune system, profoundly influencing APC maturation and CTL activation and proliferation.^574^ Both preclinical and clinical studies have highlighted the potential of innate immune pathway agonists as adjuvants to amplify the anti-cancer effects of vaccines. In a HER2^+^ breast cancer mouse model, adding a STING agonist to a novel peptide vaccine targeting heat shock protein 90 fostered T-cell rearrangement of TCRβ, prolonging mouse survival.^575^ Furthermore, a vaccine consisting of STING agonist 2’3’-cGAMP, TLR9 ligand CpG, and tumor antigen peptides loaded into nanoporous microparticles effectively inhibited tumor growth. Both TLR9 and STING agonists enhanced APC IFN-β and TNF-α expression, with TLR9 being indispensable for the vaccine’s anti-tumor response.^576^ Poly IC and its derivative Poly ICLC, acting as agonists for TLR3, RIG-I, and MDA5, are widely utilized in cancer vaccines, aiding tumor eradication in both animal tumor models and patients.^577^ One clinical study revealed that treating newly diagnosed GBM patients with a combination of Temozolomide and Poly ICLC resulted in a median survival of 17.2 months, surpassing existing standard treatments (NCT00262730). Additionally, a trial using low-dose reirradiation combined with poly (I:C) and GM-CSF in recurrent WHO grade IV glioma patients reported those responding to poly (I:C) exhibited significantly extended PFS and OS than non-responders, accompanied by an uptick in CD8^+^ T and NK cells (NCT03392545).^578^

Combining various innate immune pathway agonists can further bolster the vaccine’s potency. A clinical trial focusing on using a long peptide vaccine to treat resected high-risk melanoma showed that co-administering poly ICLC and incomplete Freund’s adjuvant, compared to solely employing the TLR7/8 agonist resiquimod, amplified the CD8^+^ T-cell immune response rate (NCT02126579).^579^ Consistently, in another clinical study, the NY-ESO-1 Protein Vaccination elicited a CD8^+^ T cell response only when combined with resiquimod (3 out of 12, 25%), though the sample size was limited.^580^

Beyond peptide vaccines, innate immune agonists also enhance the therapeutic efficacy of DC vaccines. Stimulation of DCs with a cocktail containing Poly I:C or resiquimod, TNF-α, IL-1β, and IFN-γ can make DCs resistant to TGF-β2, preserving MHC-II expression and IL-12 release, hence boosting DCs’ ability to activation T cell and tumor-inhibitory effects.^581^ A clinical trial using a dendritic cell vaccine with poly (I:C) and imiquimod as adjuvants reported a median survival of 19 months for GBM patients, suggesting a favorable prognosis relative to patients receiving standard treatment at the same facility (NCT02709616)(NCT02808364).^582^ Another DC vaccine clinical study targeting advanced cancer patients demonstrated that intratumoral application of poly ICLC heightened IFN-β and IFN-α mRNA levels in circulating peripheral blood mononuclear cells (PBMCs).^583^

In recent years, mRNA vaccines have garnered increasing attention, and components of innate immune agonists can be used as adjuvants to further enhance the efficacy of these vaccines. For instance, the incorporation of the Toll-like receptor 2/6 agonist Pam2Cys has been found to enhance the anti-cancer effects of mRNA vaccines and establish a Pam2Cys-dependent long-lasting immune memory.^584^

It is noteworthy that the method of vaccine administration can affect its therapeutic efficacy. Recent research has indicated that vaccines containing tumor antigen peptides and a TLR7/8 agonist exhibit superior anti-tumor effects when administered intravenously compared to subcutaneous injection. Intravenous injection enhances the systemic secretion of type I IFN, a feature not present in subcutaneous injection. This increase in type I IFN can remodel the TME, reducing the presence of immunosuppressive TAMs.^585^

In summary, innate immune agonists have shown commendable efficacy in enhancing the therapeutic outcomes of tumor vaccines. These findings warrant further research to construct the optimal combination of agonists.

### Intervention of microbiota, the nature ligands

Many tumors contain microbial components, whose structural constituents and secretions are natural agonists of the innate immune pathway.^586^ Modulating the tumor’s microbial community holds the potential for regulating the tumor’s immune response and prognosis. In fact, there is mounting evidence that interfering with the tumor’s microbiome can improve its prognosis. In lung adenocarcinoma, germ-free or antibiotic-treated mice were significantly protected from lung cancer development induced by Kras mutation and p53 loss. The pro-tumorigenic effect of commensal bacteria likely depends on their activation of the TLRs pathway in bone marrow cells, as chimeric mouse analysis showed that the absence of Myd88 in bone marrow donors inhibited tumor growth. Downstream products of bone marrow cell TLRs, such as IL-1β and IL-23, can induce activation and proliferation of γδ T cells, mediating chronic inflammation and thus promoting lung cancer development.^587^ Another study based on an intraperitoneal colorectal cancer and melanoma transplant tumor model in mice showed that oral administration of *Lactobacillus rhamnosus* GG (LGG) could promote IFN-β production by inducing the DCs’ cGAS/STING/TBK1/IRF7 axis. This enhanced the cross-priming of anti-tumor CD8^+^ T cells, promoting anti-tumor immunity. Combined treatment with LGG and PD-1 inhibitors shifted the gut microbial community towards enrichment in *Lactobacillus murinus* and *Bacteroides uniformis*, known to increase DC activation and CD8+ tumor recruitment and was consistent with the better prognosis of the combined therapy. Interestingly, this microbial community change was not observed when either of the drugs was used alone, suggesting a deeper interaction between the two.^588^

Moreover, the tumor microbiome also regulates the tumor’s response to therapies like chemotherapy and radiotherapy. For example, *Fusobacterium (F.) nucleatum* in the colorectal cancer tissue of patients with post-chemotherapy relapse can target the TLR4/MyD88 signaling pathway, which downregulates microRNAs that inhibit autophagy, thereby activating tumor cell autophagy and inhibiting apoptosis, leading to chemotherapy resistance. Thus, targeting the reduction of *F. nucleatum* is a potential therapy to improve chemotherapy resistance in patients.^589^ The gut microbiome can also affect the radiotherapy efficacy in HCC patients. The gut microbiome regulates the liver cancer RT sensitivity through the cGAS-STING signaling in DCs. It was found that bacterial-origin STING agonists such as C-di-Amp were significantly higher in the liver cancer tissues of RT-sensitive patients than in those resistant to RT. Therefore, the gut microbiome may influence the sensitivity of tumors to chemotherapy by regulating the activation level of the cGAS-STING pathway.^590^

Intervening in tumor therapy by modulating the tumor’s microbiome offers several advantages. Firstly, this approach is relatively simple and accessible. For gastrointestinal tumors, beneficial bacteria can be increased through oral administration of probiotics containing the relevant microbes, or harmful bacteria can be reduced using specific phages and antibiotics with targeted spectra.^591^ Additionally, the microbiome can activate multiple innate immune pathways simultaneously, creating synergistic effects across multiple targets. The modifiability of microbes also allows for multi-target synergistic effects. For instance, an attenuated *Salmonella typhimurium* strain engineered to secrete *Vibrio vulnificus* flagellin B (FlaB) in tumor tissues can activate both TLR4 and TLR5. The activation of TLR4 and TLR5 has a synergistic effect, where TLR4 signaling induces the infiltration of numerous immune cells like monocytes/macrophages and neutrophils, while the TLR5 pathway activates M1 TAMs and inhibits M2 TAMs.^592^

However, there are several considerations in manipulating the tumor microbiome. It’s essential to determine how specific bacterial strains influence tumor development. The same bacterial species may have different effects on various tumors or under different microbial community backgrounds. The safety of altering the microbial community must also be considered, including potential adverse reactions to introducing specific microbes. An intriguing aspect is whether methods to improve tumor prognosis by modulating the microbiome should focus on altering the tumor’s internal microbial community. Intervention in the gut microbiome has been found to affect the sensitivity of liver tumors to chemotherapy.^590^ Studies on the gut-liver axis and gut-brain axis suggest that intervening in the more easily modifiable gut microbiome may impact tumor development in distant organs.^590,593^ Research in this area could also enhance the safety of microbiome intervention therapies, as introducing bacteria into sterile organs like the brain or bone marrow poses significant ethical and safety challenges.

### Combination therapies focusing on upstream potentiation

#### Increasing production of DAMPs

DAMPs serve as natural activators of the innate immune pathway. Therapies that enhance the production of DAMPs not only intensify the activation of the innate immune pathway but also bolster its persistence. Moreover, such strategies allow for a reduction in the dosage of activators, consequently mitigating adverse reactions. Radiochemotherapy stands as the most clinically prevalent method for stimulating DAMP production.^594–596^ Additionally, radiochemotherapy facilitates the release of tumor antigens, thus promoting the antigen presenting of APCs.^594^ In murine models of pulmonary metastasis stemming from melanoma and breast cancer, the combined application of STING and chemotherapy markedly amplified the anti-cancer effects of STING. This combination initiated a systemic anti-cancer immune response, managed metastasis in both lungs and established long-term immune memory.^597^ Consistently, results from combining TLR7/8 agonists with radiotherapy showcased an enhancement in the effects of the TLR7/8 agonists. In this immunotherapeutic approach, DCs, which play a pivotal role in antigen presentation and cytotoxic cell activation, are at the epicenter.^598^ Clinical trials have also indicated that intratumoral injections of the TLR9 agonist, CpG ODN, in combination with radiotherapy for the treatment of mycosis fungoides, resulted in a decrease in immune-suppressive cell infiltration in one-third of patients warrant further study (NCT00226993).^599^

Beyond radiochemotherapy, inhibitors of DNA repair enzymes can also be harnessed to boost DAMP production. In a BRCA1-deficient breast cancer model, the concurrent use of the DNA repair enzyme PARP inhibitor and the STING agonist significantly amplified the anti-tumor effects of STING. Furthermore, the administration of STING reinstated the BRCA1-deficient breast cancer’s sensitivity to the PARP inhibitor.^600^ In certain tumors characterized by DNA repair deficiencies, the therapeutic efficacy of this combination can be further magnified. For instance, in a model of pediatric high-grade glioma with H3.3-G34 mutations, impaired DNA repair led to the buildup of extrachromosomal DNA. By capitalizing on this weakness, the life span of mice was considerably extended by amalgamating the STING agonist, radiation therapy, and DNA damage response inhibitors. Furthermore, there is an induced immune memory in those who survived for extended periods.^601^

Beyond the methods previously mentioned, there are alternative techniques for inducing immunogenic cell death. One such method is irreversible electroporation, a treatment that involves introducing electrodes into tumors to deliver electric pulses, prompting tumor cell apoptosis.^602,603^ Another approach is the use of apoptosis-inducing agents, such as combining TRAIL receptor agonists—which induce apoptosis—with sensitizers of the TRAIL-induced apoptotic pathway, like bortezomib, a proteasome inhibitor.^604^ These combined strategies have demonstrated superior efficacy compared to monotherapies that solely utilize innate immune activators.^602–604^

#### Promoting phagocytosis

The activation of T cells by APCs hinges on the presentation of the antigen and the transmission of co-stimulatory signals.^605^ Therefore, the uptake and processing of antigens—a prerequisite for antigen presentation—are pivotal for immune activation. By co-administering drugs that promote the phagocytosis of tumor cells or tumor antigens by APCs, the functional integrity of APCs, which are central to the immune response, can be enhanced. Moreover, the APC’s processing of tumors can release DAMPs, stimulating the activation of endogenous innate immune pathways.^606^ Furthermore, the enhancement of APC phagocytic activity can also increase the uptake of exogenously provided agonists, amplifying their effective dose.^607^ However, tumor cells have developed mechanisms to evade phagocytosis, notably by upregulating the anti-phagocytic molecule CD47.^608^ Based on this, researchers have crafted a blood-brain barrier-permeable nanocapsule to deliver both anti-CD47 antibodies and STING agonists directly to gliomas. This approach notably increased the polarization towards the M1 phenotype within gliomas and hindered GBM growth. Impressively, its efficacy surpassed treatments utilizing only STING agonists or CD47 antibodies.^609^ Another study, which combined TLR7 agonists, CD47 antibodies, and radiation, also demonstrated its potential in promoting M1-type polarization of TAMs and exhibited remarkable antitumor efficacy.^610^ Beyond CD47 antibodies, other phagocytosis-promoting agents, such as phagocytosis-activating ligands, mannans, have also shown promising antitumor effects when used in conjunction with innate immune pathway agonists.^611,612^

#### Reopening Signaling Pathway

The prerequisite for the use of innate immune pathway activators is the normal expression of all components of the innate immune pathway in cells. However, the TME can induce the suppression of the innate immune pathway through various means, such as by epigenetically inhibiting the expression of key proteins of the innate immune pathway.^256,257^ For instance, the expression of STING is epigenetically repressed due to methylation at cg16983159 in glioma cells. By applying, decitabine, a DNA methyltransferase inhibitor, STING expression can be restored, thus reinvigorating STING’s responsiveness to its agonist, cGAMP.^256,257^ In a separate study, the combination of zebularine (a demethylating agent) and the STING agonist cGAMP effectively induced ISG expression and augmented the infiltration of CD8 T cells and NK cells into tumors, consequently inhibiting tumor growth and prolonging mouse survival. This combination demonstrated a significantly superior outcome compared to using cGAMP alone.^613^ Furthermore, aberrant protein expression or anomalous levels of protein expression can lead to irregularities in the innate immune pathway. For example, the abnormal binding of PP2A to MST1/2 in TAMs can suppress the STING pathway. Inhibiting or knocking out PP2A in TAMs enhances their responsiveness to STING agonists, leading to increased IFN production.^262^

As discussed in the section “Innate Immune Pathway in Cancer”, numerous components of the TME can induce abnormalities in the innate immune pathway. By jointly targeting these pathways, there’s a potential to enhance antitumor immune activity.

### Combination therapies targeting downstream deinhibition

#### Immune checkpoint blockers

The synergistic effect of combining innate immune pathway agonists with immune checkpoint inhibitors (ICBs) in cancer therapy is mechanistically traceable. The anti-tumor response caused by the “release of the brakes” on CTLs and NK cells through ICBs can be amplified by more active CTLs and NK cells. Innate immune agonists, in fact, have an immunostimulatory effect. For instance, STING is essential in inducing an IFN-dependent T cell anti-tumor response,^238^ while TLRs are necessary for the maturation of DCs.^371,372^ Additionally, the effectiveness of ICBs depends on the sufficient expression of corresponding receptors on target cells, and innate immune pathway agonists can upregulate the expression of these molecules.^218,220,499,524^

These effects have been confirmed in many preclinical studies in rodent models. For example, a vaccine with a STING agonist-induced regression of palpable, poorly immunogenic tumors that did not respond to PD-1 blockade alone. Significant upregulation of PD-L1 was observed in tumors treated with the STING agonist, sensitizing them to PD-L1 inhibitors. The vaccine’s anti-tumor activity was STING-dependent and associated with increased activation of DCs and tumor antigen-specific CD8^+^ T cells. Interestingly, mice treated with the combination therapy gained protection against tumor rechallenge, suggesting that the combination therapy led to long-term tumor-specific memory, which was not observed in any single therapy.^220^ It is noteworthy that the formation of immune memory by STING agonists does not necessarily depend on ICBs. This was observed in a mouse model of glioma, where the administration of STING agonists alone was sufficient to establish long-lasting immune memory. However, it’s important to note that in this study, STING agonists were administered intratumorally, which might provide a more potent stimulation. Additionally, the primary effector cells activated by STING in this context were NK cells, as evidenced by the fact that the depletion of NK cells abrogated the tumor-eliminating effects of the STING agonists. The dependence of STING-induced immune memory on ICBs may vary depending on several factors, such as the tumor background, the intensity of the agonist stimulation, and the types of effector cells predominantly involved.

Consistent with STING agonists, RIG-I activation also upregulated tumor cell PD-L1 expression, making the combination with PD-L1 inhibitors more effective than either treatment alone. Intriguingly, RIG-I activation alone could induce long-term immune memory, and the persistence of adoptive T cell anti-tumor response proved that T cells had acquired immune memory.^499^ The combination of innate immune pathway agonists with ICBs also inhibited the growth of distant tumors, suggesting their potential role in inhibiting the development of metastatic tumors. A study combining TLR7/9 agonists with PD-1 inhibitors in HSCNN showed that their combination not only promoted local tumor regression but also enhanced the regression of distant and recurrent tumors.^614^ STING activation was necessary for CTLA-4-mediated regression of distant melanoma tumors, as tumor cell STING knockout prevented the enrichment of intratumoral CD8^+^ T cells in distant tumors, leading to faster tumor growth and poorer prognosis.^571^ Investigating how the combination of innate immune pathway agonists and ICBs generates or enhances immune memory has tremendous potential value in preventing tumor recurrence and metastasis. However, the specific mechanisms are still unknown. Analyzing changes in T cell phenotype may provide useful clues, as many studies have reported that combination therapy further increases the number or proportion of activated cells.^220,572,614^ Moreover, the main effector cells in combination therapy are disputed. In many studies, combination therapy increased the number of IFN-γ + CD8 + T cells.^220,572,614^ In HSCNN, the combined effect of TLR7/9 agonists and PD-1 was abrogated by CD8 antibody depletion of CTLs, indicating that its anti-tumor effect mainly relies on CTLs.^614^ However, in a melanoma lung metastasis model, the combined effect of a STING agonist and PD-1 was not abrogated by CD8^+^ cell depletion but depended on NK1.1^+^ cells, suggesting that its combined anti-tumor effect mainly relies on NK cells.^615^ Therefore, the effector cells of combination therapy may also be pathway and tumor type-dependent. In summary, the combination therapy is not a simple additive effect but involves profound interactions and synergistic effects.

Several clinical investigations have also highlighted the potential benefits of such combined treatments. For instance, a Phase Ib trial involving inhaled DV281, a TLR9 agonist, in combination with Nivolumab in patients with advanced or metastatic non-small cell lung cancer indicated sustained control in 50% of the participants.^616^ Another study demonstrated that the combined use of the TLR9 agonist Vidutolimod with PD-1 blockade resulted in durable responses in 25% of the patients. Remarkably, tumor regression was noted in both injected and non-injected sites, encompassing visceral lesions.^617^ For head and neck squamous cell carcinoma (HNSCC), the combined use of the TLR9 agonist SD-101 and PD-1 blockade was found to be safe and induced a certain objective response rate (24%), even in patients with low baseline IFNγ-related gene expression in tumors (NCT02521870).^618,619^ Notably, a higher objective response rate of 44% was observed in HPV^+^ patients. Moreover, this combination treatment led to a sustained increase in the expression of genes representing various immune cell types, including CTL and NK cells.^618^ Clinical trials that combined the STING agonist MIW815 (ADU-S100) with the PD-1 inhibitor Spartalizumab indicated that, out of 106 patients, 11 (10.4%, 90% confidence interval (CI) 5.9-16.6) achieved an objective response, either confirmed complete remission (CR) or partial remission (PR). The disease control rate, which is the proportion of patients showing either a response or stable disease, stood at 29.2% (90% CI, 22.0–37.4). The study, however, was prematurely terminated due to its limited efficacy. Nonetheless, an upsurge in pro-inflammatory factors, including IFNβ, and an enhanced infiltration of CD8^+^ T cells was documented (NCT03172936).^252^ Manganese ions, acting as agonists of the cGAS-STING pathway, when combined with PD-1 antibodies, have shown preliminary favorable clinical efficacy with a 45.5% (95% CI, 26.9–65.3) best objective response and a 90.9% (95% CI, 72.2–97.5) best disease control rate. Notably, all five patients who had previously failed combination treatment with PD-1 antibodies and chemotherapy or radiotherapy showed disease control, including three cases of partial response (PR) and two of stable disease (SD). This suggests that Mn2+ may restore the effectiveness of ICB therapies in these immunologically unresponsive patients (NCT03991559).^620^

Overall, the combination therapy of innate immune agonists and ICBs has demonstrated significant immune activation in humans, evidenced by increased levels of inflammatory factors. However, why this response does not translate into better patient prognosis in some cases is one of the key points for further analysis. Understanding the mechanisms that underlie this discrepancy could be crucial for optimizing the use of such combination therapies in cancer treatment.

#### Pro-tumoral bypass inhibitors

The activation of intrinsic immune pathways can also induce certain pro-tumoral pathways, potentially diminishing or even reversing the effects of intrinsic immune pathway agonists.^12,221,621^ Consequently, co-administration with inhibitors targeting these bypass mechanisms can better unleash the potential of intrinsic immune pathway activators. For instance, STING agonists can enhance IDO activity, leading to immune suppression.^221^ By combining with an IDO inhibitor, the anti-tumor effect of the STING agonist is substantially amplified in murine models of colorectal cancer.^622^ Furthermore, the type I IFN produced by the STING pathway induces the expression of CCL2, CCL7, and CCL12, promoting the recruitment of CCR2-expressing MDSCs to tumors and thus fostering immune suppression. The co-administration with a CCR2 inhibitor markedly enhances the tumor suppression induced by cGAMP and radiation therapy, leading to prolonged survival in tumor-bearing mice models.^621^

The concept of bypass activation of STING, particularly in chromosomal instability (CIN) tumors, presents a notable example. Studies have shown that the downstream product of the cGAS-STING pathway, IL-6, can activate the STAT3 signaling pathway in tumors, thereby promoting tumor growth. The use of IL-6R inhibitors has significantly inhibited tumor growth.^219^ Therefore, the combination of STING agonists with IL-6 inhibitors may be a potential future therapy.

Additionally, chronic activation of the cGAS-STING pathway induced by CIN leads to a reorganization of downstream signaling in cancer cells. This reorganization is characterized by a selective blunting of the type I IFN pathway downstream of STING and a corresponding increase in cancer cell-derived endoplasmic reticulum (ER) stress responses. This shift results in a tumor-promoting effect of STING activation. Researchers have proposed using STING inhibitors to suppress the development of CIN tumors, a strategy that has been validated in mice.^12^ However, an intriguing idea is whether the combined use of STING agonists and ER stress response inhibitors could redirect the downstream pathway towards the production of type I IFN, thereby leveraging the anti-tumor effect of the STING pathway. This approach could potentially provide a novel way to manipulate the complex signaling dynamics in cancer cells for therapeutic benefit.

In addition to the combination strategies mentioned above, innate immune pathway agonists can also enhance the efficacy of many other therapies, such as CAR T therapy.^383,623,624^ Given the multitude of downstream effects innate immune pathway agonists possess, and their ability to elevate the baseline level of immune activation, there are numerous potential combination strategies worth investigating. There are already many clinical trials of innate immune pathway agonists combined with other therapies (Table 3). The combined use of different innate immune pathway agonists also represents a promising avenue for further research.

## Challenges and solutions: precise harness of innate immune pathways

**Fig. 6:**
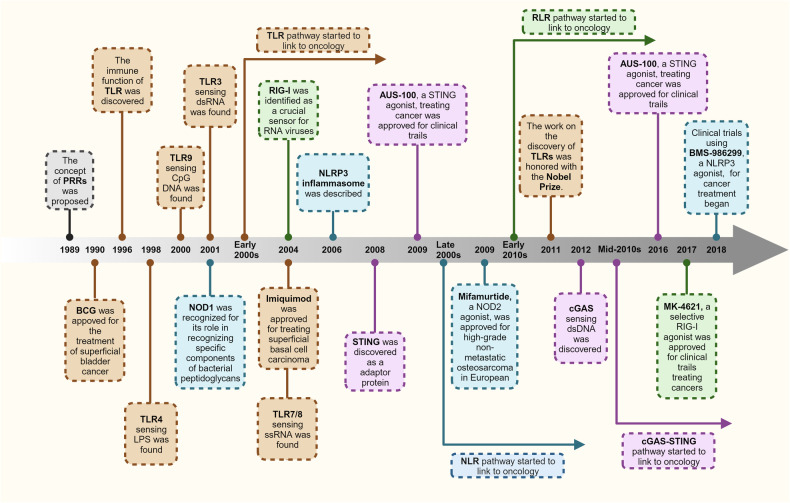
Innate immune sensors and their pathways in the nucleus. (1) hnRNPA2B1 recognition of dsDNA in the nucleus is followed by JMJD6-induced demethylation, promoting its translocation to the cytoplasm. There, hnRNPA2B1 is recruited to STING, triggering downstream activation. (2) SAFA identifies dsRNA and homodimerizes to bind to multiple SAFA binding sequences in the IFNB1 enhancer, promoting the transcription of type I IFNs. (3) Upon recognizing dsDNA, IFI16 relocates to the cytoplasm to activate the STING pathway. It also recruits ASC and caspase-1 to form inflammasomes. (4) ZBP1 detects Z-RNAs, activating RIPK3. Subsequent RIPK3 activation induces cell apoptosis and phosphorylates MLKL. Phosphorylated MLKL disrupts nuclear and cellular membranes, leading to necroptosis. Created with BioRender.com

Since the discovery of the innate immune pathways, our understanding of them has deepened progressively, leading to numerous preclinical and clinical trials in the field of cancer therapy (Fig. 6). However, significant breakthroughs in clinical applications using innate immune pathway agonists have yet to be realized. In this section, we summarize the challenges and potential solutions concerning the application of innate immune pathways in cancer treatment.

### Potency, dosage, tolerance, and precision delivery

**Fig. 7:**
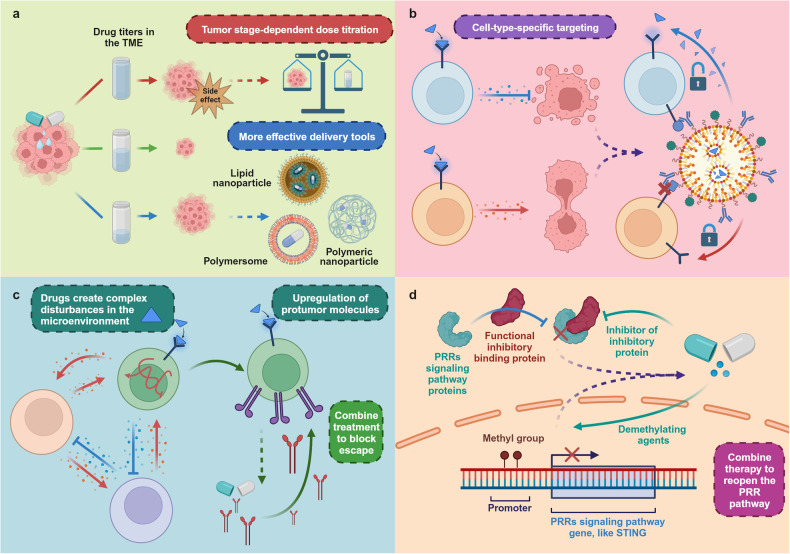
Obstacles and solutions for tumor treatment with innate immune pathway agonists. **a** Adjusting dosage and delivery methods to achieve optimal therapeutic effects. **b** Selectively targeting cell types that possess anti-tumor activities upon activation of innate immune pathways. **c** Overcoming immune escape caused by bypass activation of agonists through combination therapies. **d** Correcting aberrant innate immune pathways. Created with BioRender.com

Innate immune pathway agonists, when utilized as medications, display variable pharmacological effects on the same system, influenced by factors such as potency, dosing interval, and concentration. The potency of a drug is instrumental in determining its therapeutic outcome. For instance, DMXAA (5,6-Dimethylxanthenone-4-acetic acid), an agonist for the STING pathway, demonstrates a high affinity for murine STING but not for its human counterpart.^625^ This disparity could explain the largely suboptimal performance of DMXAA in early clinical trials for tumor treatments.^236^ Another illustrative example is the differential potency among various TLR9 agonists. While CpG-A selectively promotes the differentiation of pDC subtypes specialized in IFN-I secretion, other TLR9 agonists, like Resiquimod and CpG-B CpG-C, do not possess this characteristic.^626^ As a result, drugs developed from CpG-A, such as vidutolimod, have heightened IFN type I production capacity and can robustly stimulate both CD4^+^ and CD8^+^ T cells. This might clarify why vidutolimod is the sole TLR9 agonist that has demonstrated clinical activity as a monotherapy in advanced anti-PD-1 refractory melanoma patients.^617^ In contrast, the Phase III clinical trial involving the combination of CpG-C oligodeoxynucleotide tilsotolimod and ipilimumab failed to show improvements in advanced PD-1 blockade-refractory melanoma, potentially due to these distinctions (NCT03445533). Distinct LPS chemotypes within TLR4 also differentially influence NF-κB and IRF3 activities and their subsequent target gene expressions.^627^ These evidences underscore the importance of developing highly effective and specific agonists for human use (Fig. 7).

Immunological tolerance is inherent within the innate immune pathways. This tolerance, identified as reduced or non-reactivity upon agonist stimulation following repeated, prolonged, or chronic activation, can be observed in many innate immune pathway receptors. Furthermore, there’s evidence of cross-tolerance where the prior activation of one receptor induces tolerance in another.^313^ Strategies such as modifying dosing intervals can circumvent this immune tolerance. For example, preclinical studies have shown that infrequent, low-dose administration of TLR7/8 agonists can better induce tumor elimination compared to frequent administrations.^628,629^ Additionally, more effective immune activation can be achieved by sequentially stimulating receptors that do not exhibit cross-tolerance. For example, while RLRs utilize non-MyD88-dependent pathways and most TLRs employ MyD88-dependent pathways, there is minimal cross-tolerance between them. Consequently, sequential stimulation of RLR and TLR can lead to enhanced immune activation.^630^

Drug dosage plays a pivotal role in therapeutic outcomes. Different dosages can induce varied anti-tumor effects. For example, low doses of viral dsRNA, when activating the RIG-I receptor, lead to minimal IFN and proinflammatory cytokine secretion, subsequently encouraging NF-κB- and Akt-driven cell proliferation, migration, and invasion in HNSCC.^631^ As the downstream production of the innate immune pathway, IL-12 can enhance the antitumor activity of CD8^+^ T cells in tumor-bearing mice at low doses, while high doses of IL-12 inhibit the endogenously generated CD8^+^ T cells response and promote tumor growth.^632^ IFN-α, a crucial downstream molecule in the innate immune pathway, also exhibits dose-dependent effects. Studies have found that high concentrations of IFN-α activate the STAT1 pathway, inhibiting tumors, whereas low concentrations of IFN-α can simultaneously activate the pro-tumorigenic STAT3 pathway.^219^ There are some clinical indications to support this notion. Besides activating TLR3/RIG-I/MDA5, poly-ICLC also stimulates OAS (2’5’oligoadenylate synthetase) and PKR (protein kinase R). Higher doses of dsRNA can inhibit the OAS and PKR systems. This could explain why, in early cancer trials, higher doses of poly-ICLC were relatively ineffective.^566^

Naturally, drug distribution profoundly affects therapeutic efficacy. Preclinical research indicates that in situ administration of the TLR9 agonist CpG significantly enhances tumor suppression, whereas intravenous and subcutaneous administration (at distant sites) is markedly less effective, possibly due to differences in drug distribution.^633^ Localized injections are advantageous as they allow immune cells infiltrating near the tumor antigen to be effectively stimulated under the high concentration of CpG, thereby eliciting a robust immune response. However systemic injections lack such specificity and might even induce T cell immunosuppression.^634^ This evidence underscores the significance of anatomically specific drug distribution. This has led to efforts to devise more targeted delivery methods or develop specialized delivery systems. For instance, researchers have developed bioengineered ferritin nanoparticles that can bind to transferrin receptor 1, which is overexpressed in blood-brain barrier endothelial cells.^635^ These nanoparticles are transported into the brain through transcytosis and achieve glioma-specific targeting by fusing different glioma-targeting moieties on the nanoparticles. The delivery of a STING agonist through this system significantly inhibited tumor growth and extended the survival time of mice.^635^ Besides, various other delivery systems, including nanocarriers, microparticles, and hydrogels, are under development and have shown superior anti-tumor activity in preclinical studies compared to traditional methods.^636–638^

### Tumor traits and personalized agonist efficacy

The characteristics of a tumor play a pivotal role in the efficacy of agonists. Activation of the intrinsic immune pathway hinges on a malleable immune cell population. As the overall tumor burden escalates, the level of tumor-induced immunosuppression diminishes.^639^ In advanced tumor stages, a large proportion of immune cells enter a state of exhaustion, rendering immunotherapy potentially less effective under a high tumor load. Preliminary clinical studies have indicated divergent outcomes when administering agonists at different tumor developmental stages. For instance, in a murine model of transplanted melanoma, prophylactic application of TLR4/9 agonists (before tumor inoculation) can activate the IFNγ/STAT1 signaling pathway, promoting tumor cell autophagy and autophagy-related tumor cell death, subsequently reducing metastasis. In contrast, therapeutic application (post-tumor inoculation) is rendered ineffective in tumor suppression due to the pre-existing activation of STAT3 in tumor cells and the production of IL-10, which inhibits the IFNγ/stat1 pathway.^640^ Another study revealed that while prophylactic use of TLR5 agonist flagellin promotes tumor growth, its therapeutic application inhibits it. This dichotomy might be attributed to the TLR5 agonist inducing differential Th cell differentiation in varying contexts—prophylactic use tilts towards Th2 differentiation while therapeutic application elicits a Th1 response.^641^

Moreover, the type of tumor impacts the efficacy of the agonist, as illustrated in previous texts and Table 1. Different tumor types might react distinctively to the same agonist. Furthermore, various characteristics can influence treatment outcomes even within a single tumor type. Of particular note are tumors with high CIN characteristics. The balance between the classical and non-classical NF-κB pathways induced by STING is affected by the TME. In contrast to the RelA-mediated classical NF-κB pathway, the RelB-driven non-classical NF-κB pathway inhibits the release of type I IFNs.^642^ Chronic stimulation of the cGAS-STING by CIN tumors causes a dominance of the non-classical NF-κB pathway in tumor cells, which in turn facilitates tumor metastasis through STING activation.^13^ A recent article also reported that tumor cells promote metastasis by activating the endoplasmic reticulum stress response downstream of STING.^12^

Furthermore, the genetic attributes of patients also influence the effectiveness of agonists. Polymorphisms in receptors of the intrinsic immune pathway have been found to impact both the pathway’s activation and the patient’s prognosis.^643,644^ Basic demographic factors such as age and gender can also influence the activation of this pathway. For example, male populations exhibit lower RIG-I expression than females, which has been linked to higher incidences of liver cancer in males. Knocking out RIG-I offsets this disparity.^488^

Clinical evidence also indicates that a particular treatment method may prove more effective for specific patient subgroups (NCT01836029) (NCT02521870).^557,561,562,618,619^ Therefore, establishing efficient tumor typing and patient grouping strategies is imperative for enhancing the therapeutic effects of intrinsic immune pathway agonists.

### Cell-type-specific responses

The activation outcomes of innate immune pathway receptors are specific to certain cell types. In B cells, for instance, TLR activation propels mitosis. Nonetheless, TLRs do not play a role in the mitotic process of other cells.^645^ Different STING signaling intensities also influence a cell’s apoptotic tendencies. In T cells, the higher expression of STING compared to macrophages predisposes them to apoptosis post-STING activation.^192^ Moreover, the downstream products following TLR activation are also reliant on the cell type. For example, while TLR4 activation in human monocytes leads to the production of IL-1β, this response is absent in MDMs.^646,647^ Different cell types within the TME manifest distinct responses when activated by the innate immune pathway as discussed in the previous section and Table 1. For instance, while TLR2 in microglia enhances CD8^+^ T cell infiltration and activation, thereby inhibiting glioma via the TLR2-MHC-I axis,^648^ its activation in glioma cells bolsters tumor development by augmenting autophagy.^649^ Additionally, the expression and levels of TLRs differ across cell types. Human mDCs, for example, express all TLRs except for TLR9, whereas human pDCs predominantly express TLR9.^650^ As a result of these mechanisms, innate immune pathway agonists might exhibit dual roles within the TME. In particular niches, the dominance of immunosuppressive cells and tumor cells might outweigh their anti-tumor actions, leading to suboptimal local therapeutic outcomes and tumor immune evasion.

Given this understanding, crafting cell-type-specific drug delivery methodologies becomes imperative. Dual-target or multi-target mediated active targeting systems emerge as a viable option.^651,652^ Such systems function by integrating diverse functional ligands onto delivery carriers. One targets tissue-specific markers, while others zero in on specific cell types that express unique receptors.^652^ This approach ensures selective activation of the innate immune pathway in distinct cell types, thereby maximizing the anti-cancer potential of innate immune pathway agonists (Fig. 7).

### Combination therapy and reprogramming innate immune pathways

The cellular response elicited by the activation of innate immune pathways is intricate, due to the presence of interwoven intracellular networks that often display overlapping, converging, alternative, and redundant routes. Directly targeting a specific anti-tumor mechanism in treatment via the activation of a solitary or a distinct set of receptors is typically daunting. Instead, such stimulation inputs often induce adaptive changes in the signal network of the tumor, silencing or hijacking the stimulus signal. One salient instance is the heightened IFN signaling provoked by innate immune pathway activation. This can escalate the expression of multiple T-cell inhibitory receptors via IFN receptor signaling, hence suppressing the anti-tumor immune response.^653,654^ To counter this, combination therapy can be employed to impede these pro-tumor alternate pathways, thereby more effectively unleashing the anti-tumor potential of activated innate immune pathways. It’s noteworthy that combination therapy can be continually adjusted based on the evolving condition of the patient, given the constant occurrence of immune evasion. For example, protracted IFN signaling can also instigate PDL1-independent resistance to ICB by altering the STAT1 epigenome. This effect can be stymied by the JAK1/JAK2 inhibitor.^654^ By further co-administering the JAK1/JAK2 inhibitor in populations manifesting ICB resistance, prolonged disease control may be fostered. Concurrently, the TME’s anomalous regulation of pathways, such as epigenetic silencing, can also be rectified through combination therapy^256,257,613^ (Fig. 7).

A topic worthy of discussion is whether inhibitors should be employed in cases where the activation of innate immune pathways exacerbates tumor progression. Our stance on this is cautious. Fundamentally, the pro-tumor function of innate immune pathways arises from the TME-driven activation of pro-tumor alternate routes.^12,211,642^ Yet at the molecular level, cells still retain the plasticity to revert to classical pathways. While inhibitors indeed curtail these pro-tumor bypasses, they also shut down the potential for reactivation of the innate immune pathway’s anti-tumor route. Thus, the use of inhibitors can be viewed as a compromise. A superior strategy would be to delve into the underlying mechanisms and intervention methods of these alternate bypasses. This could allow for the reprogramming of innate immune pathways to more effectively boost anti-tumor responses.

**Fig. 8:**
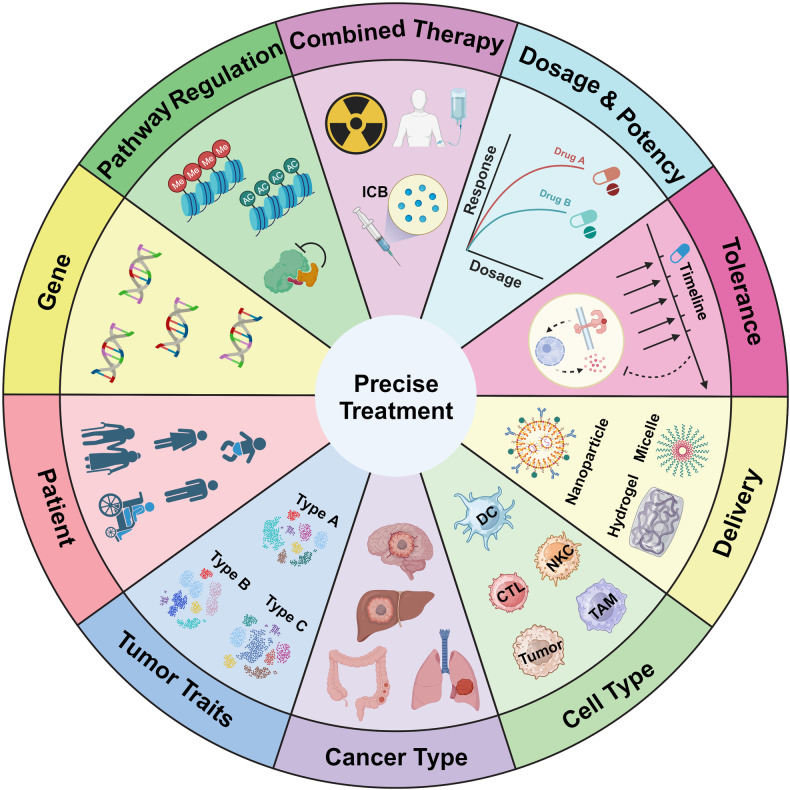
Precision treatment with innate immune pathway agonists. (1) Rational drug dosing and development of high-potency drugs. (2) Appropriate dosing regimens to overcome immune tolerance. (3) Efficient and precise delivery methods. (4) Selective targeting of specific cell types. (5) Tumor type and anatomical location. (6) Molecular and biochemical characteristics of the tumor. (7) Patient characteristics, including gender, age, health status, and medical history. (8) Patient’s genetic traits, especially genetic polymorphisms related to the innate immune pathway. (9) Correction of aberrant innate immune pathways. (10) Combination therapies to amplify therapeutic effects and avoid immune evasion. Created with BioRender.com

In summary, the key to overcoming the challenges of applying innate immune pathway agonists lies in the meticulous characterization of the tumor, patient, and drug, aiming for precision therapy (Fig. 8). All these factors contribute to the shaping of the innate immune pathway, resulting in diverse responses to innate immune activators.

## Conclusion

Targeting the innate immune pathway to remodel the tumor microenvironment and improve tumor prognosis is a captivating topic. The activation of the innate immune pathway, through the secretion of inflammatory cytokines and IFNs, can promote the recruitment and activation of immune cells, thus potentially inhibiting tumor growth. Numerous preclinical studies have already demonstrated its benefits. Our analysis indicates that immune cells in the TME undergo metabolic and immune reprogramming due to hypoxia, glucose deficiency, and various signaling molecules, losing their anti-tumor functions and acquiring pro-tumor phenotypes. This phenotypic transformation is reversible and can be reactivated against the tumor by targeting the innate immune pathway. However, the clinical translation of innate immune pathway activators is fraught with difficulties, with currently only three classes of drugs applied in a limited number of cancers. Moreover, recent studies suggest that activation of the innate immune pathway may promote tumor progression. In the TME, the innate immune pathway differs from normal physiological conditions, showing activation of alternative pathways and inhibition of classical pathways. This transformation could occur through various aspects of the central dogma, including epigenetic silencing of genes, post-translational modifications, and protein-protein interactions. Notably, whether this transformation occurs is related to tumor type and characteristics. For instance, the transformation of the STING pathway is closely related to the CIN properties of tumors. Investigating what tumor characteristics induce this transformation is one of the future research directions.

Additionally, different cell types display cell-specific intrinsic functions of the innate immune pathway. The role of this heterogeneity in tumor development is still under-researched. Current studies on alternative pathway activation are mainly focused on tumor cells themselves, with few studies addressing whether such transformations occur within innate immune cells. The lack of model animals capable of manipulating immune pathway molecules in specific cells may be a limiting factor in this field’s development. Currently, the role of intrinsic innate immune pathways in vivo is primarily based on two mouse models: the cre-loxp system and chimeric mouse models. Although these models allow for cell-type-specific pathway manipulation, their binary operation mode – the presence or absence of certain molecules – does not accurately reflect the changes in cell pathways in the TME, which mainly manifest as upregulation or downregulation. Establishing more refined models for continuous modulation may provide a deeper understanding of the role of these pathways in tumors.

**Fig. 9:**
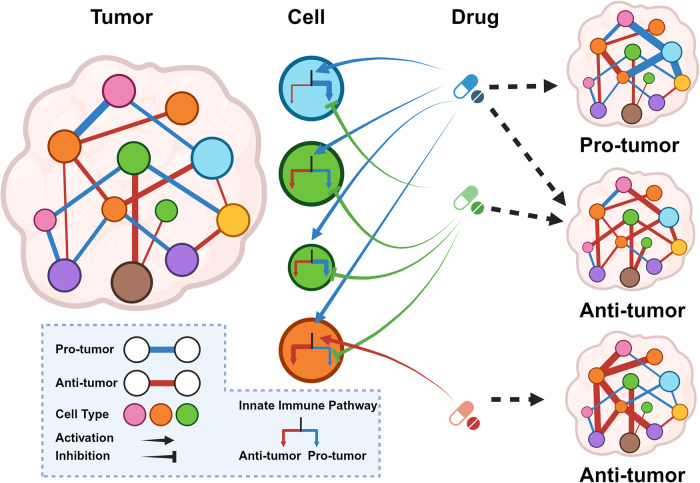
Targeted cell type-specific plasticity in the innate immune pathway network. In the tumor microenvironment, various types of cells construct a complex intercellular interaction network. These diverse cells exhibit heterogeneous innate immune pathways, with some cells predominantly featuring tumor-promoting pathways, while others are dominated by tumor-suppressing pathways. Additionally, the tumor background participates in shaping these pathways, meaning the same cell type can exhibit different innate immune pathway configurations in various microecological niches. Different targeting strategies may lead to distinct clinical outcomes: (1) Non-selective activation of innate immune pathways may cause a shift towards a tumor-promoting environment due to the dominance of immunosuppressive interactions; (2) A combination of non-selective innate immune pathway activators and tumor-promoting alternative pathway inhibitors can mediate changes across the entire network, potentially exerting anti-tumor effects; and (3) Cell type-specific innate immune pathway activators may induce localized anti-tumor actions. Created with BioRender.com

The analysis of intrinsic changes in the innate immune pathways of cells is key to promoting the clinical translation of targeted therapies for the innate immune pathway. Cells are the fundamental units of signaling pathway networks, with their membranes acting as barriers to create distinct entities for input and output. By analyzing these basic units and their pathways, we can construct a comprehensive signaling network for the entire tumor. This approach significantly advances our understanding of the tumor’s innate immune pathways and aids in the development of targeted drugs (Fig. 9).

The effect of targeted drugs on the tumor’s innate immune pathway is not static. They participate in reshaping the innate immune pathway, a typical example being the desensitization and tolerance to innate immune pathway agonists. The activation strength of the innate immune pathway can also impact the anti-tumor effect of downstream molecules, as previously discussed molecules like IL-12 and IFN-α have concentration-dependent characteristics, and changes in their concentration can induce opposing effects. Based on this, researching the intensity of targeted drugs and different dosing regimens’ impact on tumor prognosis will advance our utilization and transformation of existing drugs. The drug delivery method is also a factor affecting its therapeutic effect. On the one hand, the delivery method affects the effective drug concentration at the target; on the other hand, it determines the drug’s distribution pattern. The drug’s distribution pattern can affect its effectiveness, as the tumor is not a homogeneous entity. Many studies have revealed its heterogeneous micro-ecology, for instance, TAMs and TRMs occupying different spatial positions and performing different functions. Developing precisely targeted drugs will help eliminate interference and optimize therapeutic effects.

Recent discoveries in the innate immune pathway have also spawned new directions of development, with nuclear innate immune pathway sensors being one representative. These nuclear DNA and RNA sensors may more sensitively detect the genomic instability of tumors, playing a key role in the early stages of tumor development. Targeting these points could fully exploit this weakness of tumors. Some nuclear sensors, like SAFA may allow targeted effects to bypass the reorganized cytoplasmic signaling networks, achieving sustained and precise therapeutic effects. Microbiome regulation is another interesting direction, as it offers a relatively safe and convenient intervention mode. It can be modulated through diet, lifestyle, and other means, and the association of the gut microbiome with various systems offers the possibility of intervening in the gut microbiome to improve the prognosis of distant tumors.

In conclusion, a precise analysis of the innate immune pathway is crucial for bridging the gap between the preclinical and clinical stages of cancer treatment. This analysis necessitates a collaborative effort that encompasses but is not limited to, molecular biology, systems biology, immunology, and oncology. With the advent of artificial intelligence, building models based on these interdisciplinary data can greatly enhance our ability to predict drug efficacy and accelerate drug development.
